# Myocardial revascularization in patients with chronic kidney disease: a systematic review and meta-analysis of surgical versus percutaneous coronary revascularization

**DOI:** 10.1093/icvts/ivaf021

**Published:** 2025-02-19

**Authors:** Valentina Grazioli, Michele Di Mauro, Giacomo Perocchio, Piersilvio Gerometta, Alfonso Agnino, Maurizio Pin, Paolo Meani, Matteo Matteucci, Daniele Ronco, Giulio Massimi, Jos Maessen, Domenico Corradi, Mario Gaudino, Roberto Lorusso

**Affiliations:** Cardio-Thoracic Surgery Department, Maastricht University Medical Centre (MUMC), Maastricht, The Netherlands; Department of Cardiac Surgery, Cliniche Humanitas Gavazzeni, Bergamo, Italy; Faculty of Health, Medicine and Life Sciences, Maastricht University, Maastricht, The Netherlands; Division of Cardiac Surgery, Ospedale Policlinico San Martino, Genova, Italy; Department of Cardiac Surgery, Cliniche Humanitas Gavazzeni, Bergamo, Italy; Department of Cardiovascular Surgery, Division of Robotic and Minimally-Invasive Cardiac Surgery, Cliniche Humanitas Gavazzeni, Bergamo, Italy; Cardiovascular Department, Maria Cecilia Hospital, GVM Care & Research, Cotignola (Ravenna), Italy; Cardio-Thoracic Surgery Department, Maastricht University Medical Centre (MUMC), Maastricht, The Netherlands; Cardio-Thoracic Surgery Department, Maastricht University Medical Centre (MUMC), Maastricht, The Netherlands; Cardiac Surgery Unit, ASST Sette Laghi, Varese, Italy; Cardio-Thoracic Surgery Department, Maastricht University Medical Centre (MUMC), Maastricht, The Netherlands; Cardiac Surgery Unit, ASST Grande Ospedale Metropolitano Niguarda, Milano, Italy; Cardio-Thoracic Surgery Department, Maastricht University Medical Centre (MUMC), Maastricht, The Netherlands; Cardiac Surgery Unit, Santa Maria Hospital, Terni, Italy; Cardio-Thoracic Surgery Department, Maastricht University Medical Centre (MUMC), Maastricht, The Netherlands; Cardiovascular Research Institute Maastricht (CARIM), Maastricht, The Netherlands; Department of Medicine and Surgery, Unit of Pathology, University of Parma, Parma, Italy; Department of Cardiothoracic Surgery, Weill Cornell Medicine, Presbyterian Hospital, New York, NY, USA; Cardio-Thoracic Surgery Department, Maastricht University Medical Centre (MUMC), Maastricht, The Netherlands; Cardiovascular Research Institute Maastricht (CARIM), Maastricht, The Netherlands

**Keywords:** chronic kidney disease, coronary artery bypass graft, percutaneous coronary intervention, major adverse cardiovascular and cerebrovascular events

## Abstract

**OBJECTIVES:**

To compare outcomes of two different revascularization strategies in chronic kidney disease (CKD) patients: coronary artery bypass grafting (CABG) versus percutaneous coronary intervention (PCI).

**METHODS:**

We conducted this meta-analysis according to Preferred Reporting Items for Systematic Review and Meta-Analyses guidelines and registered with PROSPERO (CRD42021238659), evaluated studies comparing CABG and PCI in patients with CAD and CKD (defined by KDIGO guidelines). Data were extracted from PubMed, EMBASE and Cochrane from 2000 to 2023. The primary end-point was long-term major adverse cardiovascular and cerebrovascular event rates, with secondary end-points including 30-day mortality, stroke, myocardial infarction (MI) and repeat revascularization. Statistical analyses included Kaplan–Meier estimations, Cox regression, and meta-regression to address heterogeneity. Publication bias was assessed via funnel plots. No funding was received, and the authors report no conflicts of interest.

**RESULTS:**

We included 33 studies with 402 300 patients (eGFR <60 ml/min/1.73 m^2^). The cohort comprised 132 314 coronary artery bypass graft and 269 986 PCI patients. Over 3 years, coronary artery bypass group provided protection against major adverse cardiac and cerebrovascular events, MI, and repeat revascularization compared to PCI. However, PCI showed better short-term outcomes, including lower 30-day mortality. Coronary artery bypass group was linked to a higher stroke risk over the 3-year follow-up.

**CONCLUSIONS:**

Revascularization strategies for CKD and coronary artery disease patients should balance PCI's short-term benefits with CABG’s long-term advantages.

## GRAPHICAL ABSTRACT

**Figure:**
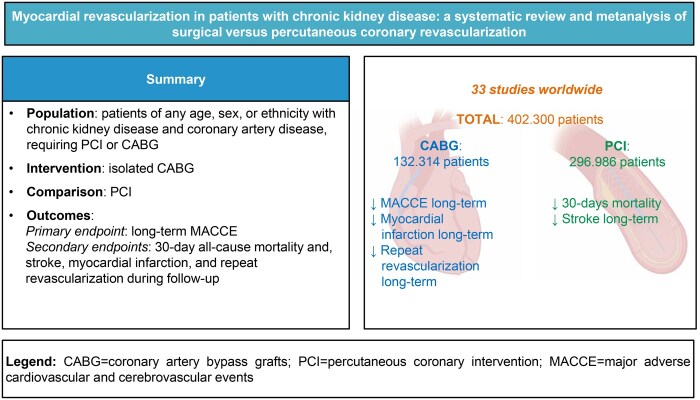

## INTRODUCTION

Chronic kidney disease (CKD) represents a global healthcare challenge with a rising incidence and affected more than 10% of the global population [1–3]. CKD is intricately linked to cardiovascular disease (CVD), and coronary artery disease (CAD) represents the most common illness in such a setting [1, 2, 4, 5].

In CKD, both traditional (e.g. diabetes and hypertension) and non-traditional CKD-related cardiovascular factors (e.g. abnormalities in calcium-related metabolism, anemia, inflammation, oxidative stress and dialysis-related factor) may contribute to the development of severe cardiovascular complications [4, 6]. Indeed, coronary arteries in CKD patients presents more marked calcification compared to non-CKD patients, typically involving both the intimal and medial layers, determining a more aggressive disease compared to non-CKD patients [4, 6–10].

The optimal therapeutic strategy for CAD in CKD remains still controversial [1]. The 2018 ESC/EACTS guidelines on myocardial revascularization reaffirm the prior ESC recommendation favoring coronary artery bypass grafting (CABG) for patients with a life expectancy exceeding 1 year [1, 11]. As a limitation, these guidelines, as well as the majority of the literature related to this topic, are primarily composed of observational trials [1].

To clarify and investigate the actual outcomes of CKD patients undergoing myocardial revascularization, and particularly to better understand the optimal revascularization strategy between surgery (CABG) and percutaneous coronary intervention (PCI) for these patients, we performed a systematic review and meta-analysis of studies presented in the literature, that are primarily observational, compare the outcomes of CABG and PCI in patients with CKD.

## MATERIALS AND METHODS

This systematic review and meta-analysis was performed according to the Preferred Reporting Items for Systematic Review and Meta-Analyses (PRISMA) statement and the PRISMA checklist can be found in Supplementary Material (Table S1) [12].

### Search strategy

We searched PubMed, EMBASE, and the Cochrane Central Register of Controlled Trials from January 2000 to December 2023 using this algorithm: (‘coronary artery disease’ AND (‘chronic kidney disease’ OR ‘kidney disease’ OR ‘renal disease’ OR ‘end stage renal disease’ OR ‘renal insufficiency’ OR ‘renal failure’ OR ‘kidney failure’ OR ‘dialysis’)) AND ((‘coronary artery bypass graft’ OR ‘coronary artery bypass grafting’ OR ‘coronary artery bypass surgery’ OR ‘coronary artery bypass’) AND (‘percutaneous coronary intervention’ OR ‘balloon angioplasty’ OR ‘transluminal coronary angioplasty’ OR ‘coronary angioplasty’ OR ‘percutaneous transluminal coronary angioplasty’)).

The search was limited to English-language articles. References from selected articles and reviews were also examined. This systematic review and meta-analysis was registered with PROSPERO (CRD42021238659). The study received no funding, and the authors declare no conflicts of interest.

### Study selection

Eligible studies met the following PICOS criteria: (i) Population: patients of any age, sex or ethnicity with CAD and CKD requiring PCI or isolated CABG (on- or off-pump). CKD was defined according to the 2017 KDIGO Guidelines [1, 13]: G1 (GFR ≥ 90 ml/min/1.73 m^2^), G2 (GFR 60–89 ml/min/1.73 m^2^), G3 (GFR 30–59 ml/min/1.73 m^2^), G4 (GFR 15–29 ml/min/1.73 m^2^), and G5 (GFR < 15 ml/min/1.73 m^2^). We also used the groupings suggested by Milojevic *et al.*, combining G3–G5 stages for moderate to severe CKD [1]. (ii) Intervention: CABG, either on- or off-pump, using venous and/or arterial grafts. (iii) Comparison: PCI with stent: drug-eluting stents (DES); bare-metal stent (BMS); everolimus-eluting stent (EES). Studies were included if they reported at least one outcome of interest.

We excluded studies published as abstracts, conference presentations, brief communications, reviews, meta-analyses, or case reports with only 1 or 2 cases. Two independent investigators (V.G. and G.P.) reviewed the selected studies to ensure they met the predefined inclusion criteria, with any disagreements resolved through consensus.

### Data extraction and quality assessment

Two authors (V.G. and G.P.) independently extracted data using a standardized form to ensure uniformity across studies. They recorded study design, patient characteristics, quality indicators, and clinical outcomes. Discrepancies were resolved with a third investigator (A.S.). Two reviewers assessed each study’s risk of bias using the Newcastle-Ottawa Scale (NOS) [14].

### Endpoints

The primary end-point was the long-term rate of major adverse cardiovascular and cerebrovascular events (MACCE), including all-cause death, stroke, myocardial infarction (MI), and repeat revascularization. For this end-point we selected study that presented Kaplan–Meier curve. Secondary end-points were 30-day all-cause mortality and, stroke, MI, and repeat revascularization during follow-up (3 years). Endpoints were defined according to each study’s report.

### Statistical analysis

MACCE data were extracted from Kaplan–Meier curves digitized using DigitizeIt 2.5.9 software, with survival data obtained via Guyot’s algorithm [15].

The quality of Kaplan–Meier-derived individual patient data was verified by comparing derived curves with the originals.

The Kaplan–Meier estimator and log-rank test were used to compare MACCE incidence between PCI and CABG. A Cox regression model, stratified by study, was applied to analyze survival data, using CABG as the reference group. The resulting Kaplan–Meier survival curves were plotted.

We estimated the 60-month restricted mean survival time difference between CABG and PCI for composite MACCE-free survival [16].

A sensitivity analysis on MACCE was conducted excluding studies with patients operated before the year 2000.

Early outcomes were reported as odds ratios (OR) with 95% confidence intervals (CI) using the Mantel-Haenszel method. Heterogeneity was assessed with the *I*^2^ statistic, categorized as low (<25%), moderate (25–50%) or high (>50%), and Cochran’s Q test. A random-effects model (tau2) was used to account for inter-study variance, with tau2 = 0 indicating no heterogeneity. Publication bias was assessed through funnel plot inspection.

Leave-one-out sensitivity analyses were performed for all outcomes. Due to substantial heterogeneity, the effects of clinical variables (hypertension, diabetes, dialysis) and methodological variables (year of publication, study type) were explored using multiple linear meta-regression models. Regression coefficients were reported with 95% confidence intervals (CI). A *P*-value of 0.05 was considered statistically significant. All analyses were conducted using R Statistical Software (version 4.2.1; R Foundation for Statistical Computing, Vienna, Austria).

## RESULTS

**Figure 1:**
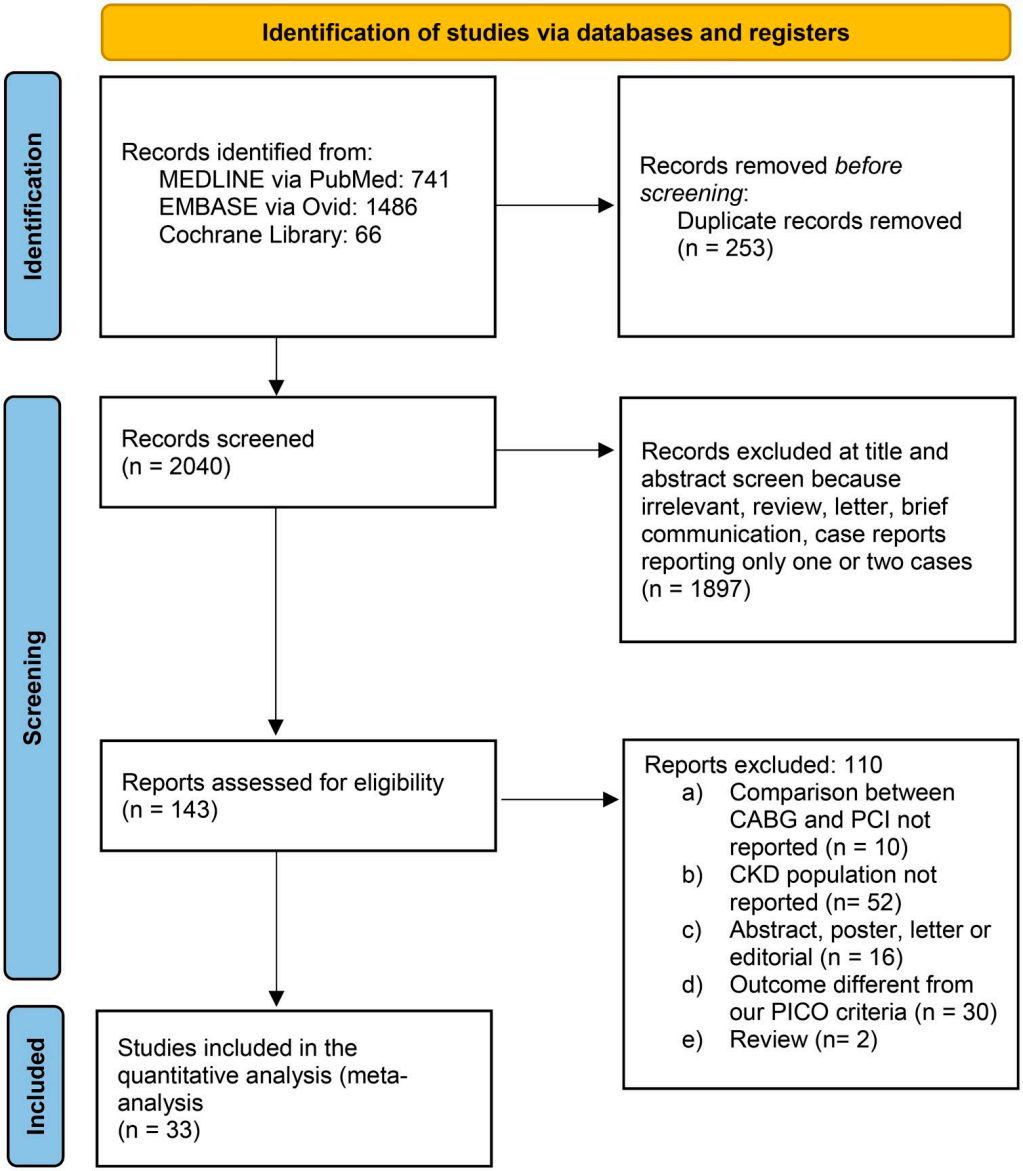
PRISMA flow-chart diagram for the selection of papers. Page MJ, et al. BMJ 2021;372:n71. doi: 10.1136/bmj.n71

Thirty-three studies were included in our analysis, as illustrated in Fig. 1 (PRISMA flow chart) [1, 17–47]. Of these, 24 were observational retrospective or cohort studies, and 9 used propensity-matched analysis. Geographically, 15 studies were in Asia, 7 in the USA, 1 in Canada, 2 in South America, 1 in Europe, 3 in Australia/New Zealand and 4 were international multicenter studies. All studies involved patients with eGFR <60 ml/min/1.73 m^2^, with 12 focusing on dialysis patients. The cohort included 402 300 patients, with 132 314 in the CABG group and 269 986 in the PCI group. Key study and patient characteristics are listed in Table 1.

**Table 1:** Main Characteristics of eligible studies and patients’ demographics

	Region	Period	Design	Patients	Age	Male (%)	Diabetes	Dyslipidaemia	Hypertension	Smoker	Stent type	FUP, years
				CABG/PCI	CABG/PCI	CABG/PCI	CABG/PCI	CABG/PCI	CABG/PCI	CABG/PCI		CABG/PCI
Agirbasli *et al.* 2000 [17]	Georgia	1987–97	Observational	130/122	60 ± 10.6/58 ± 13.03	84 (65%)/78 (64%)	54 (46%)/68 (57%)	44 (51%)/48 (45%)	108 (91%)/113 (95%)	17 (16%)/24 (21%)	n/a	1
Aoki *et al.* 2005 [18]	International multicentre	1997–8	*Post hoc* RCT	73/69	71 ± 6/70 ± 6	53 (73%)/44 (64%)	11(15%)/15 (22%)	38 (52%)/40 (58%)	34 (47%)/36 (52%)	8 (11%)/11 (16%)	n/a	5
Bangalore *et al.* 2015 [19]	New York	2008–11	PSM	2960/2960	69.62 ± 10.53/69.94 ± 10.67	61.5%/62.0%	48.3%/48.9%	63.3%/62.4%	78.2%/77.0%	26.8%/26.4%	EES	2.9
Chan *et al.* 2015 [20]	Ontario	2008–11	PSM	893/893	75.0 ± 7.5/75.2 ± 9.4	464 (52.0%)/477 (53.4%)	403 (45.1%)/386 (43.2%)	672 (75.3%)/671 (75.1%)	760 (85.1%)/741 (83.0%)	434 (48.6%)/438 (49.1%)	DES	3
Chang *et al.* 2013 [21]	California	1996–2008	Observational	1458/1458	GFR 45–59: 70.6(7.9)/70.7 (8.0)	n/a	GFR 45–59: 30.8%/30.8%	GFR 45–59: 88.8%/87.8%%	GFR 45–59: 68.0%/68.7%	GFR 45–59: 31.8%/33.2%	n/a	5
					GFR < 45: 74.5(7.5)/74.4 (7.5)		GFR < 45: 44.3%/44.3%	GFR < 45: 88.0%/89.0%	GFR < 45: 83.8%/80.5%	GFR < 45: 33.8%/33.3%		
Charytan *et al.* 2021 [22]	Massachusetts	2003–12	PSM	3775/3775	73.0/72.9	2419 (64.1%)/2385 (63.2%)	1801 (47.7%)/1748 (46.3%)	3332 (88.3%)/3325 (88.1)	3424 (90.7)/3448 (91.3)	452 (12.0)/432 (11.4)	n/a	5.4
Giustino *et al.* 2018 [23]	International mutlicenter		*Post hoc* RCT	184/177	72.7 ± 7.8	239 (66.2%)	146 (40.4%)	266 (73.9%)	306 (84.8)	44 (12.3)	EES	3
Ix *et al.* 2005 [24]	International mutlicenter	1997–8	*Post hoc* RCT	139/151	69 ± 7/68 ± 6	56%/57%	16%/20%	57%/54%	54%/47%	9%/17%	n/a	3
Kang *et al.* 2017 [25]	Korea	2003–13	Observational	943/1165	66.4 ± 8.4/67.3 ± 9.5	661 (70.1%)/779 (66.9%)	547 (58%)/574 (49.3%)	334 (35.4%)/402 (34.5%)	716 (75.9%)/884 (75.9%)	418 (44.3%)/486 (41.7%)	DES	5
Kilic *et al.* 2020 [26]	Pittsburgh	2010–17	PSM	352/352	72.0 (67.0–79.0)/73.0(65.0–81.0)	220 (62.5%)/205 (58.2%)	201 (57.1%)/203 (57.7%)	317 (90.1%)/313 (88.9%)	330 (93.8%)/331 (94.0%)	58 (16.5%)/60 (17.1%)	n/a	3.2
Kim *et al.* 2020 [27]	Korea	2003–17	Observational	355/536	GFR 30–60:	GFR 30–60:	GFR 30–60:	GFR 30–60:	GFR 30–60:	GFR 30–60:	DES	2
					71 (64–76)	622 (74.2%)	388 (46.3%)	487 (58.1%)	636 (75.9%)	177 (21.1%)		
					GFR < 30:	GFR < 30:	GFR < 30:	GFR < 30:	GFR < 30:	GFR < 30:		
					69 (62–74)	163 (70.3%)	180 (77.6%)	125 (53.9%)	215 (92.7%)	36 (15.5%)		
Koh *et al.* 2023 [28]	Singapore	2013–17	Observational	46/274	57.5 ± 10.9/63.6 ± 10.2	30 (65.2%)/185 (67.5%)	34 (73.9%)/184 (67.2%)	38 (82.6%)/225 (82.1%)	43 (93.5%)/264 (96.4%)	4 (8.7%)/25 (9.1%)	n/a	1
Komiya *et al.* 2015 [29]	Japan	2005–7	PSM	77/77	72.7 ± 7.1/72.7 ± 9.4	44 (57%)/48 (62%)	44 (57%)/49 (64%)	39 (51%)/35 (45%)	73 (95%)/70 (91%)	17 (22%)/16 (21%)	DES	2.4
Kumada *et al.* 2018 [30]	Japan	2010–14	PSM	92/92	66 ± 9/69 ± 9	79.7%/74.8%	58.0%/69.5%	24.6%/33.2%	65.9%/74.9%	24.6%/28.3%	EES	3.6
Lautamaki *et al.* 2016 [31]	Finland	2007–10	PSM	54/54	72.0 ± 8.5/72.5 ± 10.4	30 (55.6%)/29 (53.7%)	24 (44.4%)/30 (55.6%)	n/a	37 (68.5%)/46 (85.2%)	n/a	n/a	2
Lima *et al.* 2016 [32]	Brazil	1995–2010	Observational	46/40	67 ± 7/68 ± 6	65.2%/52.5%	n/a	n/a	71.7%/77.5%	13.0%/2.5%	BMS/DES	5.4
Lin, 2018 [50]	Taiwan	2004–10	Observational	101/84	72.8 ± 10/74.2 ± 10	83 (82.2%)/72 (85.7%)	53 (52.5%)/46 (54.8%)	39 (39%)/44 (52%)	87 (86.1%)/74 (88.1%)	71 (70%)/38 (45%)	BMS/DES	3.5
Lopes *et al.* 2009 [33]	Brazil	n/a	*Post hoc* RCT	46/49	67	101 (67%)	90 (60%)	n/a	57 (38%)	111 (74%)	n/a	5
Manabe *et al.* 2009 [34]	Japan	2004–7	Observational	28/18	63.9 ± 8.9/61.2 ± 12.2	23/17	18/9	6/6	20/14	14/6	BMS/DES	1.29 ± 0.7/1.48 ± 0.8
Marui *et al.* 2014 [35]	Japan	2005–7	Observational	130/258	66.5 ± 8.7/66.2 ± 10.6	104 (80%)/187 (73%)	77 (59%)/167 (65%)	n/a	107 (82%)/229 (89%)	20 (15%)/46 (18%)	BMS/DES	4.9
Milojevic *et al.* 2018 [1]	International multicentre	n/a	*Post hoc* RCT	151/158	71.6 ± 7.9/71.9 ± 7.3	101 (66.9%)/108 (68.4%)	50 (33.1%)/44 (27.8%)	119 (79.3%)/122 (77.7%)	128 (85.3%)/135 (86%)	n/a	DES	5
Pan *et al.* 2016 [36]	China	2005–10	Observational	206/147	68.4 ± 5.7/69.3 ± 5.5	155 (75.2%)/108 (73.5%)	74 (35.9%)/60 (40.8%)	32 (26.0%)/8 (10.0%)	86 (69.9%)/52 (65.0%)	61 (49.6%)/29 (36.3%)	DES	2.5
Pan *et al.* 2023 [37]	Taiwan	2008–17	PSM	920/920	61.7 ± 9.3/62.2 ± 9.8	621(67.5%)/628(68.3%)	506(55%)/502(54.6%)	281(30.5%)/265(28.8%)	716(77.8%)/715(77.7%)	n/a	DES	2.8 (1.2–4.4)
Pilmore *et al.* 2017 [38]	New Zeland	2003–12	Observational	30/61	58.4 ± 8.4/61.2 ± 12.2	22 (73%)/41 (67%)	19 (63%)/34 (56%)	n/a	n/a	8 (27%)/26 (43%)	n/a	3.3
Sattar *et al.* 2020 [39]	Pakistan	2012–16	Observational	119/109	63.1 ± 10.0/65.4 ± 11.6	74 (62.2%)/70 (64.2%)	86 (72.3%)/67 (61.5%)	38 (31.9%)/44 (40.4%)	97 (81.5%)/87 (79.8%)	14 (11.8%)/17 (15.6%)	DES	1.8
Shroff *et al.* 2013 [40]	United States	2004–10	Observational	6178/16855	n/a	62.2%/55.4%	75.2%/75.8%	n/a	n/a	n/a	DES/BMS	1.6 (0.5–2.9)/1.6 (0.7–2.9)+0.9 (0.5–2.2)
Sugumar *et al.* 2014 [41]	Australia and New Zeland	2004–8	Observational	526/263	GFR 30–60: 75.2 ± 8.6/73.7 ± 9.0	GFR 30–60: 56.9%/61.1%	GFR 30–60: 36.3%/36.3%	GFR 30–60: 68.6%/72.1%	GFR 30–60: 82.1%/81.4%	GFR 30–60: 9.5%/9.3%	n/a	GFR 30–60: 3.2 ± 1.3
					GFR < 30: 68.7 ± 11.7/69.3 ± 11.4	GFR < 30: 63.5%/70.3%	GFR < 30: 33.8%/46%	GFR < 30: 74.3%/73.0%	GFR < 30: 83.8%/86.5%	GFR < 30: 14.9%/16.2%		GFR < 30: 2.8 ± 1.6
Sunagawa *et al.* 2010 [42]	Japan	2002–6	Observational	29/75	62.9 ± 10.1/65.2 ± 12.6	87%/73%	43%/43%	20%/20%	80%/77%	n/a	DES	2.6/2
Terazawa *et al.* 2012 [43]	Japan	2004–7	Observational	58/67	65 ± 8.2/63.6 ± 9.3	45 (78%)/51 (76%)	30 (52%)/43 (64%)	n/a	45 (78%)/52 (78%)	n/a	DES	3
Ullah *et al.* 2021 [44]	United States	2002–17	Observational	112099/238524	65.1 ± 11.2/64.9 ± 11.7	74313 (66.3%)/139729 (58.6%)	26637 (23.8%)/71951 (30.3%)	n/a	70984 (63.5%)/155070 (65.2%)	n/a	n/a	n/a
Wang*et al.* 2020 [45]	China	2007–17	Observational	26/86	63.6 ± 10.8/62.2 ± 11.3	76.9%/67.4%	50.0%/54.7%	n/a	96.2%/96.5%	23.1%/11.8%	DES	1.6
Yeates *et al.* 2012 [46]	Australia	1999–2009	Observational	24/31	60.3 ± 9.3/58.5 ± 11.1	14 (58.3%)/21 (67.7%)	10 (41.7%)/14 (45.2%)	20 (83%)/26 (84%)	22 (91%)/31 (100%)	3 (12.5%)/0 (0%)	n/a	2
Zhang *et al.* 2016 [47]	China	2009–14	Observational	66/86	63.3 ± 5.9	129 (61.4%)	116 (55.2%)	50 (23.8%)	138 (65.7%)	75 (35.7%)	n/a	3.2

FUP, follow-up period; CABG, coronary artery bypass graft; PCI, percutaneous coronary intervention; RCT, randomized clinical trial; PSM, propensity score matching; GFR, glomerular filtration rate; DES, drug eluting stent; EES, everolimus-eluting stent; BMS, bare-metal stent; n/a, not applicable.

### Primary end-point

**Figure 2:**
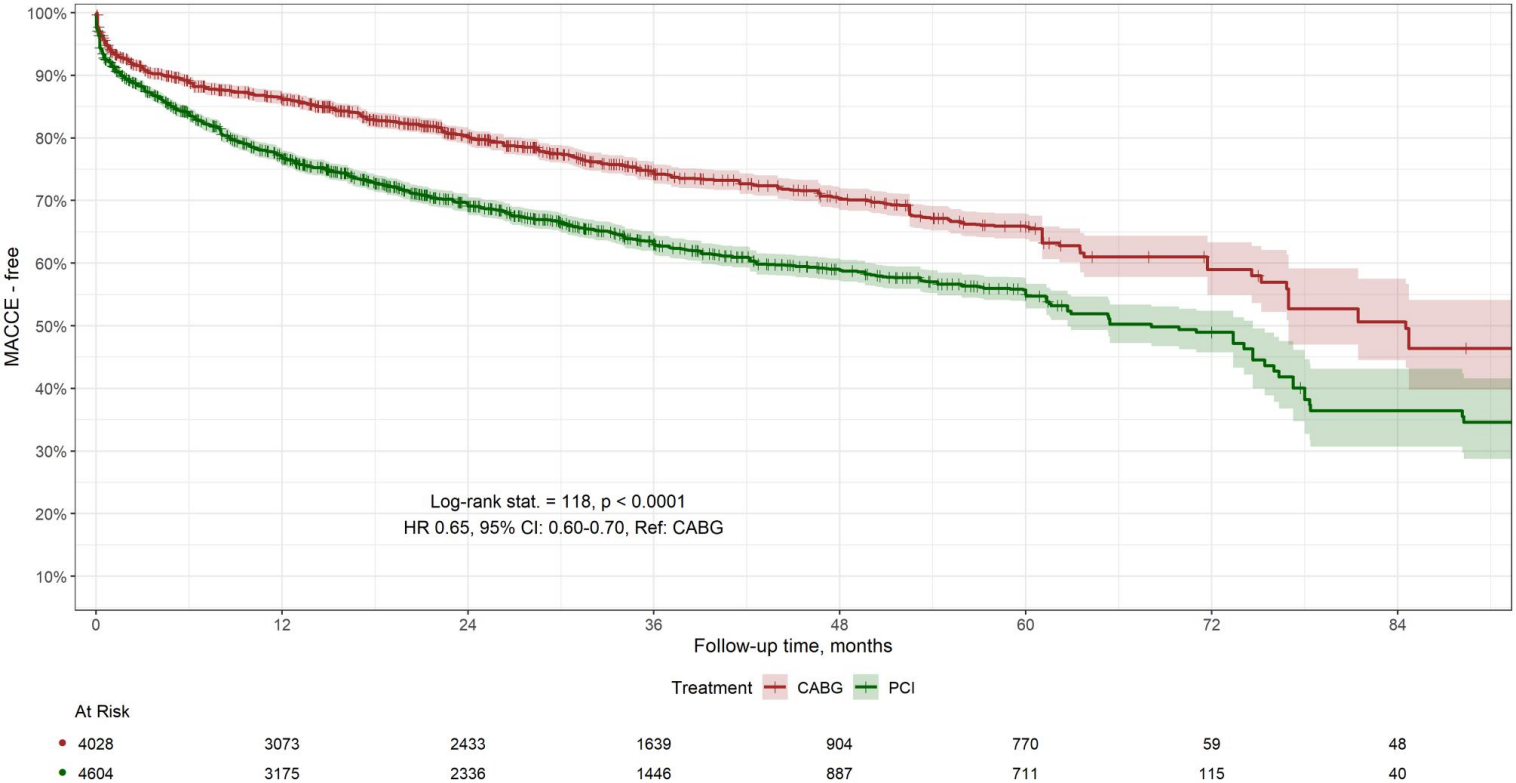
MACCE-free survival curves of CABG versus PCI

A total of 20 studies, involving 8632 patients, provided MACCE-free survival data over a follow-up period of 0–120 months. At 12, 24, 36, 48 and 60 months, MACCE-free survival rates were higher in the CABG group (86%, 80%, 74%, 70% and 65%) compared to the PCI group (77%, 69%, 63%, 59% and 55%). The survival curves showed significant differences (log-rank test, chi-square 118, *P* < 0.0001) (Fig. 2). The Hazard Ratio (HR) was 0.65 (95% CI, 0.60–0.70), indicating an association of PCI with adverse events. CABG improved MACCE-free survival by 5.92 months over 60 months (95% CI, −6.95 to −4.88, *P* < 0.0001).

Sensitivity analysis on MACCEs excluding studies with patients operated before the year 2000, involving 8105 patients showed consistent results compared to the overall study findings. The survival curves showed significant differences (log-rank test, chi-square 114, *P* < 0.0001) (Supplementary Fig. S1), and the HR was 0.64 (95% CI, 0.59–0.69).

### Secondary end-points

**Figure 3:**
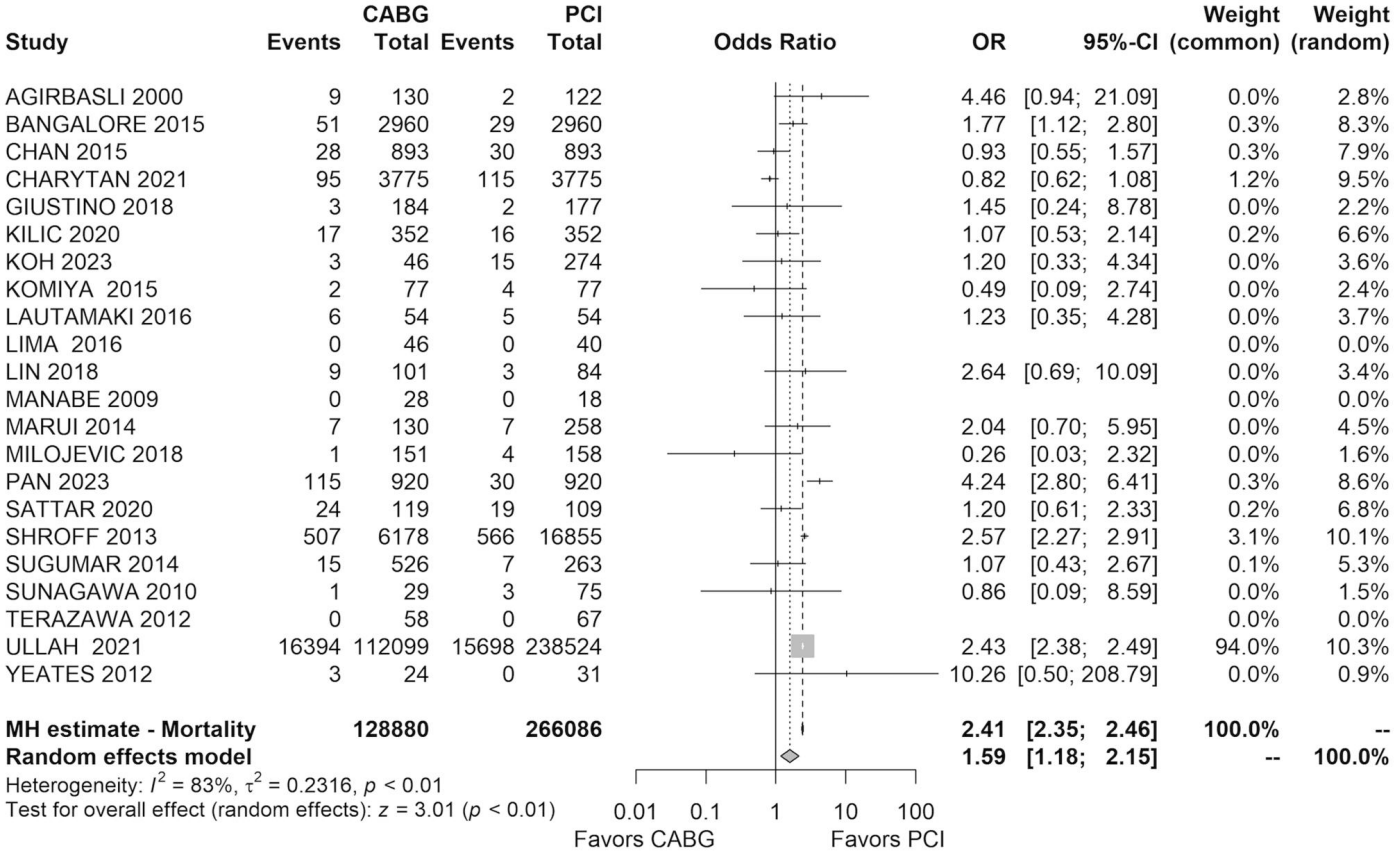
Forest plot of the early mortality for CABG versus PCI

Thirty-day mortality was reported for 395 755 patients across 22 studies, with rates of 13% in the CABG group and 6% in the PCI group. CABG had significantly higher early mortality (OR = 1.59, 95% CI: 1.18–2.15, *I*^2^=83%, *τ*^2^=0.23) (Fig. 3).

**Figure 4:**
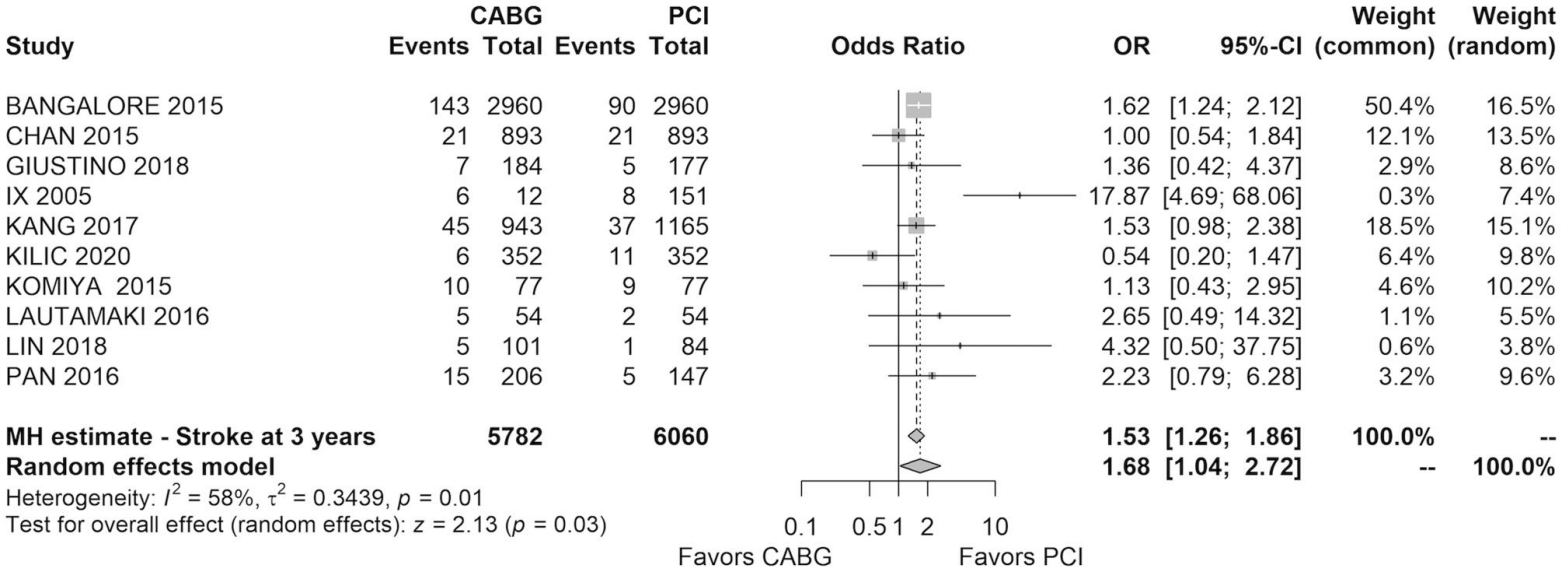
Forest plot of the long-term stroke rate (3 years) for CABG versus PCI

Ten studies reported stroke incidence at follow-up, involving 11 969 patients. At 3 years, stroke occurred in 4.5% of the CABG group and 3.1% of the PCI group. CABG was associated with a significantly higher stroke incidence (OR = 1.68, 95% CI: 1.04–2.72, *I*^2^=58%, *τ*^2^=0.34; Fig. 4).

**Figure 5:**
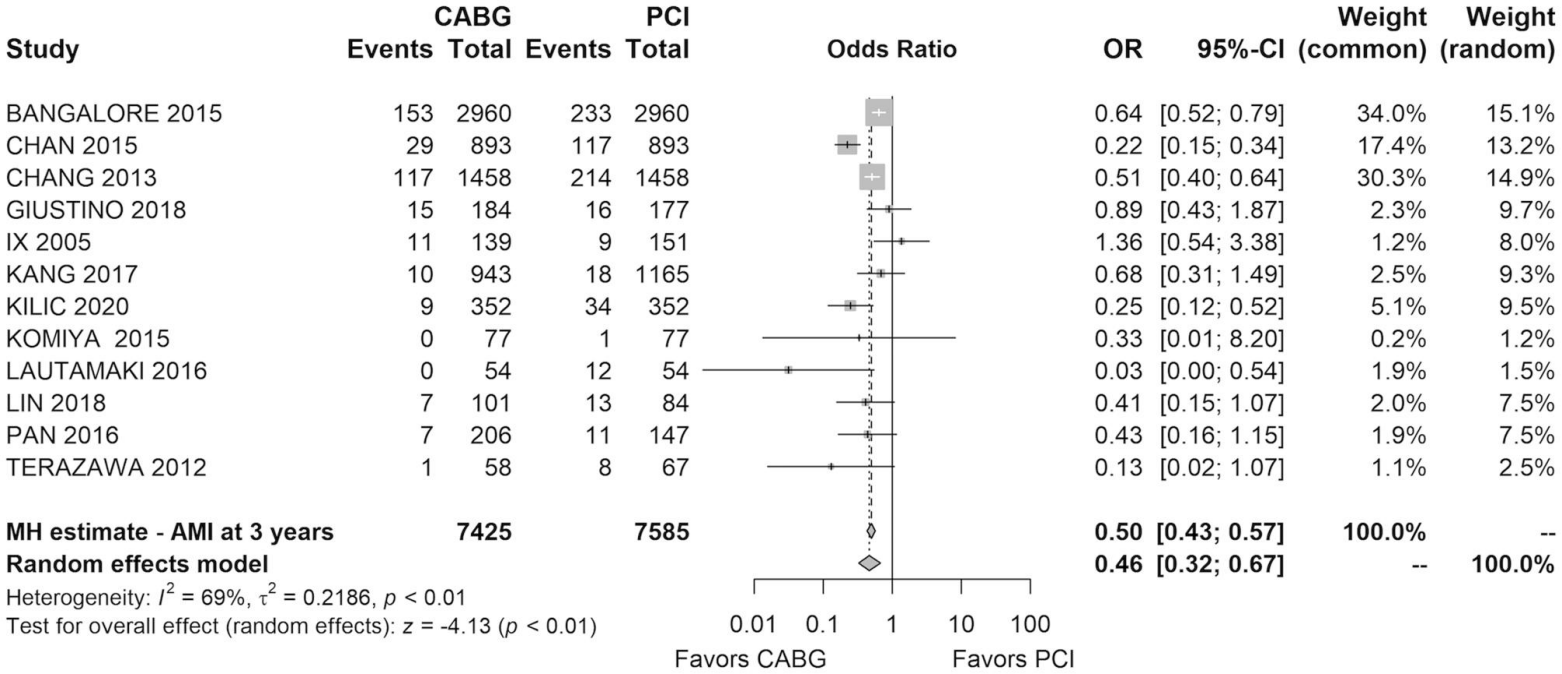
Forest plot of the MI rate at follow-up period of 3 years for CABG versus PCI

Data on MI rates at follow-up were extracted from 12 studies involving 15 010 patients. At 3 years, the CABG group had a 4.8% MI rate compared to 9% in the PCI group. PCI was associated with a significantly higher MI incidence (OR = 0.46, 95% CI: 0.32–0.67, *I*^2^=69%, *τ*^2^=0.22). Figure 5 shows the forest plot for MI at follow-up.

**Figure 6:**
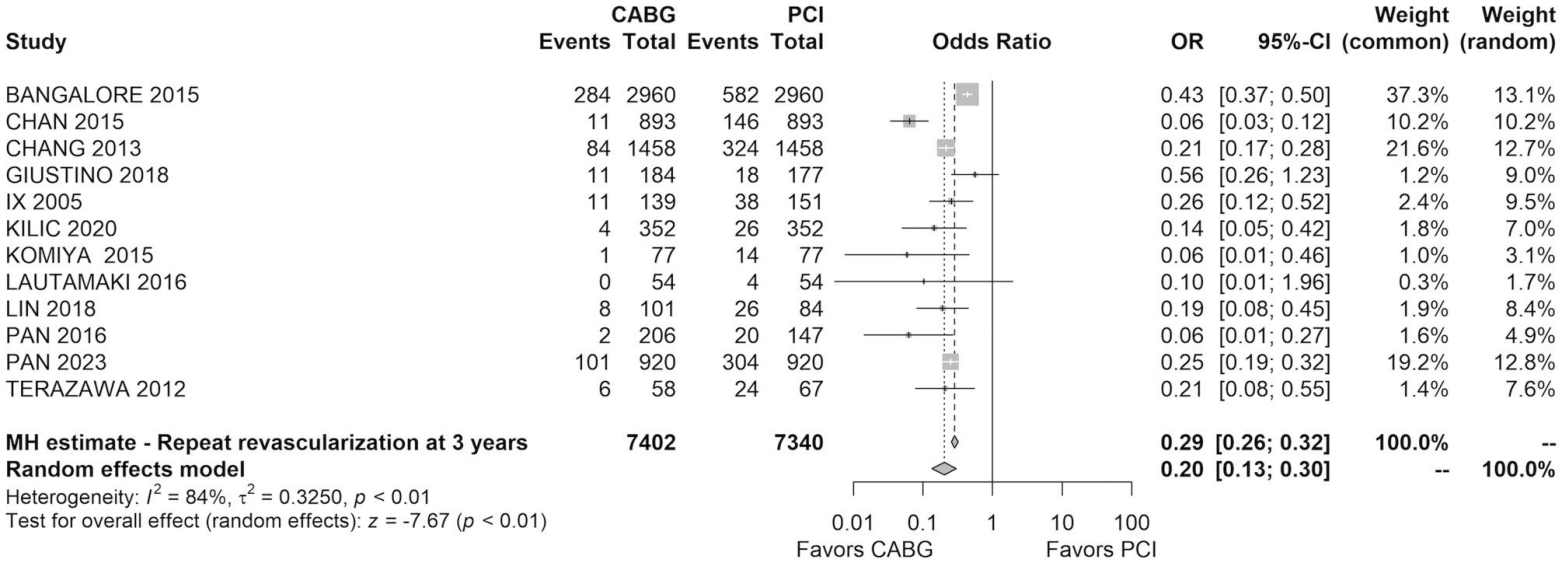
Forest plot of the repeat revascularization rate at follow-up period of 3 years for CABG versus PCI

Twelve studies, involving 14 742 patients, reported repeat revascularization rates at 3 years of 7% in the CABG group versus 21% in the PCI group. PCI was associated with a significantly higher incidence of repeat revascularization (OR = 0.20, 95% CI: 0.13–0.30, *I*^2^ = 84%, *τ*^2^ = 0.33) as shown in Fig. 6.

For all 30-day mortality, stroke, MI, and repeat revascularization, no small study effect was noted (Supplementary Figs S2, S4, S6, and S8), and leave-one-out analysis confirmed stable results (Supplementary Figs S3, S5, S7, and S9).

**Figure 7:**
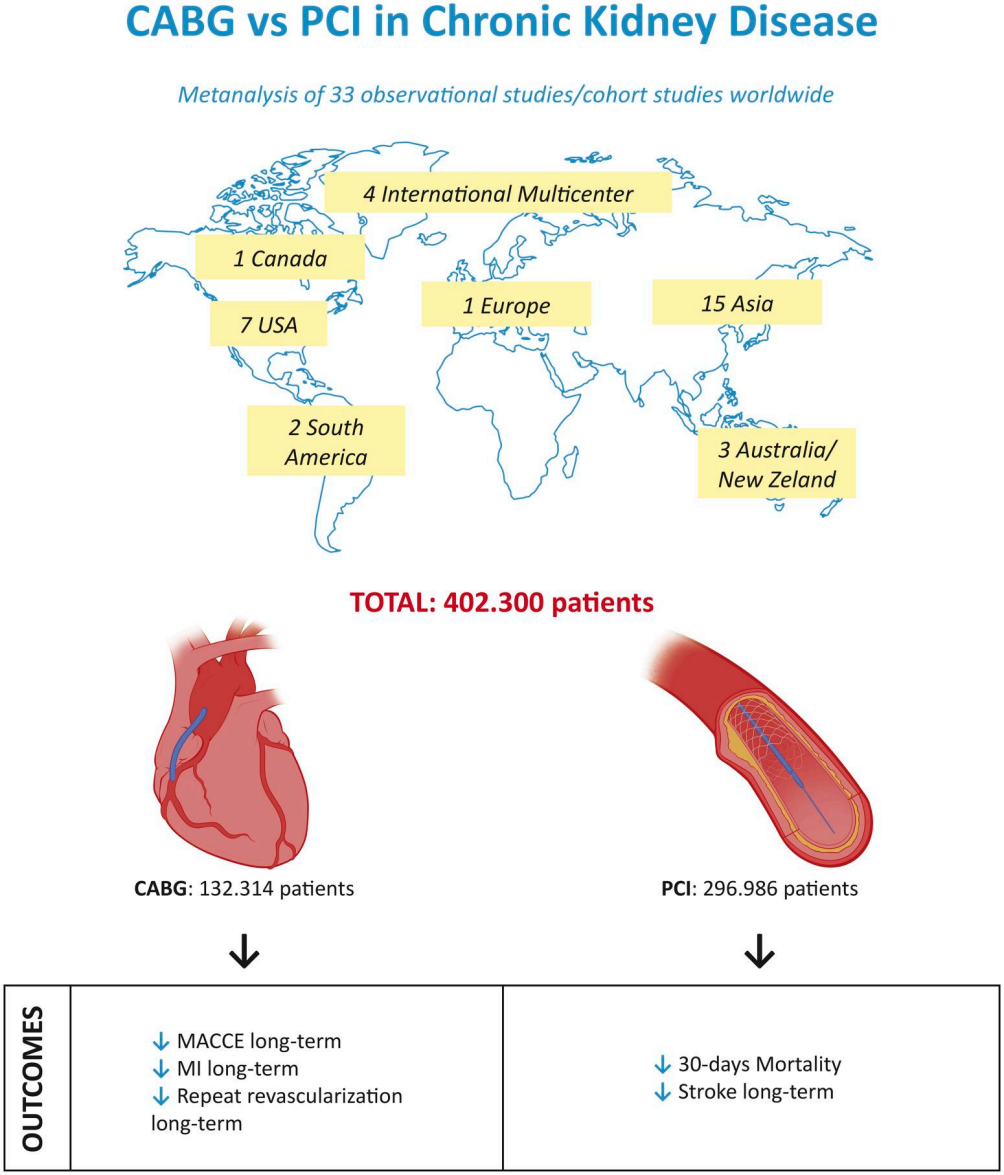
Central summary of results comparing CABG to PCI in CKD patients based on the meta-analysis

Figure 7 summarizes the results from this meta-analysis comparing CABG and PCI in CKD patients.

Multivariable meta-regression identified hypertension, diabetes, dialysis, publication year, and study type as sources of heterogeneity (Supplementary Table S2 and Bubble plots for univariable meta-regression in the Supplementary Material). Subgroup analyses revealed consistent findings across different characteristics (Supplementary Subgroup Forest Plot).

### Quality and risk of bias assessment

All studies encompassed within our systematic review and meta-analysis exhibited a low risk of bias regarding the representativeness of the general population and the selection of participants. Moreover, the majority demonstrated a low risk concerning confounding factors, missing data, outcome assessment, and follow-up.

Detailed evidence quality summaries for each study are provided in Supplementary Table S3.

## DISCUSSION

The key findings of this meta-analysis, comparing outcomes related to myocardial revascularization strategies (CABG vs PCI) in patients with moderate and severe CKD with an eGFR below 60 ml/min/1.73 m^2^, are as follows:

1. over a long-term period of 3-years, CABG demonstrates a protective effect against MACCE, MI, and the need for repeat revascularization;
2. in the short-term, specifically at the 30-days, PCI exhibits superior outcomes, particularly in terms of reduced early mortality;
3. during the 3-year follow-up, CABG reveals a higher incidence of stroke events compared to PCI.

Concerning the myocardial revascularization in CKD patients, our analysis found that early outcomes appear to favor PCI, however, in the long-term, CABG demonstrates superior results, which aligns with the data of previous studies [48, 49]. Notably, there was significant heterogeneity among the studies included in the meta-analysis. To address this, we conducted a meta-regression, considering variables that could potentially influence the outcomes. This additional analysis confirmed the initial findings, reinforcing the observed early advantage of PCI and the long-term benefit of CABG.

In case of coronary revascularization (CABG or PCI), CKD is recognized as a significant risk factor for the development of MACCE [31, 48–53]. Our analysis demonstrated the superiority of CABG over PCI in CKD patients for reducing MACCE in the long term, consistent with findings reported in the literature [1, 20, 28, 31, 34, 41, 43, 47, 50]. However, this conclusion is based on limited data, as only studies reporting this specific outcome were included, accounting for a total of 8632 patients out of an initial cohort of 402 300. This represents a significant limitation inherent to the available studies and the literature itself, rather than a methodological flaw of our analysis.

This superiority observed at medium-term follow-up appears to be primarily influenced by an increased rate of repeat revascularization in the PCI group: CKD patients undergoing PCI face a threefold higher risk of repeat revascularization at 5 years compared to CABG [1, 18, 54]. Despite DES improvements over the time, restenosis risk remains higher with PCI due to CKD’s unique atherosclerosis, characterized by calcified lesions and increased residual stenoses, that can lead to incomplete revascularization, increasing the risk of late MI and mortality [1, 19, 29, 35, 43, 48, 49, 51–60].

In contrast to PCI, the need for repeat revascularization is negligible with CABG due to the lower rates of graft dysfunction. This can be attributed to the unique properties of grafts, particularly arterial grafts, which release nitric oxide (NO) and other endothelial-protective factors. These biological advantages stand in stark contrast to the passive or potentially adverse effects of stents, including drug-eluting stents [61, 62].

One of the other major advantages of CABG is its protective effect against the progression of atherosclerotic lesions. By bypassing lengthy obstructive segments, CABG minimizes the impact of the atherosclerotic process on upstream proximal vessels [20, 63].

This, combined with the reduced risk of repeat revascularization, further supports our key finding that CABG significantly reduces the risk of late MI, a result also consistently reported in the literature [63, 64].

Concerning early outcomes, it is well recognized that factors such as fluid shifts during cardiopulmonary bypass (CPB), CKD-related anemia, platelet dysfunction, and the underlying atherosclerotic condition—often associated with severe peripheral vascular disease—may contribute to the heightened early mortality rate observed in the CABG group [54]. The higher 30-day mortality in the CKD-CABG cohort is largely attributed to the invasiveness of the procedure, which leads to increased postoperative complications such as bleeding, higher transfusion requirements, prolonged ventilation, and a greater risk of infections [44, 60, 65]. The avoidance of CPB through off-pump CABG may be an effective strategy to reduce postoperative renal damage, and worse outcomes, associated with on-pump procedures [66, 67].

In addition to the 30-day mortality rate, our analysis also demonstrated a higher long-term risk of stroke in the CABG group [19, 51, 59, 63, 68]. For instance, data from previous studies, such as the SYNTAX trial, highlight a higher incidence of stroke at 12 months in CABG patients compared to those undergoing PCI, potentially linked to the use of dual antiplatelet therapy (DAPT) following PCI [69, 70]. It is well known that contemporary stents often require shorter durations of DAPT (e.g. 6 months), which represents a limitation in the data, as our findings align with earlier studies but may not fully reflect current PCI practices [71–74]. With further studies incorporating more recent practices, it is plausible that the higher stroke rate observed in the CABG group may become equivalent to that in the PCI group.

In 2024, the debate on revascularization strategies in CKD remains critical. Although studies support CABG, and ESC guidelines recommend it for CKD patients, everyday clinical practice uncertainties persist regarding the optimal revascularization approach [11, 26]. The risk paradox often leads to less invasive treatment for higher-risk patients [28]. Surgeons worry about complex lesions, poor target vessels, stroke risk and worsening kidney function from CPB [26]. Cardiologists are concerned about contrast dosage impacting kidney function and lesions that may be untreatable with stents or prone to restenosis or thrombosis [26]. Effectively, CKD disrupts conventional cardiovascular paradigms: patients often presenting atypical symptoms and complex anatomical profiles, including multivessel involvement, calcification, and peripheral vascular disease [75]. This increases their risk of CAD, cerebrovascular events, and major adverse limb events, such as amputation [76, 77]. In CKD patients, particularly in hemodialysis, mesenteric ischemia has an annual incidence of 0.3–1.9%; non-occlusive forms manifest as splanchnic hypoperfusion and ischemia-reperfusion injury, with chronic bowel ischemia in chronic dialysis patients, frequently linked to atherosclerosis [76, 77]. The CKD-CAD interaction exacerbates the risk profile, with a synergistic effect rather than mere coexistence [36]. For this high-risk profile patients, many RCTs exclude CKD patients from the study [75].

Therefore, our only remaining course of action is to re-examine the existing literature through meta-analyses and to strive towards initiating new RCT specifically targeting this fragile, delicate, and complex patient population.

### Limitation

This meta-analysis has several limitations.

First, most included studies are observational or propensity-matched, increasing the risk of selection and treatment biases. Notably, there is a scarcity of RCTs focused specifically on CKD patients; instead, data come from more generalized RCTs comparing CABG to PCI.

Second, variations in the definition of MACCE across studies may introduce inconsistencies in the reported outcomes. Additionally, this outcome is available for a limited number of patients compared to the total cohort.

Third, there were notable differences in myocardial procedures and stent types used across studies, adding complexity to the analysis since stent characteristics can influence outcomes. Significant heterogeneity was observed in stroke, AMI and repeat revascularization results, likely due to varying clinical characteristics, intervention types, and follow-up periods. Additionally, most studies did not specify whether single or multiple arterial grafts were used in CABG. Subgroup analyses were conducted to address heterogeneity, which reduced but did not eliminate it, consistent with the literature [68, 78].

MACCE-free survival data faced issues with event timing, patient withdrawal, and follow-up inconsistencies. To standardize survival data, the Guyot algorithm was used to reconstruct individual patient data and compare HRs from original and reconstructed data.

## CONCLUSION

CABG is more effective than PCI for CAD in CKD patients, leading to a greater reduction in MACCE events, particularly in repeat revascularization and new MIs due to restenosis.

However, CABG was associated with higher 30-day mortality and an increased incidence of long-term strokes.

This meta-analysis highlights the need for more robust RCTs on myocardial revascularization in CKD patients with CAD, essential for refining clinical guidelines and improving outcomes in this complex group.

## Supplementary Material

ivaf021_Supplementary_Data

## Data Availability

The data underlying this article will be shared on reasonable request to the corresponding author.

## ABBREVIATIONS

BMS: Bare-metal stent
CABG: Coronary artery bypass graft
CAD: Coronary artery disease
CKD: Chronic kidney disease
CVD: Cardiovascular disease
DES: Drug eluting stent
EACTS: European Association for Cardio-Thoracic Surgery
EES: Everolimus-eluting stent
ESC: European Society of Cardiology
GFR: Glomerular filtration rate
MACCE: Major adverse cardiovascular and cerebrovascular events
MI: Myocardial infarction
PCI: Percutaneous coronary intervention
RCT: Randomized clinical trial
