# Supplementary material for: Myocardial revascularization in patients with chronic kidney disease: a systematic review and meta-analysis of surgical versus percutaneous coronary revascularization
Source: Interdiscip Cardiovasc Thorac Surg. 2025 Feb 19;40(3):ivaf021. doi: 10.1093/icvts/ivaf021 (PMC11897794; doi:10.1093/icvts/ivaf021)
Supplement: ivaf021_Supplementary_Data [file ivaf021_supplementary_data.zip › Supplemental Material_VG_13 01 2025.docx]

**Supplemental Material**

Table S1. PRISMA checklist

| **Section and Topic** | **Item #** | **Checklist item** | **Location where item is reported** |
| --- | --- | --- | --- |
| **TITLE** | | |  |
| Title | 1 | Identify the report as a systematic review. | Title page (page 1) |
| **ABSTRACT** | | |  |
| Abstract | 2 | See the PRISMA 2020 for Abstracts checklist. | Page 3 |
| **INTRODUCTION** | | |  |
| Rationale | 3 | Describe the rationale for the review in the context of existing knowledge. | Page 5 |
| Objectives | 4 | Provide an explicit statement of the objective(s) or question(s) the review addresses. | Page 5 |
| **METHODS** | | |  |
| Eligibility criteria | 5 | Specify the inclusion and exclusion criteria for the review and how studies were grouped for the syntheses. | Page 6 |
| Information sources | 6 | Specify all databases, registers, websites, organisations, reference lists and other sources searched or consulted to identify studies. Specify the date when each source was last searched or consulted. | Page 6 |
| Search strategy | 7 | Present the full search strategies for all databases, registers and websites, including any filters and limits used. | Page 6 |
| Selection process | 8 | Specify the methods used to decide whether a study met the inclusion criteria of the review, including how many reviewers screened each record and each report retrieved, whether they worked independently, and if applicable, details of automation tools used in the process. | Page 6-7 |
| Data collection process | 9 | Specify the methods used to collect data from reports, including how many reviewers collected data from each report, whether they worked independently, any processes for obtaining or confirming data from study investigators, and if applicable, details of automation tools used in the process. | Page 6-7 |
| Data items | 10a | List and define all outcomes for which data were sought. Specify whether all results that were compatible with each outcome domain in each study were sought (e.g. for all measures, time points, analyses), and if not, the methods used to decide which results to collect. | Page 7 |
|  | 10b | List and define all other variables for which data were sought (e.g. participant and intervention characteristics, funding sources). Describe any assumptions made about any missing or unclear information. | Page 7 |
| Study risk of bias assessment | 11 | Specify the methods used to assess risk of bias in the included studies, including details of the tool(s) used, how many reviewers assessed each study and whether they worked independently, and if applicable, details of automation tools used in the process. | Page 8 |
| Effect measures | 12 | Specify for each outcome the effect measure(s) (e.g. risk ratio, mean difference) used in the synthesis or presentation of results. | Page 8 |
| Synthesis methods | 13a | Describe the processes used to decide which studies were eligible for each synthesis (e.g. tabulating the study intervention characteristics and comparing against the planned groups for each synthesis (item #5)). | Page 7-8 |
|  | 13b | Describe any methods required to prepare the data for presentation or synthesis, such as handling of missing summary statistics, or data conversions. | Page 7-8 |
|  | 13c | Describe any methods used to tabulate or visually display results of individual studies and syntheses. | Page 7-8 |
|  | 13d | Describe any methods used to synthesize results and provide a rationale for the choice(s). If meta-analysis was performed, describe the model(s), method(s) to identify the presence and extent of statistical heterogeneity, and software package(s) used. | Page 7-8 |
|  | 13e | Describe any methods used to explore possible causes of heterogeneity among study results (e.g. subgroup analysis, meta-regression). | Page 7-8 |
|  | 13f | Describe any sensitivity analyses conducted to assess robustness of the synthesized results. | Page 7-8 |
| Reporting bias assessment | 14 | Describe any methods used to assess risk of bias due to missing results in a synthesis (arising from reporting biases). | Page 7-8 |
| Certainty assessment | 15 | Describe any methods used to assess certainty (or confidence) in the body of evidence for an outcome. | Page 7-8 |
| **RESULTS** | | |  |
| Study selection | 16a | Describe the results of the search and selection process, from the number of records identified in the search to the number of studies included in the review, ideally using a flow diagram. | Page 9, Figure 1 |
|  | 16b | Cite studies that might appear to meet the inclusion criteria, but which were excluded, and explain why they were excluded. | Page 9 |
| Study characteristics | 17 | Cite each included study and present its characteristics. | Table 1 |
| Risk of bias in studies | 18 | Present assessments of risk of bias for each included study. | Supplementary table |
| Results of individual studies | 19 | For all outcomes, present, for each study: (a) summary statistics for each group (where appropriate) and (b) an effect estimate and its precision (e.g. confidence/credible interval), ideally using structured tables or plots. | Page 9-10 |
| Results of syntheses | 20a | For each synthesis, briefly summarise the characteristics and risk of bias among contributing studies. | Page 11 |
|  | 20b | Present results of all statistical syntheses conducted. If meta-analysis was done, present for each the summary estimate and its precision (e.g. confidence/credible interval) and measures of statistical heterogeneity. If comparing groups, describe the direction of the effect. | Page 10 |
|  | 20c | Present results of all investigations of possible causes of heterogeneity among study results. | Page 10 |
|  | 20d | Present results of all sensitivity analyses conducted to assess the robustness of the synthesized results. | Page 10 |
| Reporting biases | 21 | Present assessments of risk of bias due to missing results (arising from reporting biases) for each synthesis assessed. | Page 11 |
| Certainty of evidence | 22 | Present assessments of certainty (or confidence) in the body of evidence for each outcome assessed. | Page 9-10 |
| **DISCUSSION** | | |  |
| Discussion | 23a | Provide a general interpretation of the results in the context of other evidence. | Page 12 |
|  | 23b | Discuss any limitations of the evidence included in the review. | Page 12 and 16 |
|  | 23c | Discuss any limitations of the review processes used. | Page 12 and 16 |
|  | 23d | Discuss implications of the results for practice, policy, and future research. | Page 14-15 |
| **OTHER INFORMATION** | | |  |
| Registration and protocol | 24a | Provide registration information for the review, including register name and registration number, or state that the review was not registered. | Page 6 |
|  | 24b | Indicate where the review protocol can be accessed, or state that a protocol was not prepared. | Page 6 |
|  | 24c | Describe and explain any amendments to information provided at registration or in the protocol. | Page 6 |
| Support | 25 | Describe sources of financial or non-financial support for the review, and the role of the funders or sponsors in the review. | Page 19 |
| Competing interests | 26 | Declare any competing interests of review authors. | Page 20 |
| Availability of data, code and other materials | 27 | Report which of the following are publicly available and where they can be found: template data collection forms; data extracted from included studies; data used for all analyses; analytic code; any other materials used in the review. | Page 6 |

Figure S1. Sensitivity analysis on MACCE-free survival


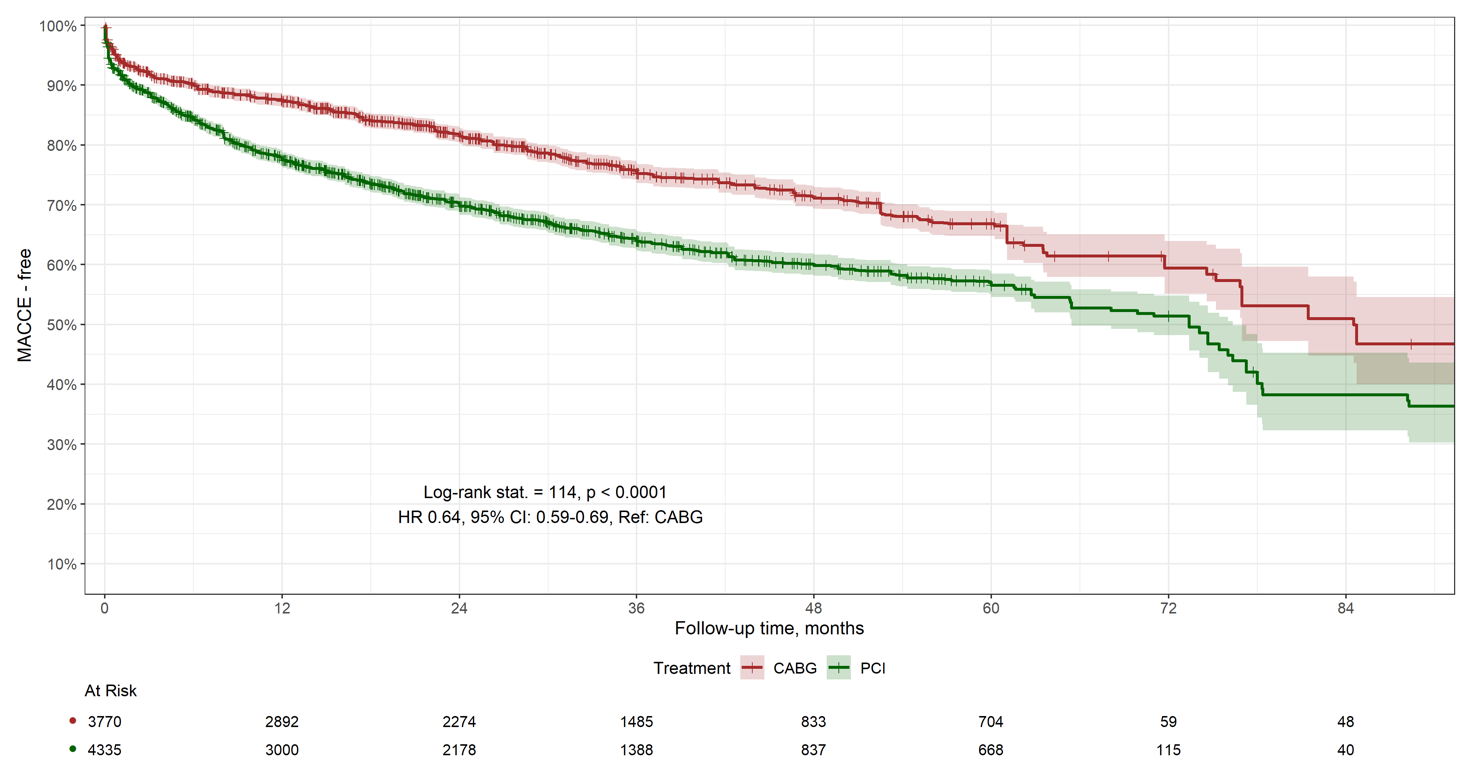


Figure S2. Funnel of small study effect for the early mortality


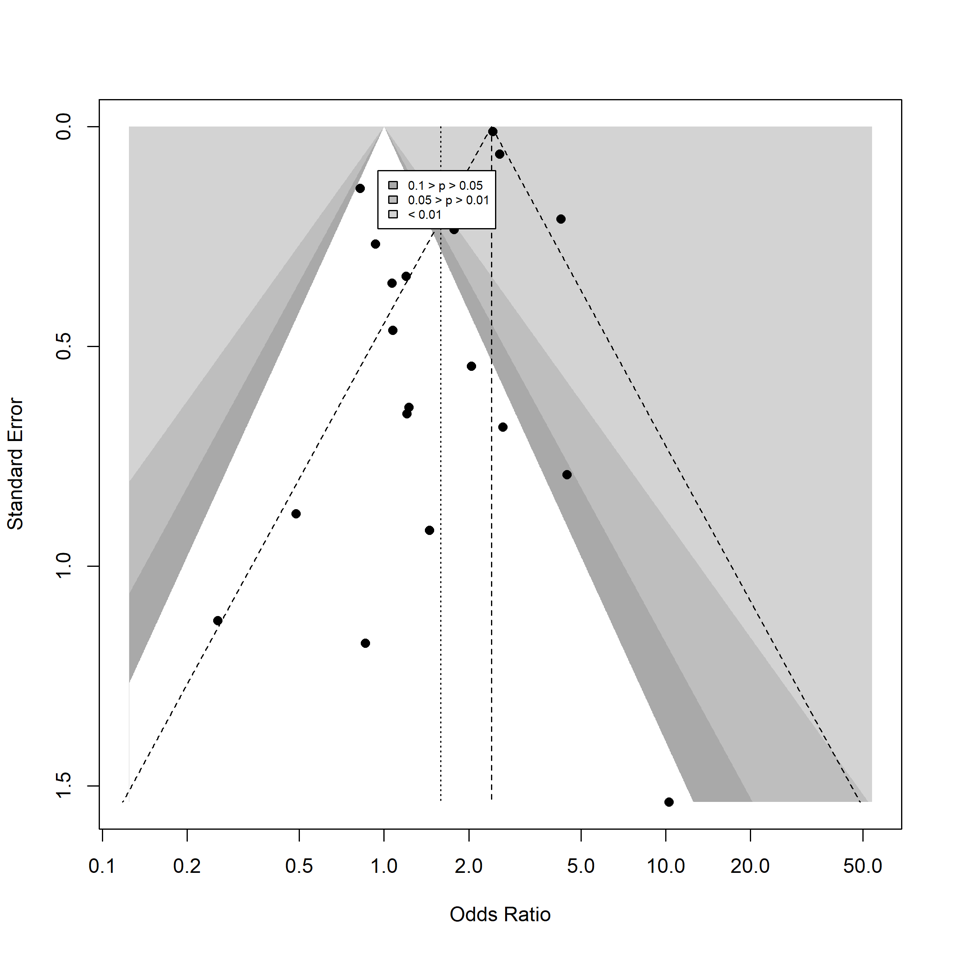


Figure S3. Leave-one-out analysis for the early mortality


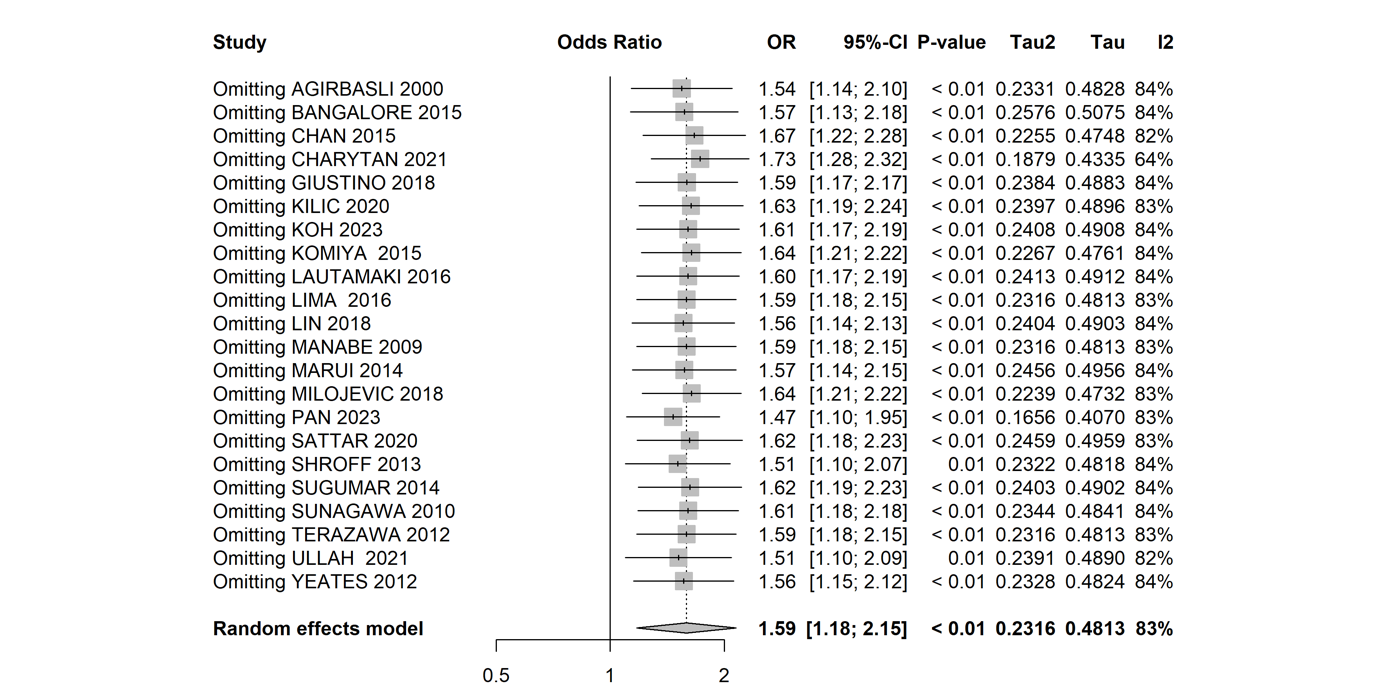


Figure S4. Funnel of small study effect for stroke


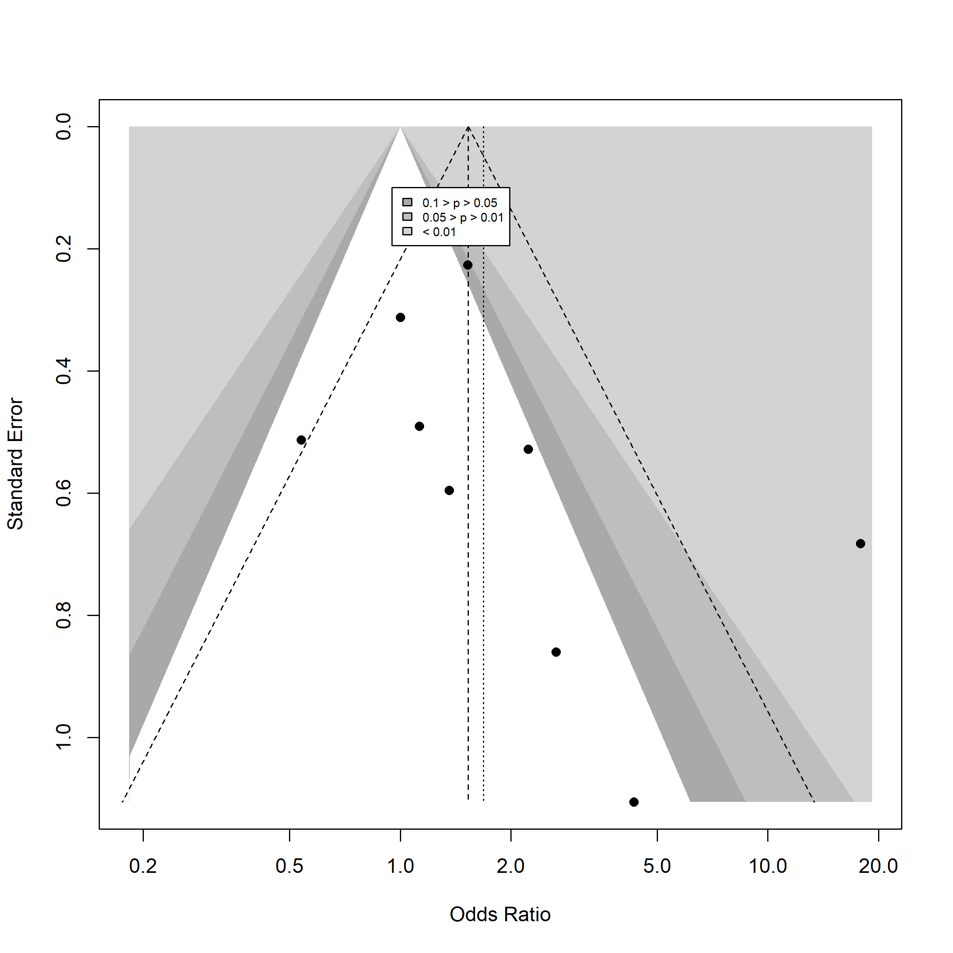


Figure S5. Leave-one-out analysis for stroke


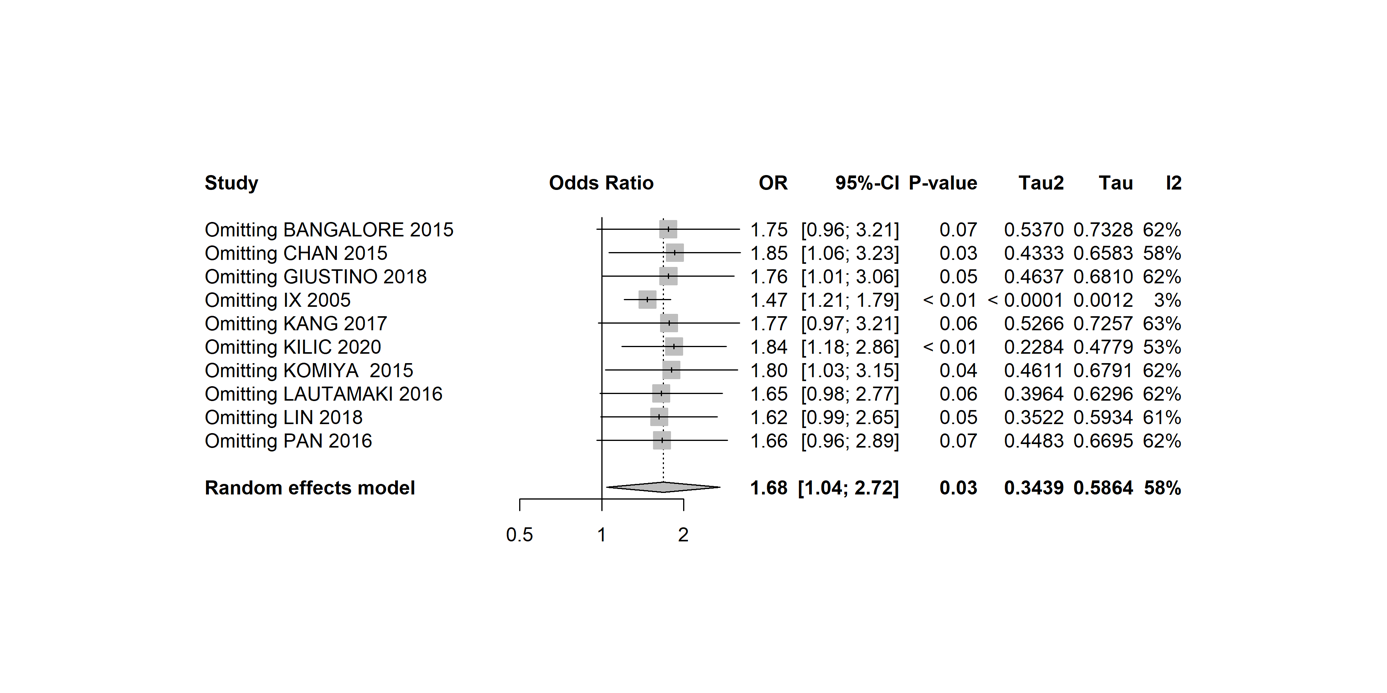


Figure S6. Funnel of small study effect for myocardial infarction


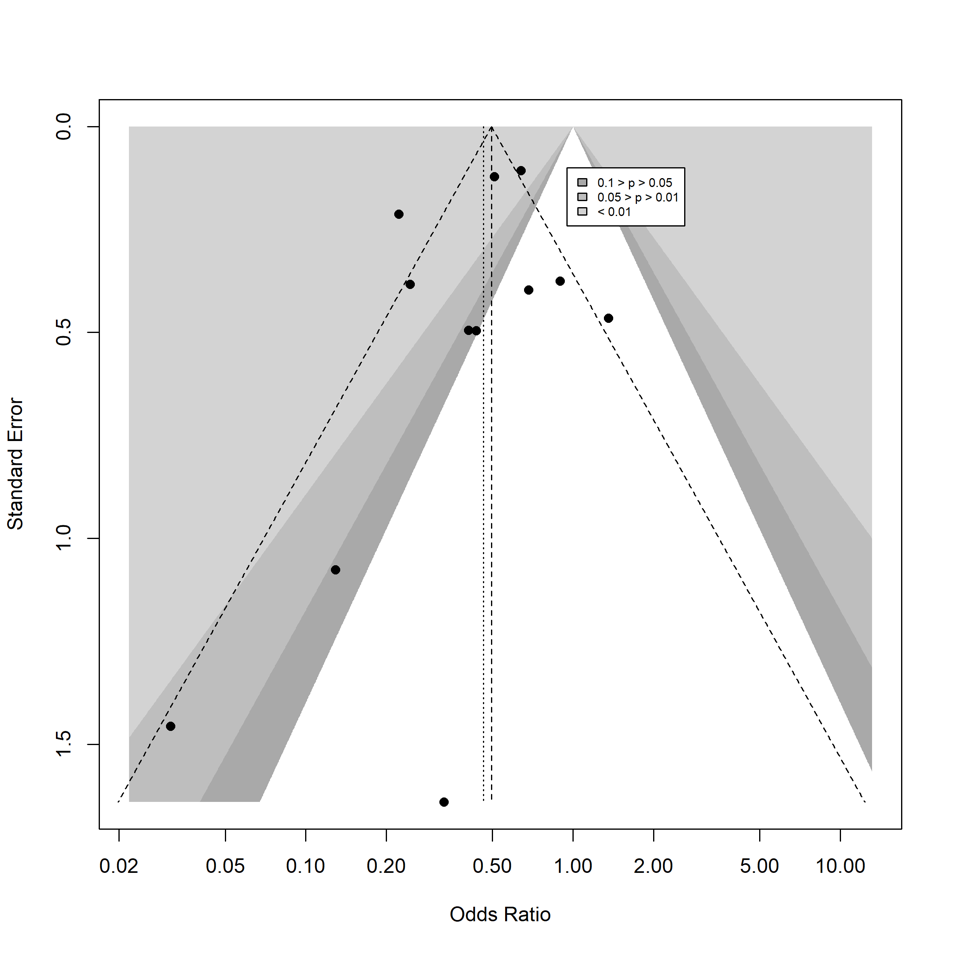


Figure S7. Leave-one-out analysis for myocardial infarction


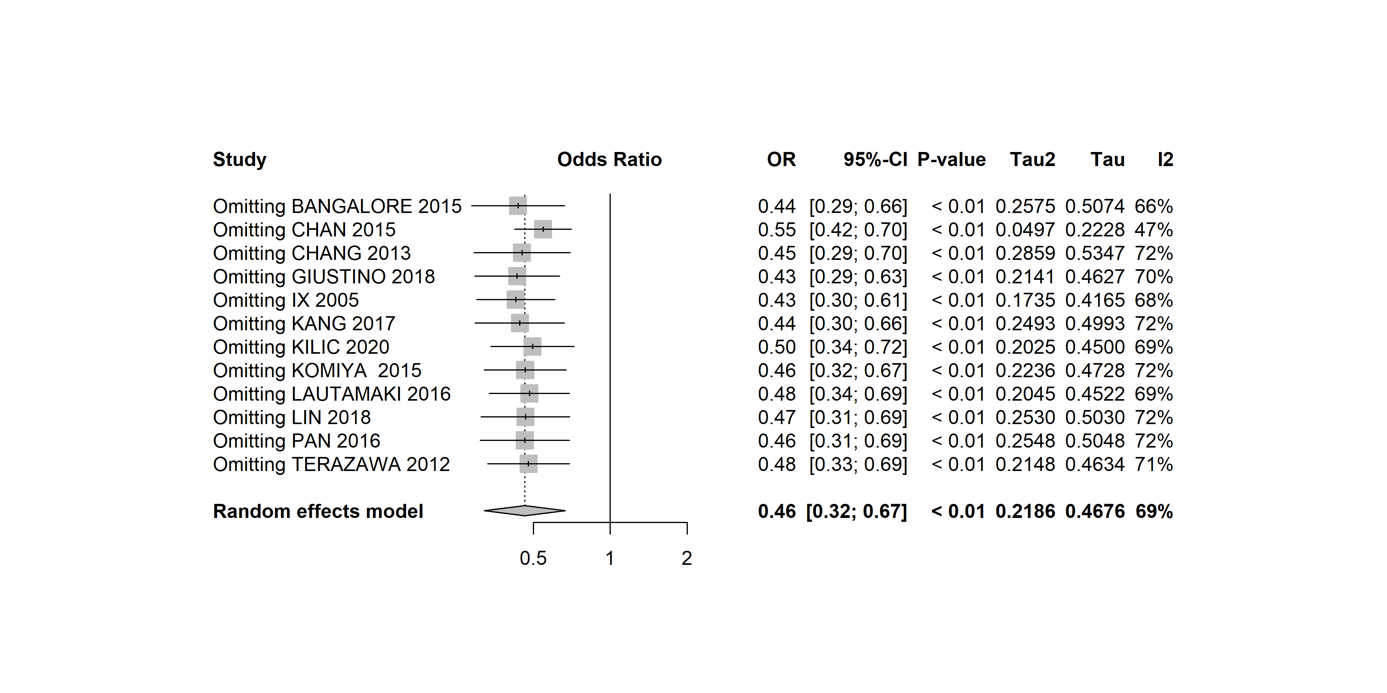


Figure S8. Funnel of small study effect for repeat revascularization


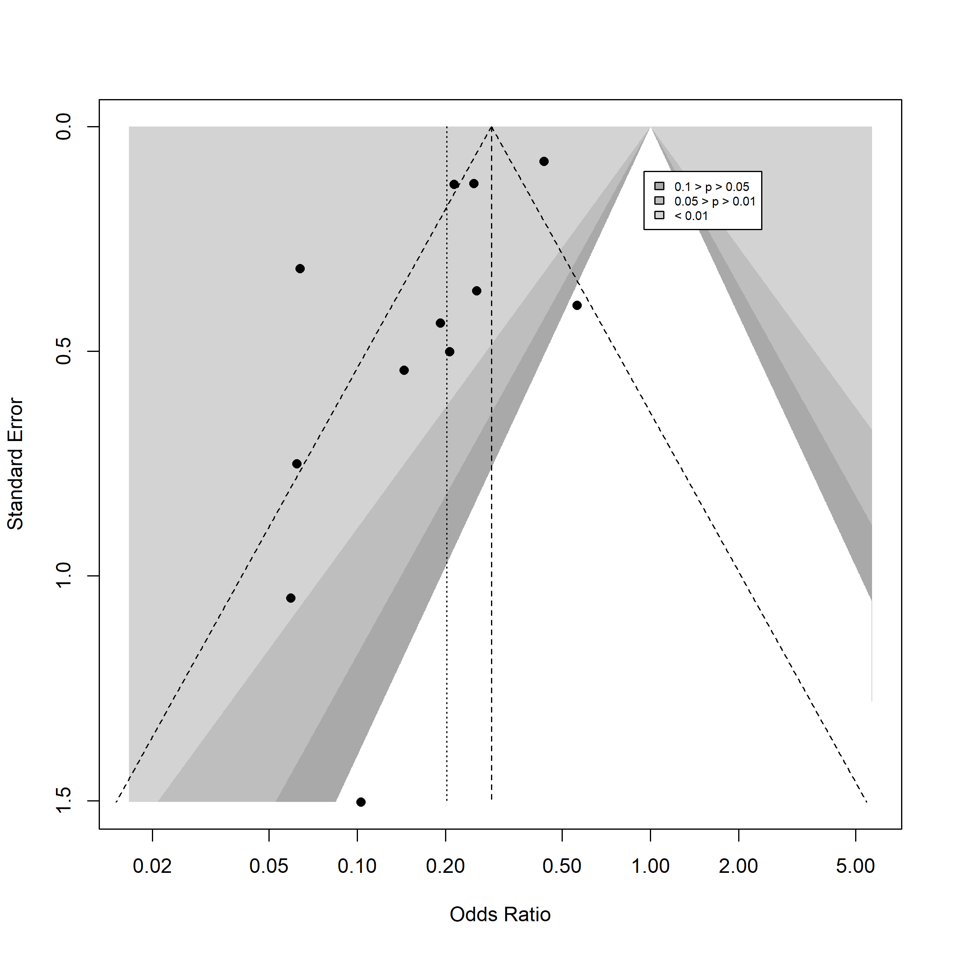


Figure S9. Leave-one-out analysis for repeat revascularization


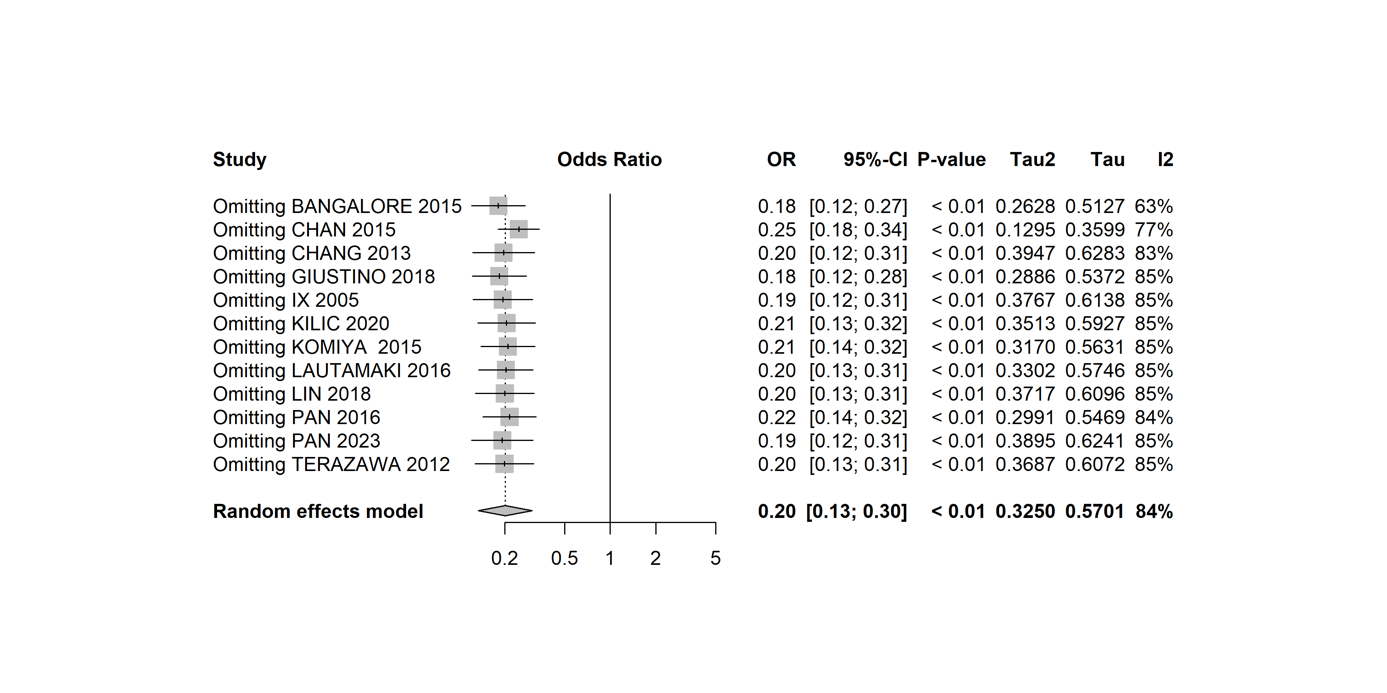


Table S2. Main Characteristics of eligible studies and patients’ demographics

| *30-days mortality* | | |
| --- | --- | --- |
| Moderator | **β (95% CI)** | **p Value** |
| *Clinical* | | |
| Hypertension | -0.001 (-0.003, 0.001) | 0.156 |
| Diabetes | 0.002 (-0.001, 0.006) | 0.154 |
| Renal replacement therapy | 0.001 (-0.0001, 0.001) | 0.018 |
| *Methodology* | | |
| Year of publication | 0.034 (-0.05, 0.128) | 0.447 |
| Study type: Cohort study | REF |  |
| Study type: Post-hoc RCT | -0.367 (-1.383, 0.648) | 0.452 |
| Study type: Propensity matched study | -0.505 (-1.208, 0.199) | 0.147 |
| *Stoke at 3 years of follow-up* | | |
| *Clinical* | | |
| Hypertension | 0.008 (-0.321, 0.336) | 0.817 |
| Diabetes | -0.025 (-0.954, 0.904) | 0.789 |
| Renal replacement therapy | 0.076 (-2.361, 2.514) | 0.760 |
| *Methodology* | | |
| Year of publication | -0.196 (-0.307, -0.086) | 0.005 |
| Study type: Cohort study | REF | |
| Study type: Post-hoc RCT | -0.368 (-0.890, 0.163) | 0.141 |
| Study type: Propensity matched study | -0.709 (-1.316, -0.103) | 0.029 |
| *MI at 3 years of follow-up* | | |
| *Clinical* | | |
| Hypertension | -0.002 (-0.015, 0.011) | 0.537 |
| Diabetes | 0.005 (-0.025, 0.034) | 0.565 |
| Renal replacement therapy | -0.005 (-0.068, 0.058) | 0.767 |
| Methodology | | |
| Year of publication | -0.024 (-0.098, 0.049) | 0.469 |
| Study type: Cohort study | REF | |
| Study type: Post-hoc RCT | 0.324 (-0.006, 0.655) | 0.053 |
| Study type: Propensity matched study | -0.757 (-1.253, -0.262) | 0.008 |
| Repeat revascularization *at 3 years of follow-up* | | |
| *Clinical* | | |
| Hypertension | -0.005 (-0.007, -0.003) | 0.003 |
| Diabetes | 0.009 (0.006, 0.012) | 0.003 |
| Renal replacement therapy | -0.001 (-0.0009, -0.0002) | 0.013 |
| Methodology | | |
| Year of publication | 0.083 (0.0131, 0.152) | 0.025 |
| Study type: Cohort study | REF | |
| Study type: Post-hoc RCT | 0.807 (0.192, 1.422) | 0.016 |
| Study type: Propensity matched study | -0.731 (-1.481, 0.019) | 0.054 |

Figure S10_A. Bubble plots for univariate meta-regression for 30-day mortality, covariate hypertension.


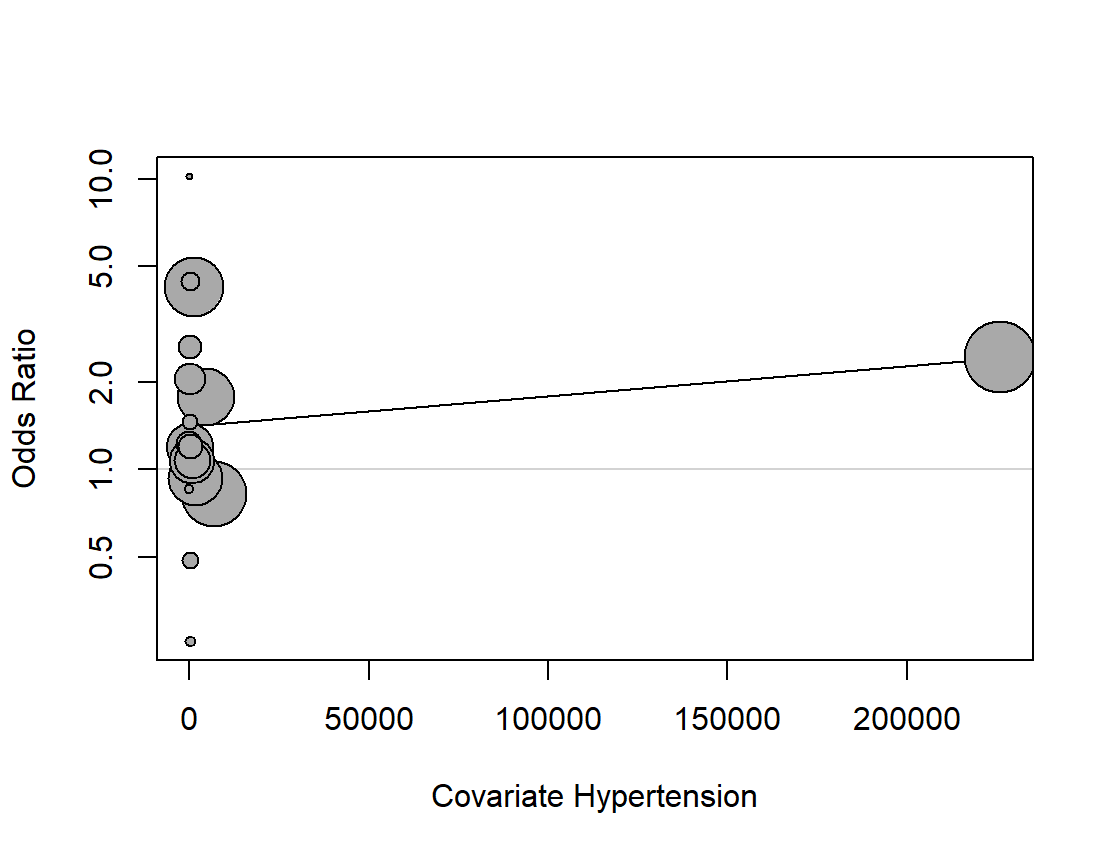


Figure S10_B. Bubble plots for univariate meta-regression for 30-day mortality, covariate diabetes.


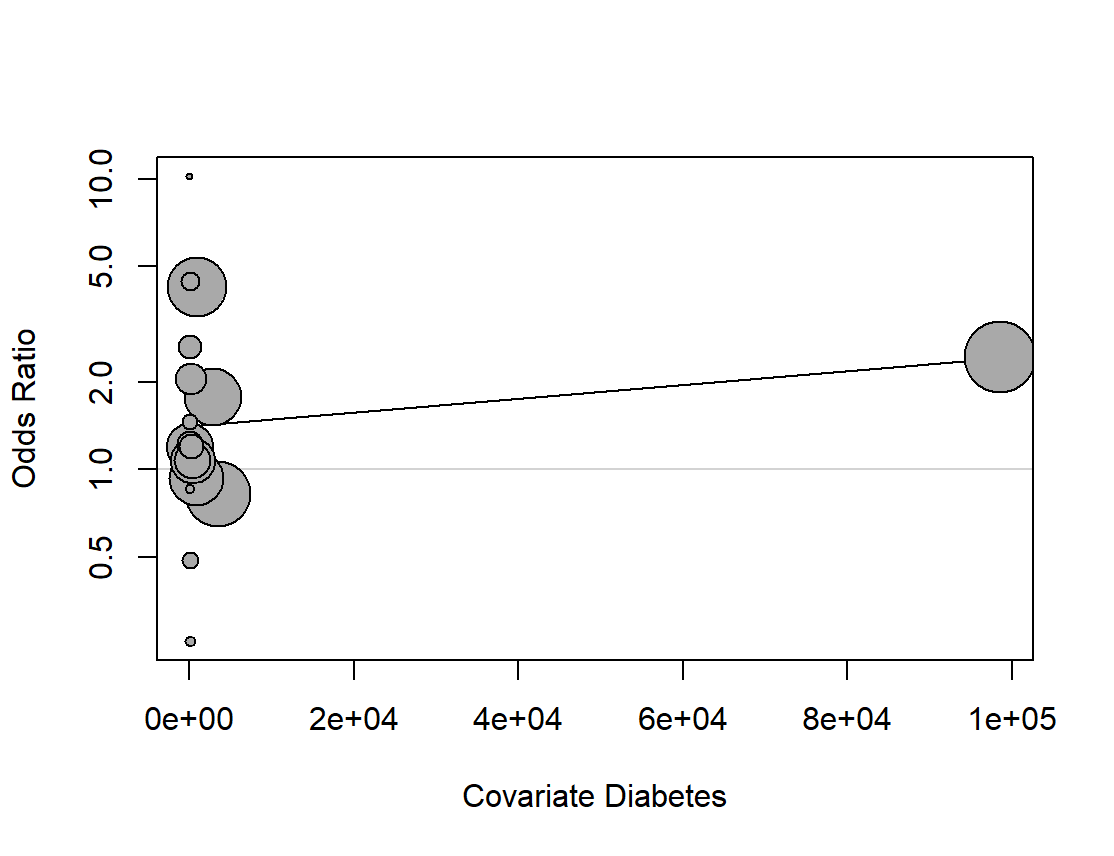


Figure S10_C. Bubble plots for univariate meta-regression for 30-day mortality, covariate dialysis.


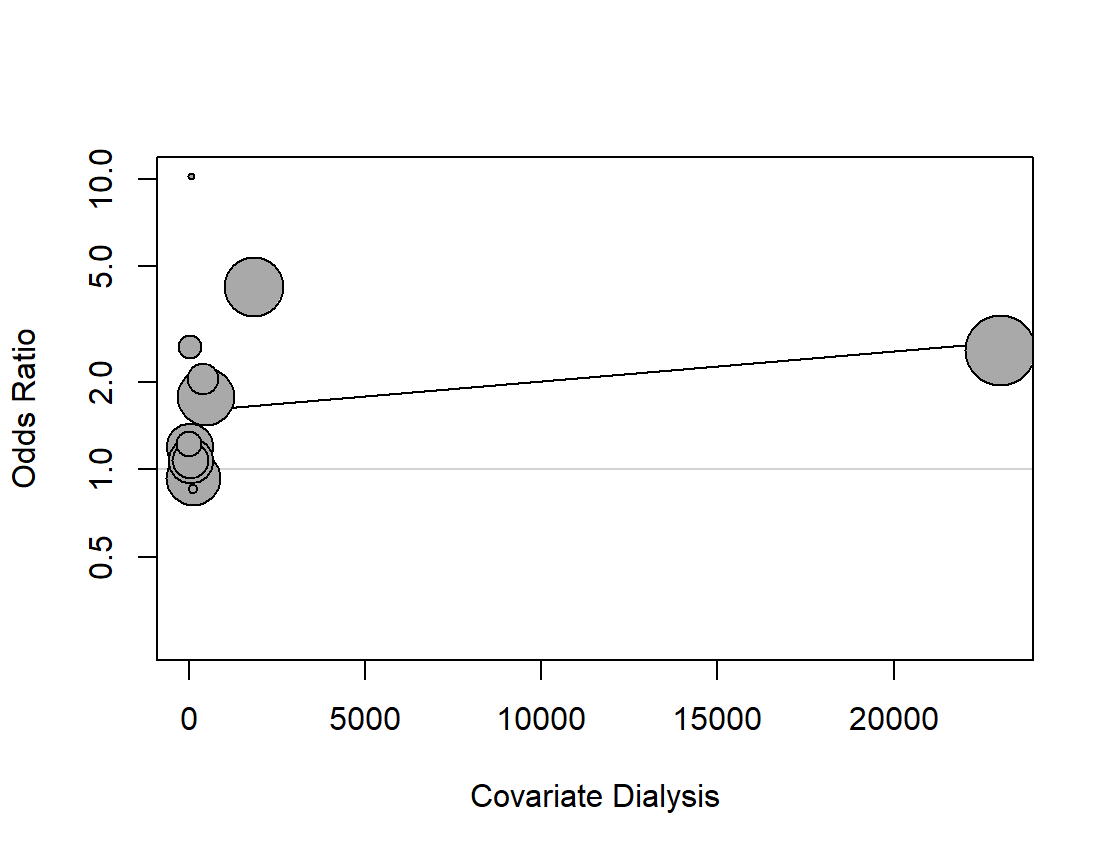


Figure S10_D. Bubble plots for univariate meta-regression for 30-day mortality, covariate study type.


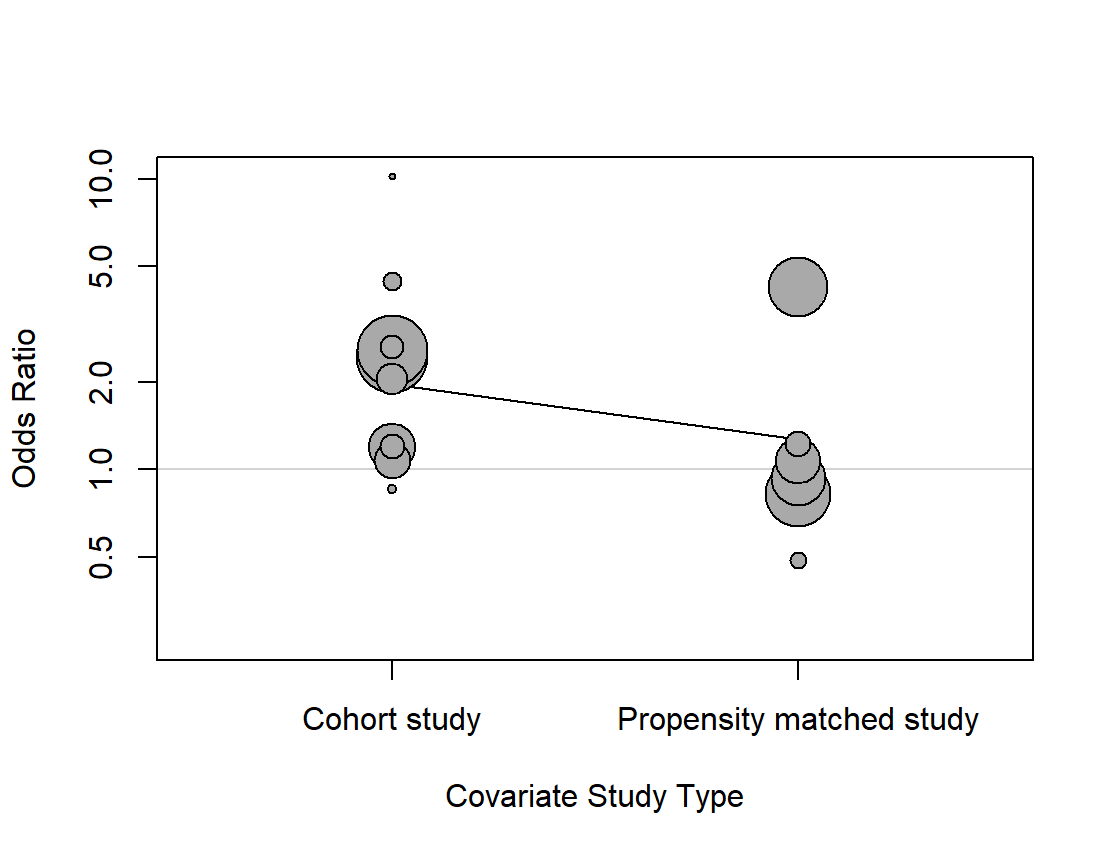


Figure S10_E. Bubble plots for univariate meta-regression for 30-day mortality, covariate publication year.


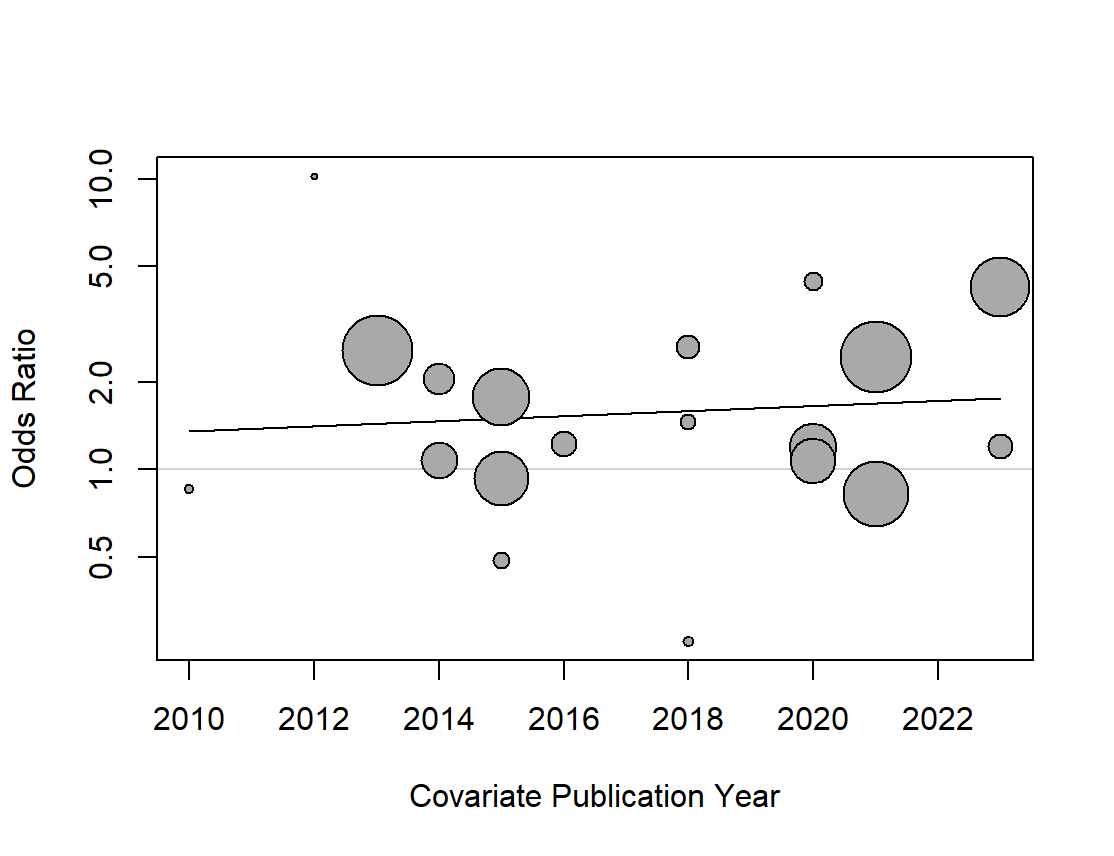


Figure S11_A. Bubble plots for univariate meta-regression for myocardial infarction at 3 years, covariate hypertension


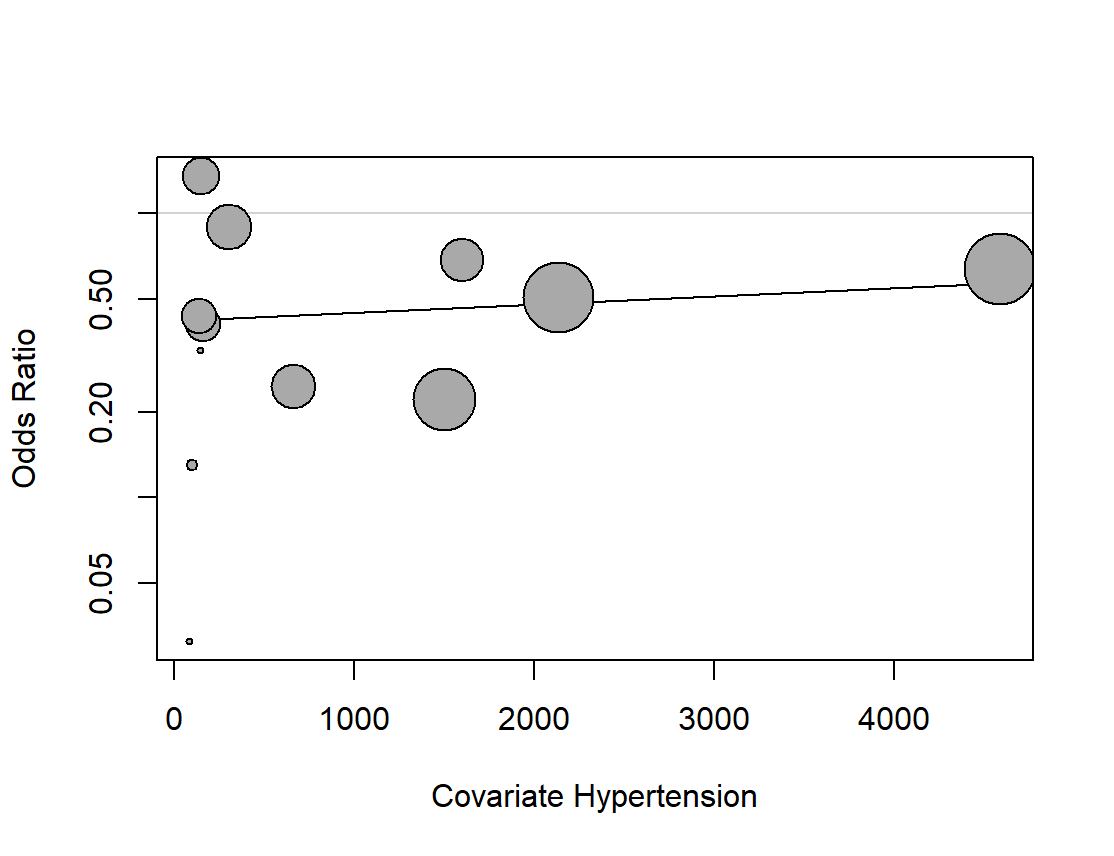


Figure S11_B. Bubble plots for univariate meta-regression for myocardial infarction at 3 years, covariate diabetes


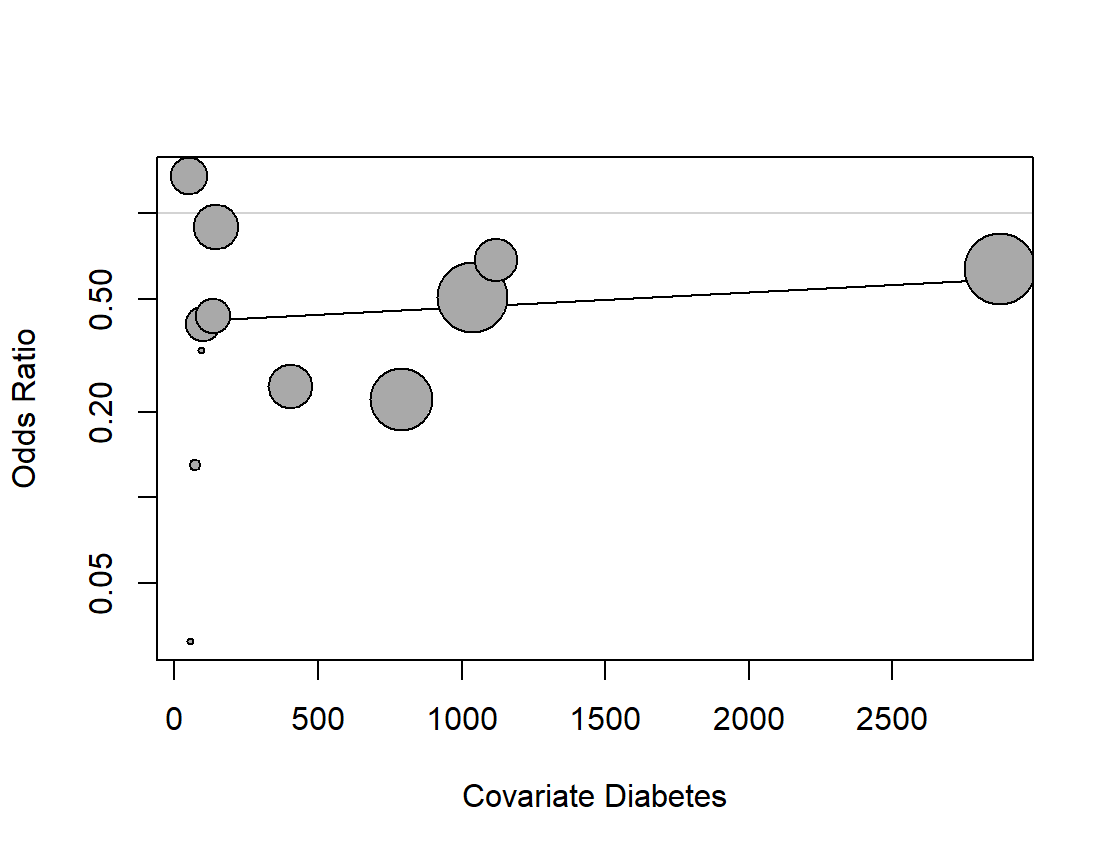


Figure S11_C. Bubble plots for univariate meta-regression for myocardial infarction at 3 years, covariate dialysis


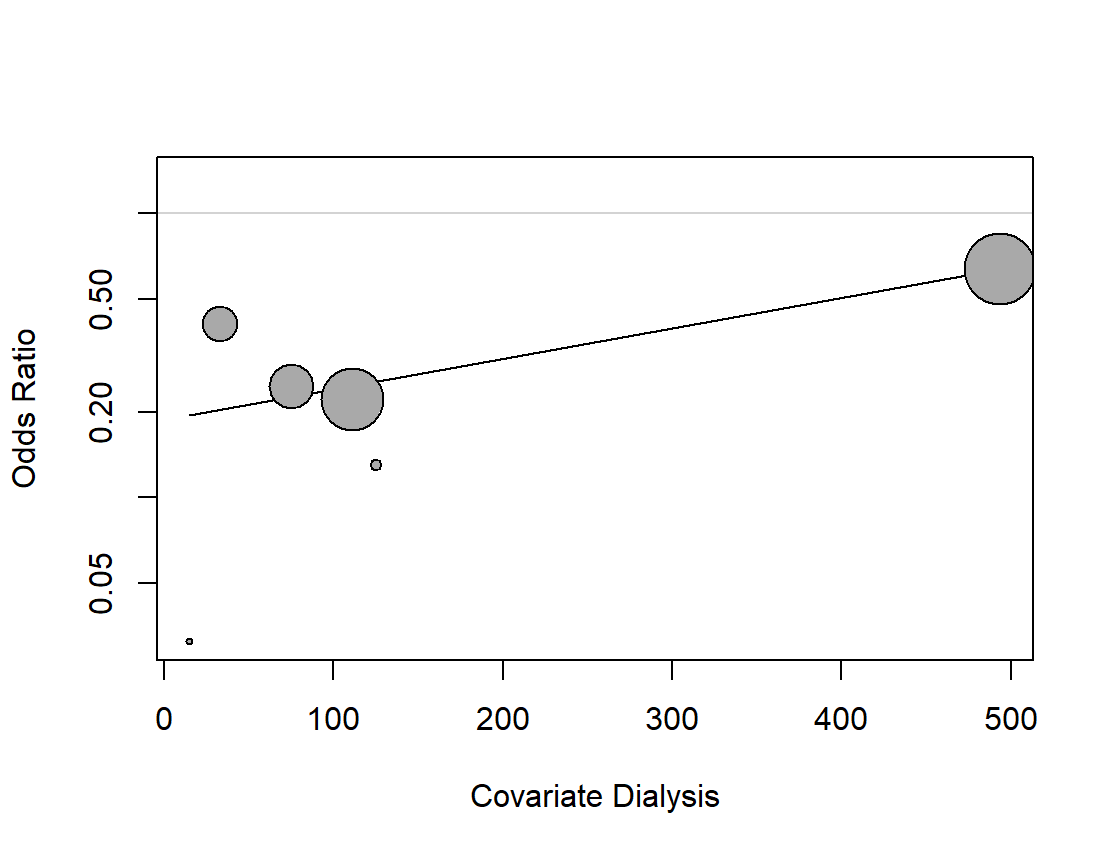


Figure S11_D. Bubble plots for univariate meta-regression for myocardial infarction at 3 years, covariate study type


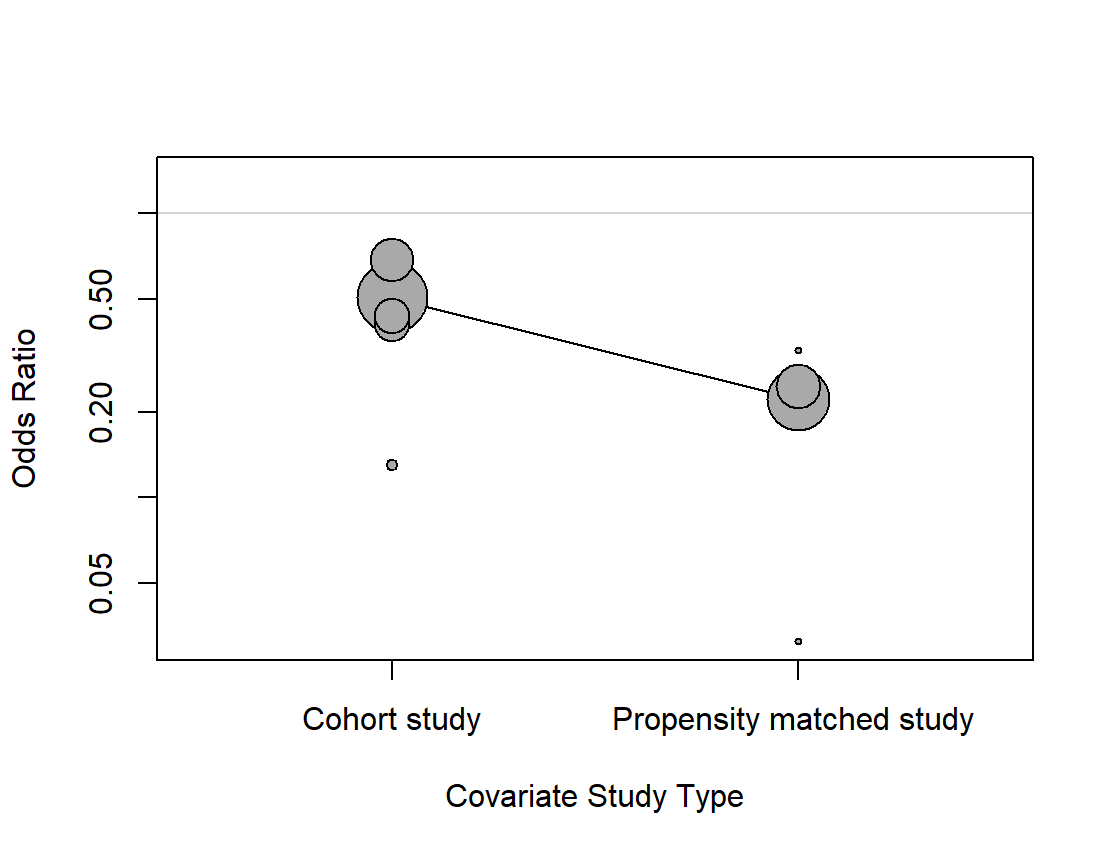


Figure S11_E. Bubble plots for univariate meta-regression for myocardial infarction at 3 years, covariate publication year


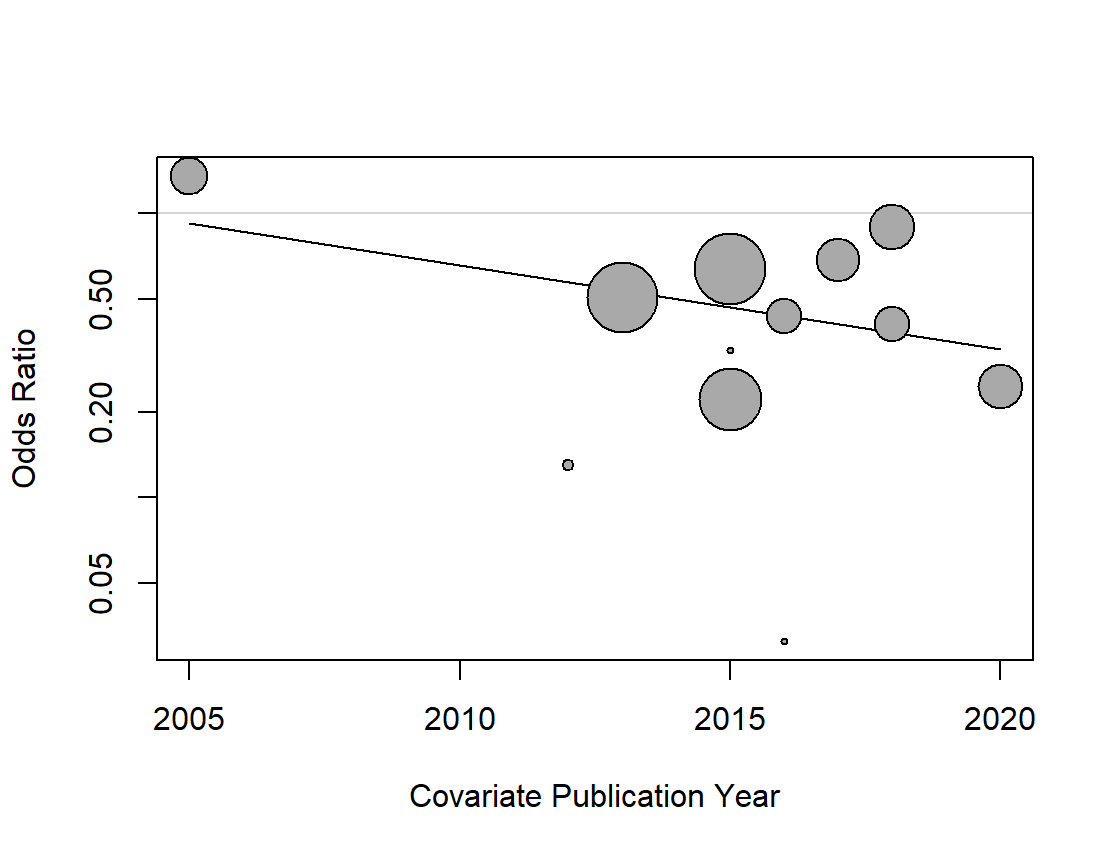


Figure S12_A. Bubble plots for univariate meta-regression for repeat revascularization at 3 years, covariate hypertension


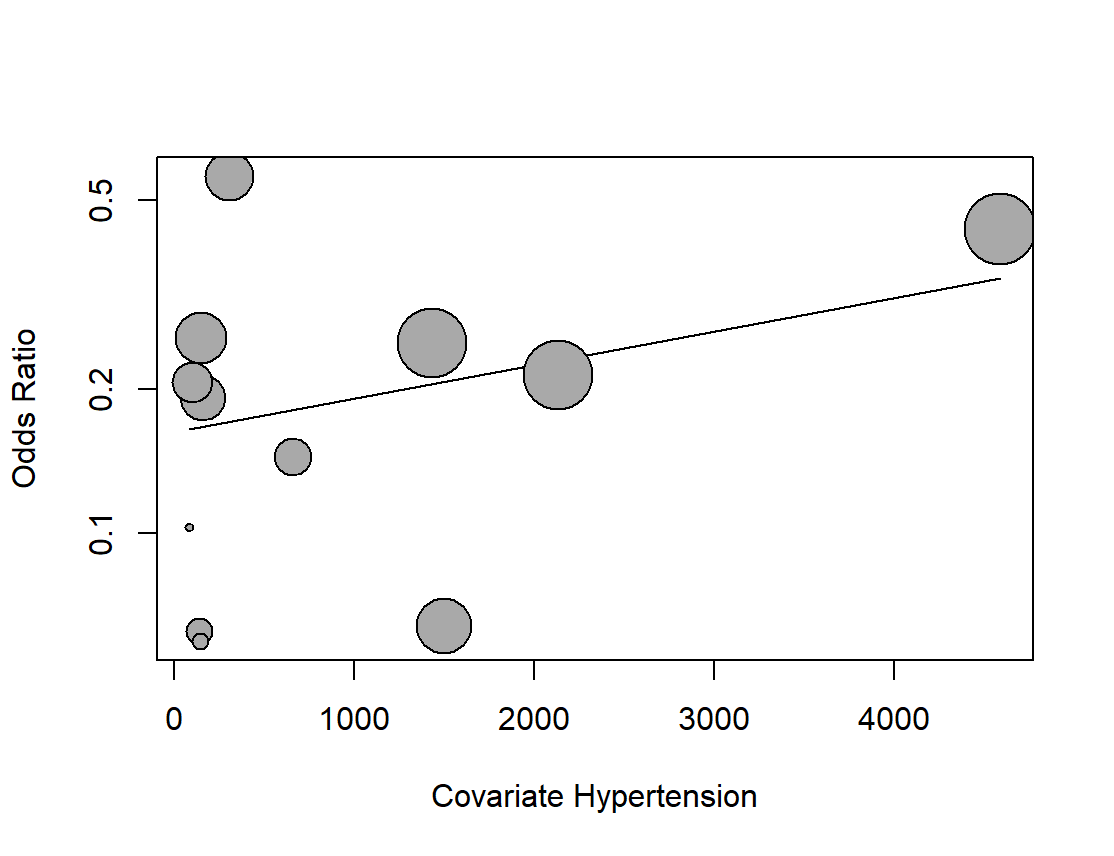


Figure S12_B. Bubble plots for univariate meta-regression for repeat revascularization at 3 years, covariate diabetes


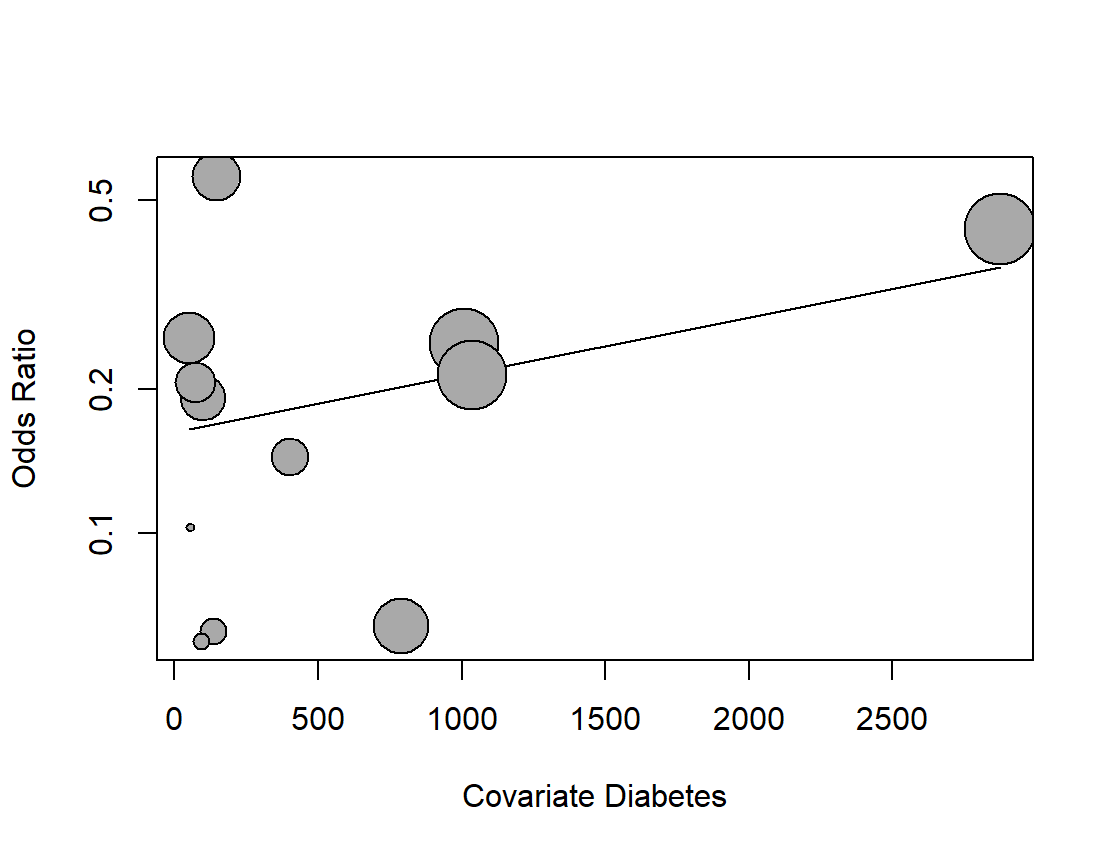


Figure S12_C. Bubble plots for univariate meta-regression for repeat revascularization at 3 years, covariate dialysis


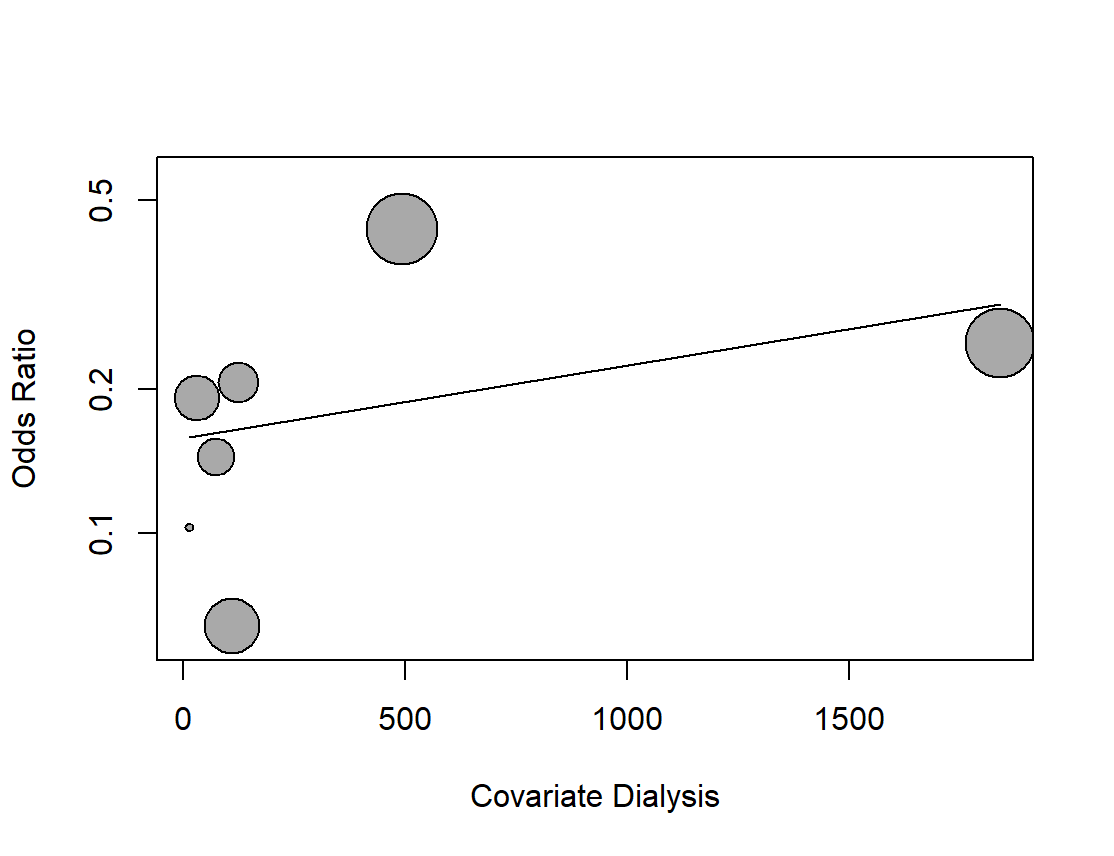


Figure S12_D. Bubble plots for univariate meta-regression for repeat revascularization at 3 years, covariate study type


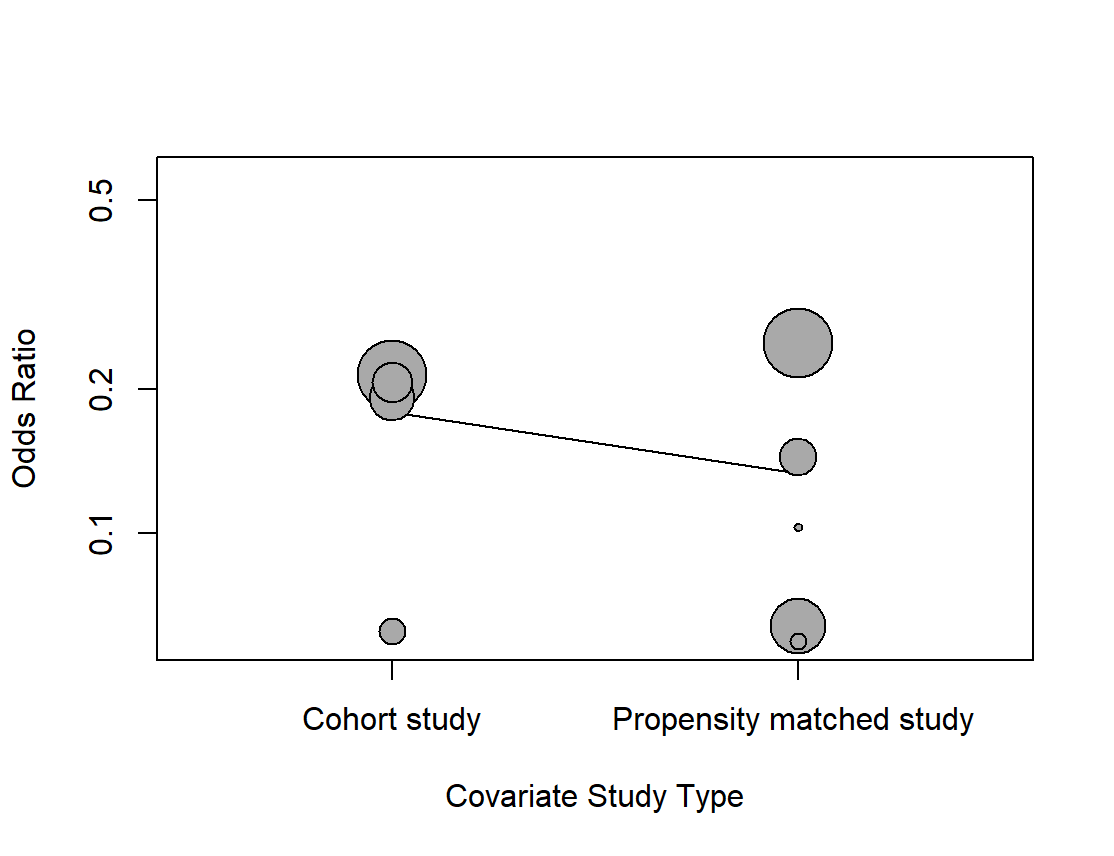


Figure S12_E. Bubble plots for univariate meta-regression for repeat revascularization at 3 years, covariate publication year


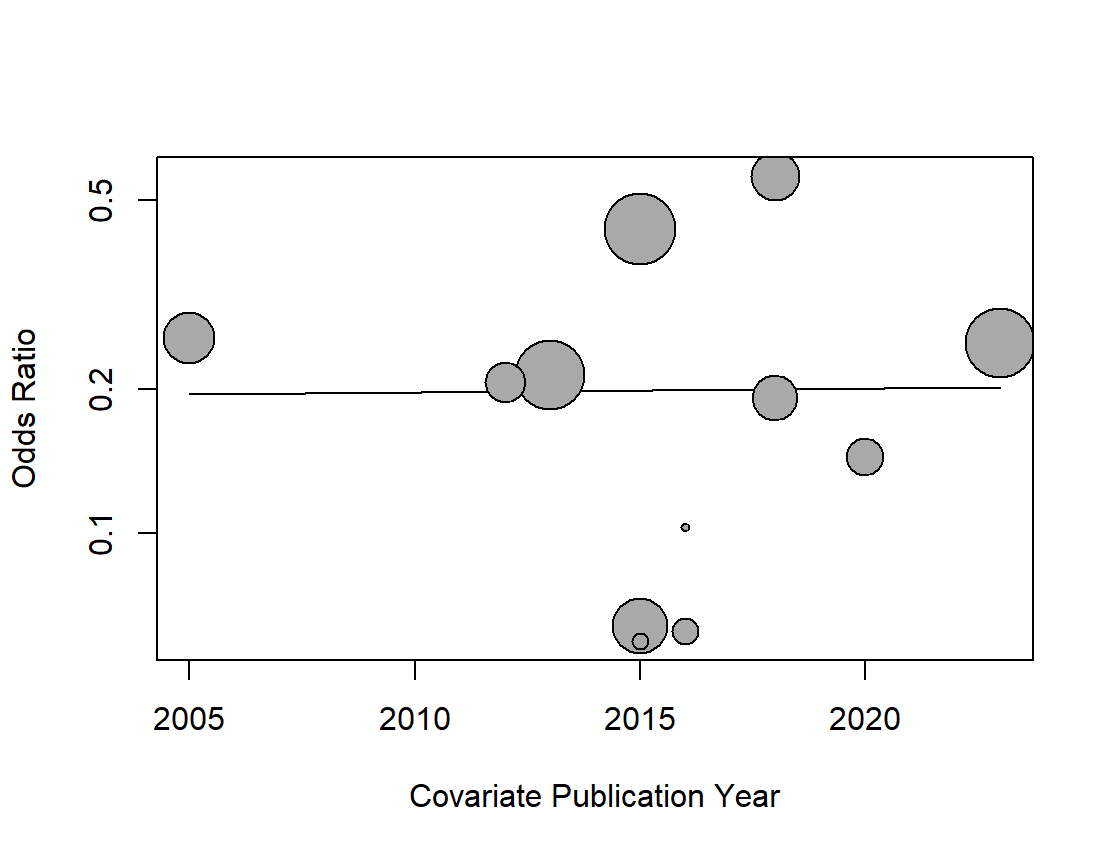


Figure S13_A. Bubble plots for univariate meta-regression for stroke at 3 years, covariate hypertension


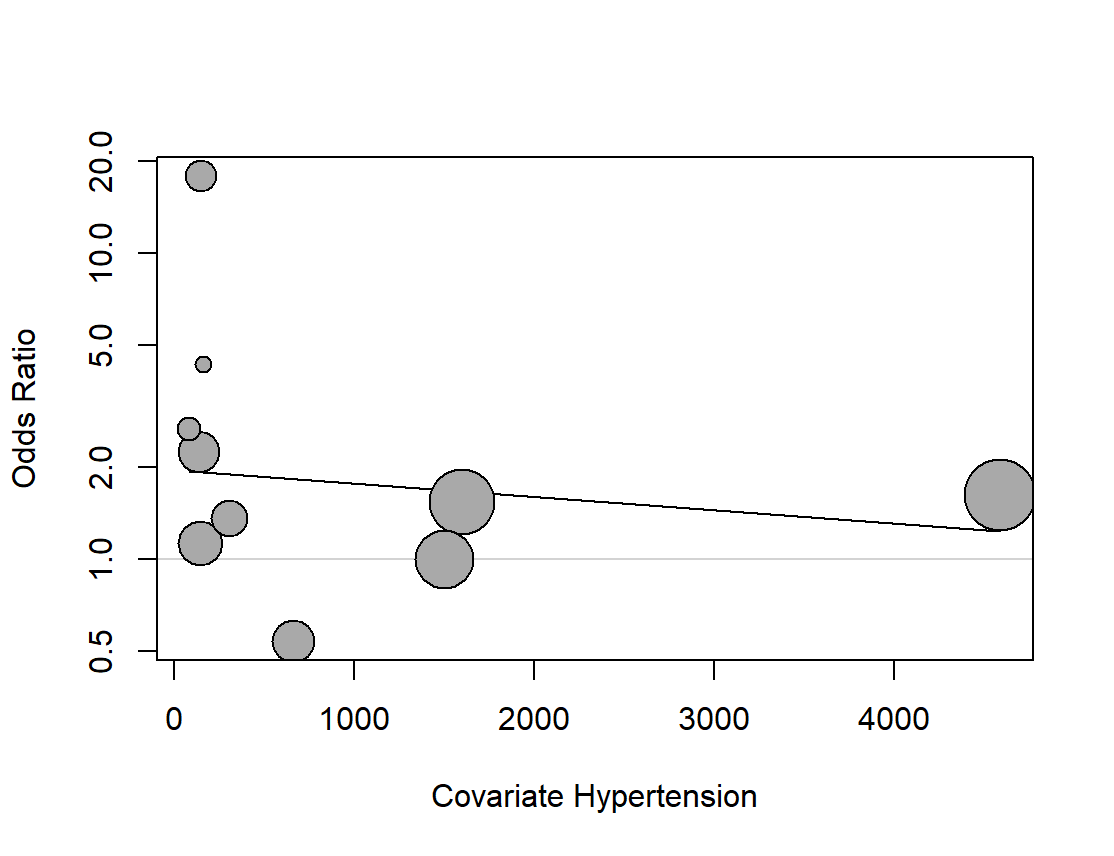


Figure S13_B. Bubble plots for univariate meta-regression for stroke at 3 years, covariate diabetes


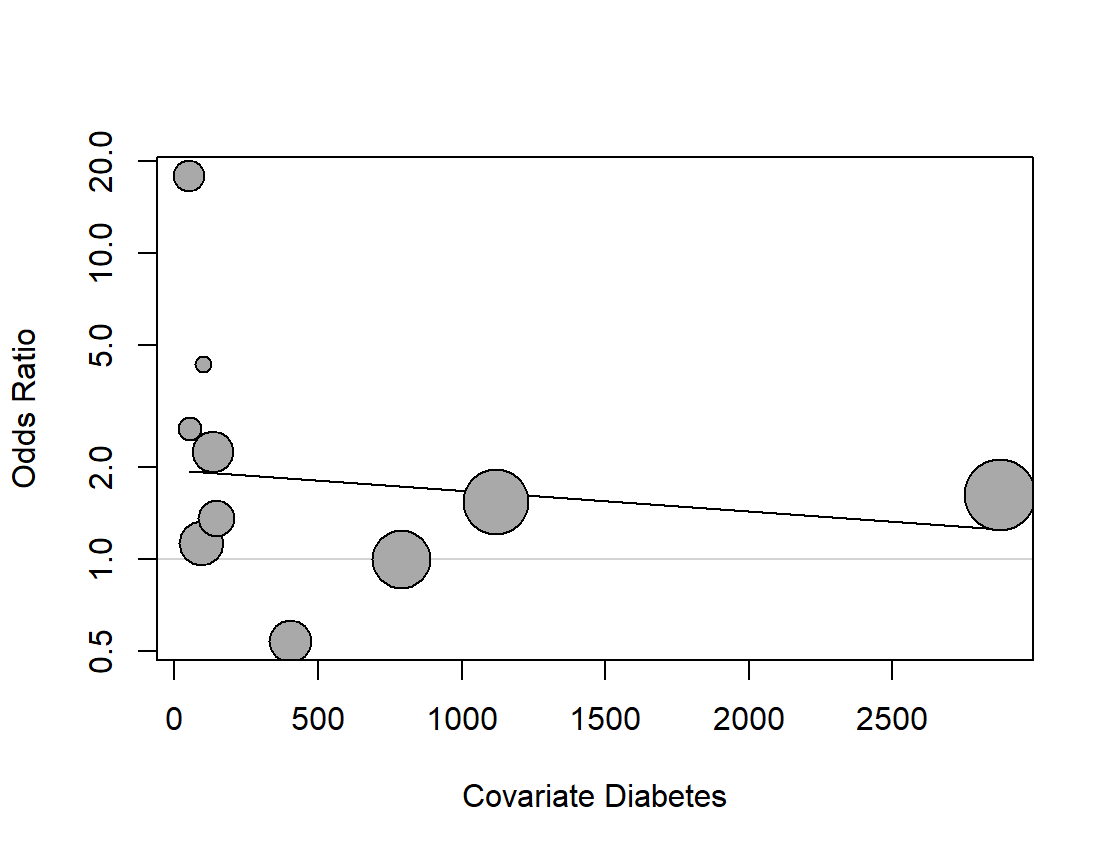


Figure S13_C. Bubble plots for univariate meta-regression for stroke at 3 years, covariate dialysis


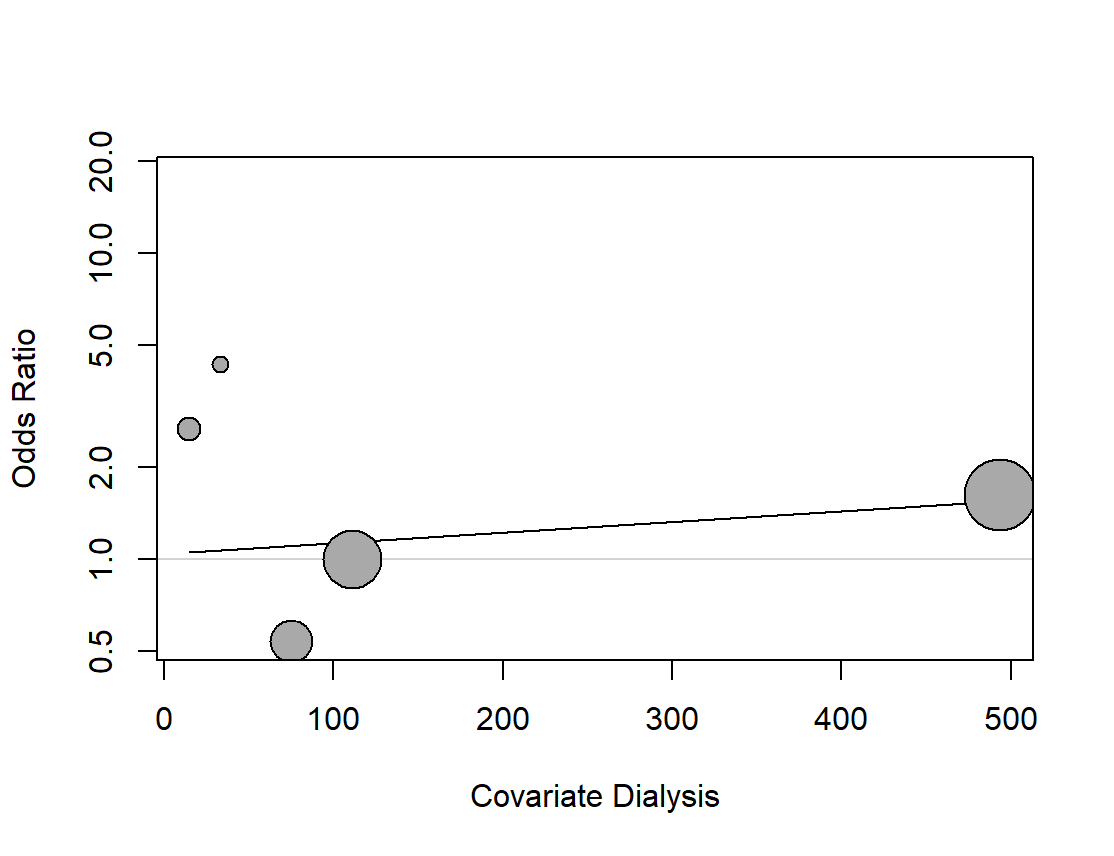


Figure S13_D. Bubble plots for univariate meta-regression for stroke at 3 years, covariate study type


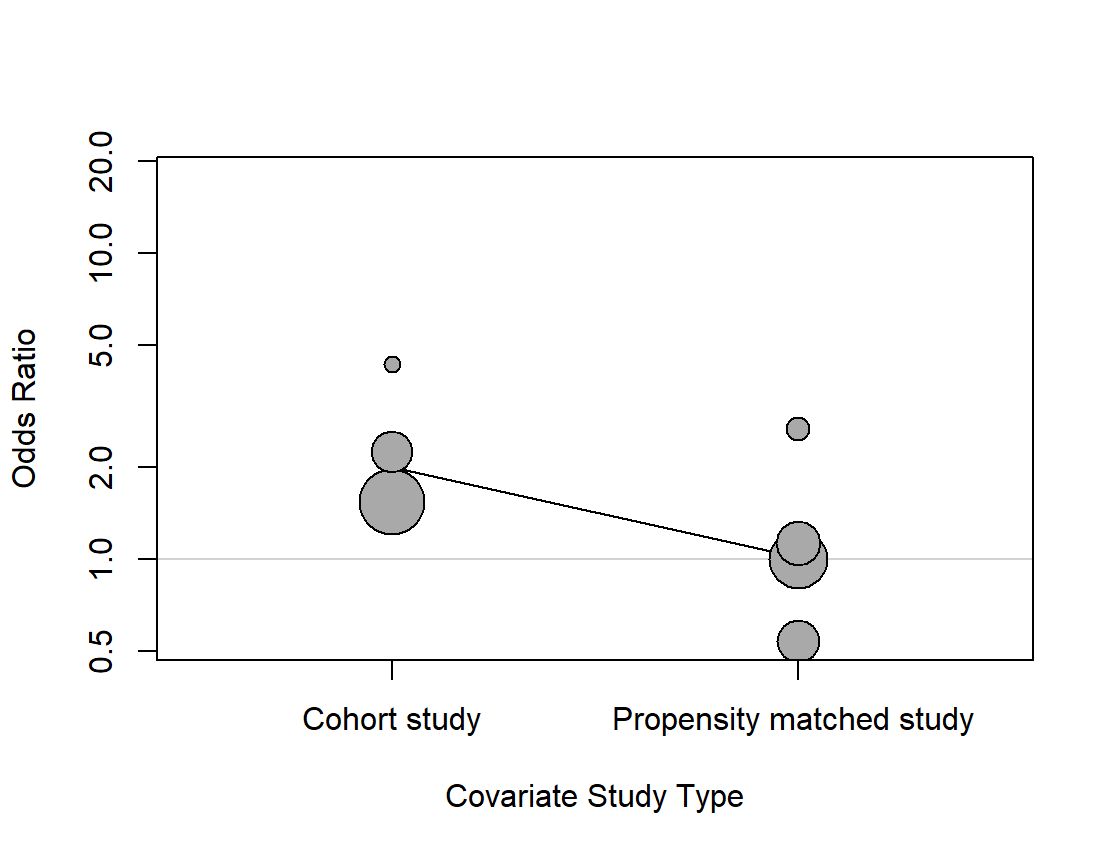


Figure S13_E. Bubble plots for univariate meta-regression for stroke at 3 years, covariate publication year


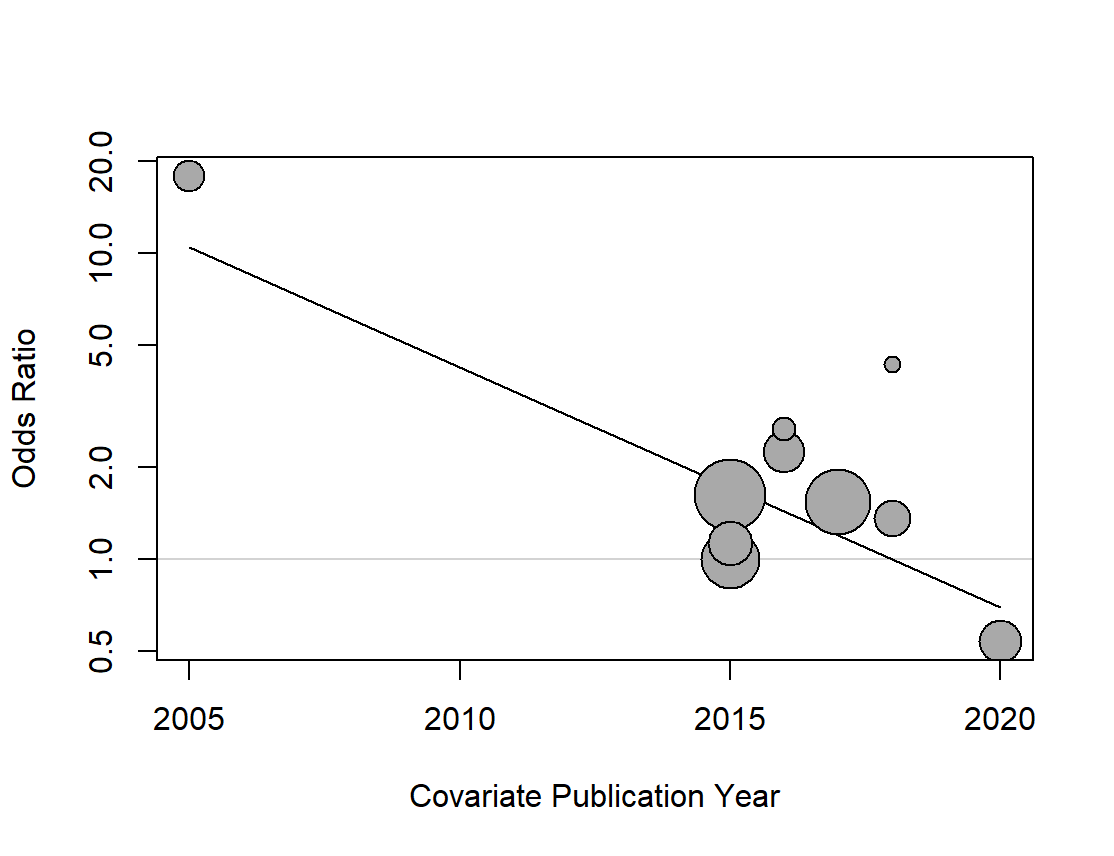


Figure S14. Forest plot subgroup meta-analysis of 30-days mortality for diabetes


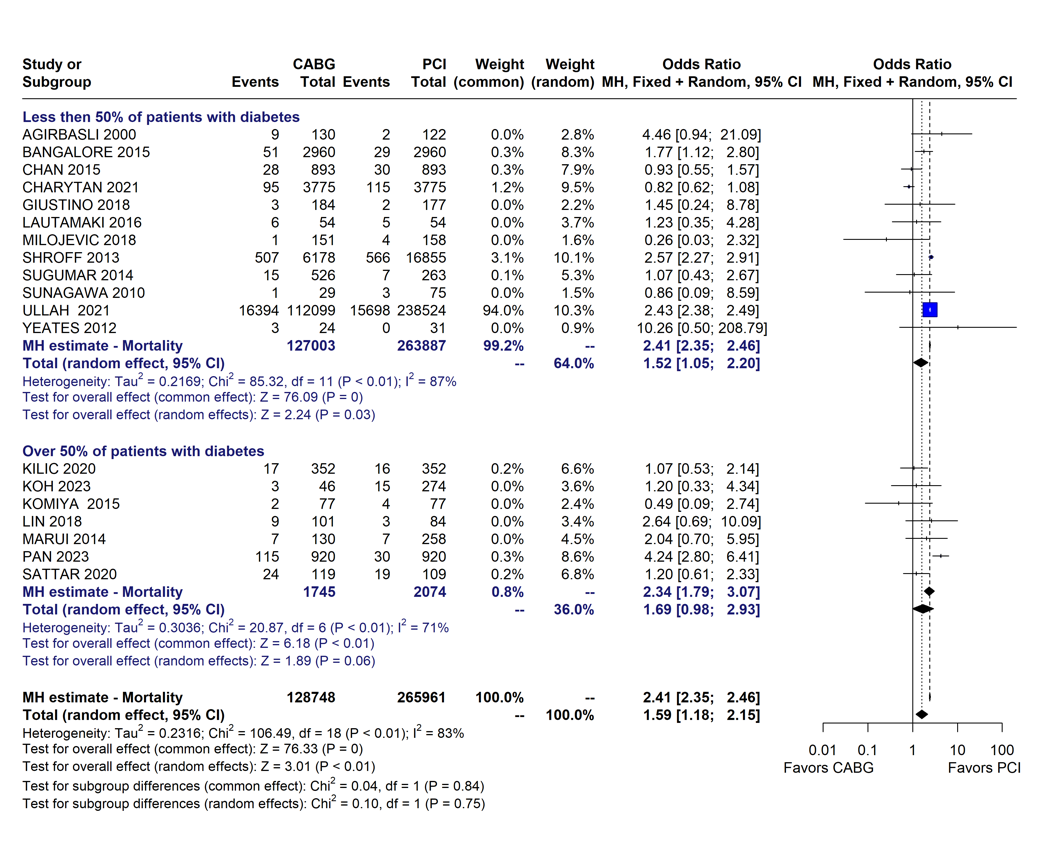


Figure S15. Forest plot subgroup meta-analysis of 30-days mortality for hemodialysis


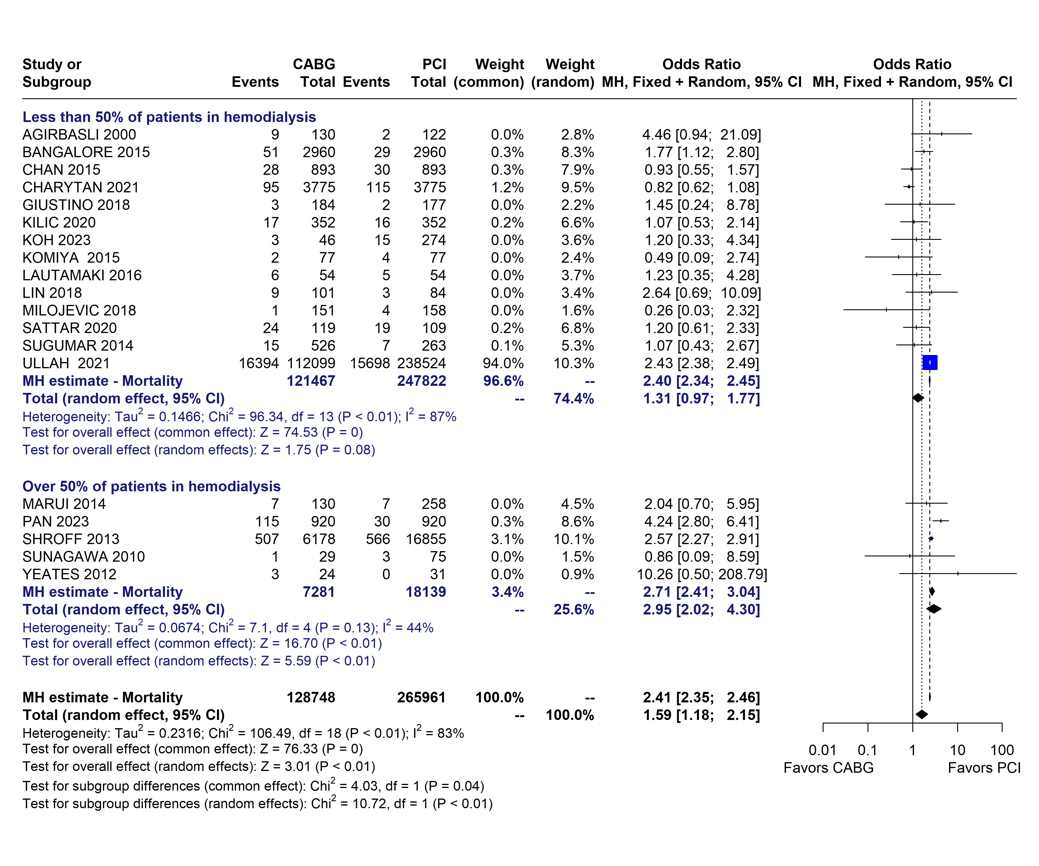


Figure S16. Forest plot subgroup meta-analysis of 30-days mortality for hypertension


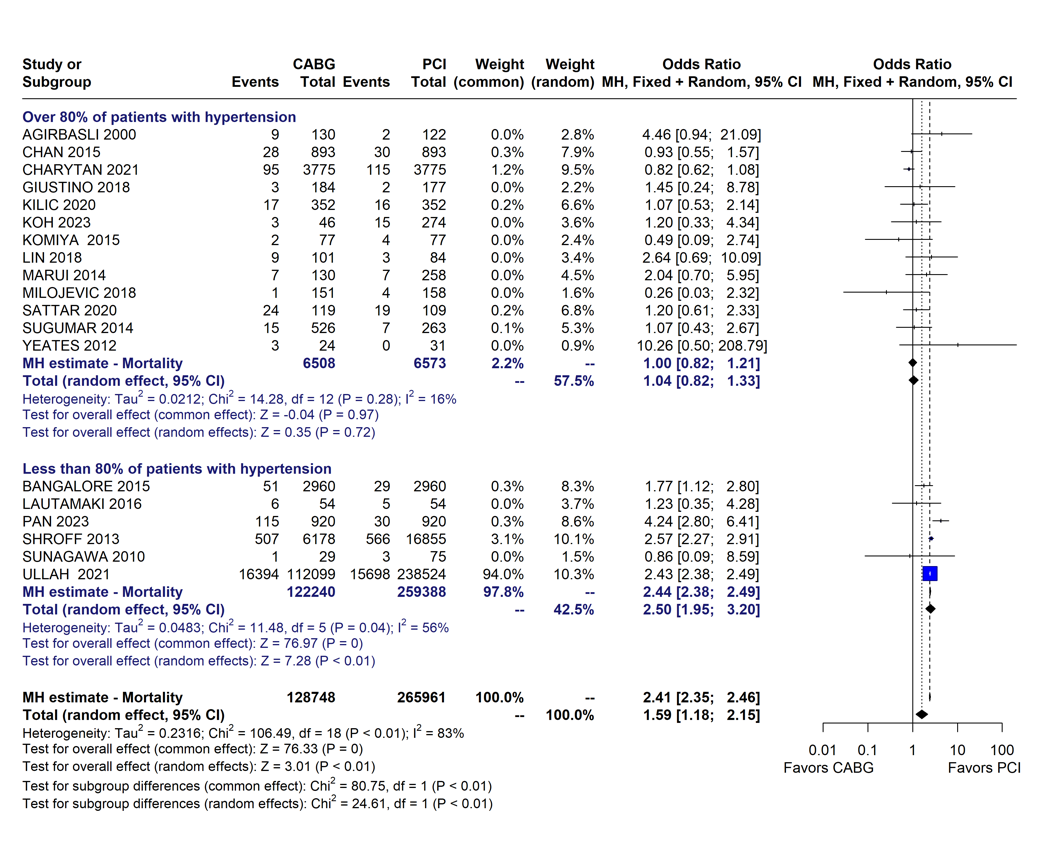


Figure S17. Forest plot subgroup meta-analysis of 30-days mortality for publication year


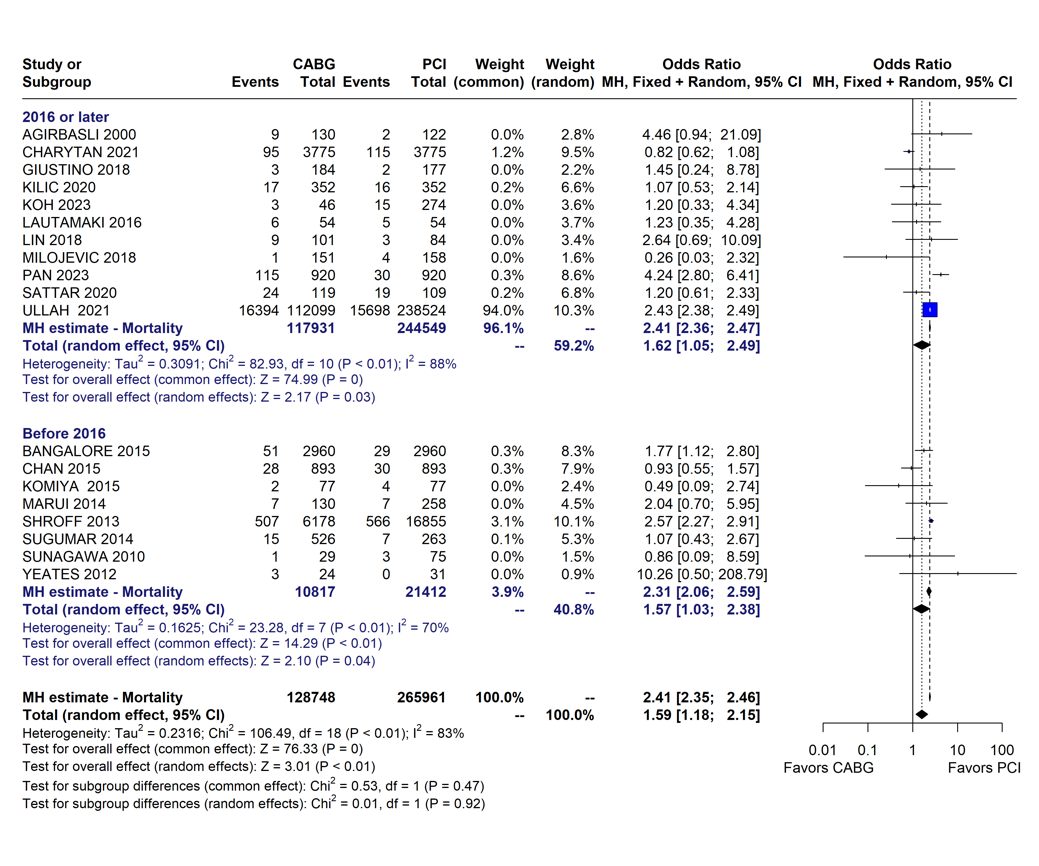


Figure S18. Forest plot subgroup meta-analysis of 30-days mortality for study type


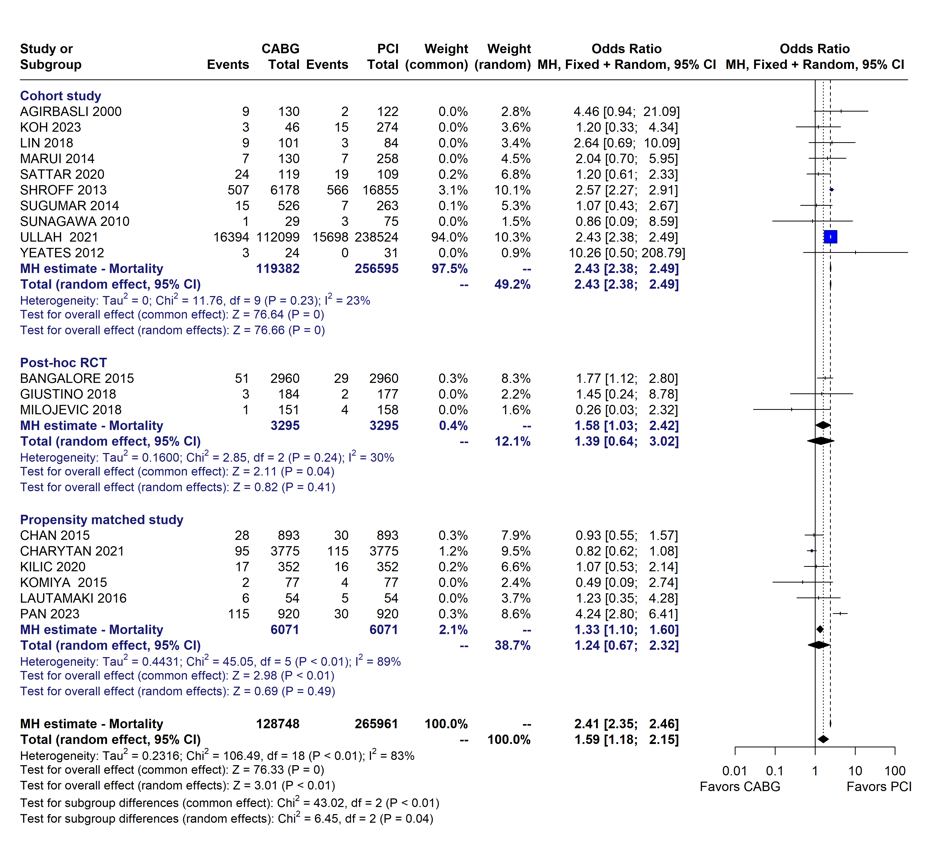


Figure S19. Forest plot subgroup meta-analysis of stroke for diabetes


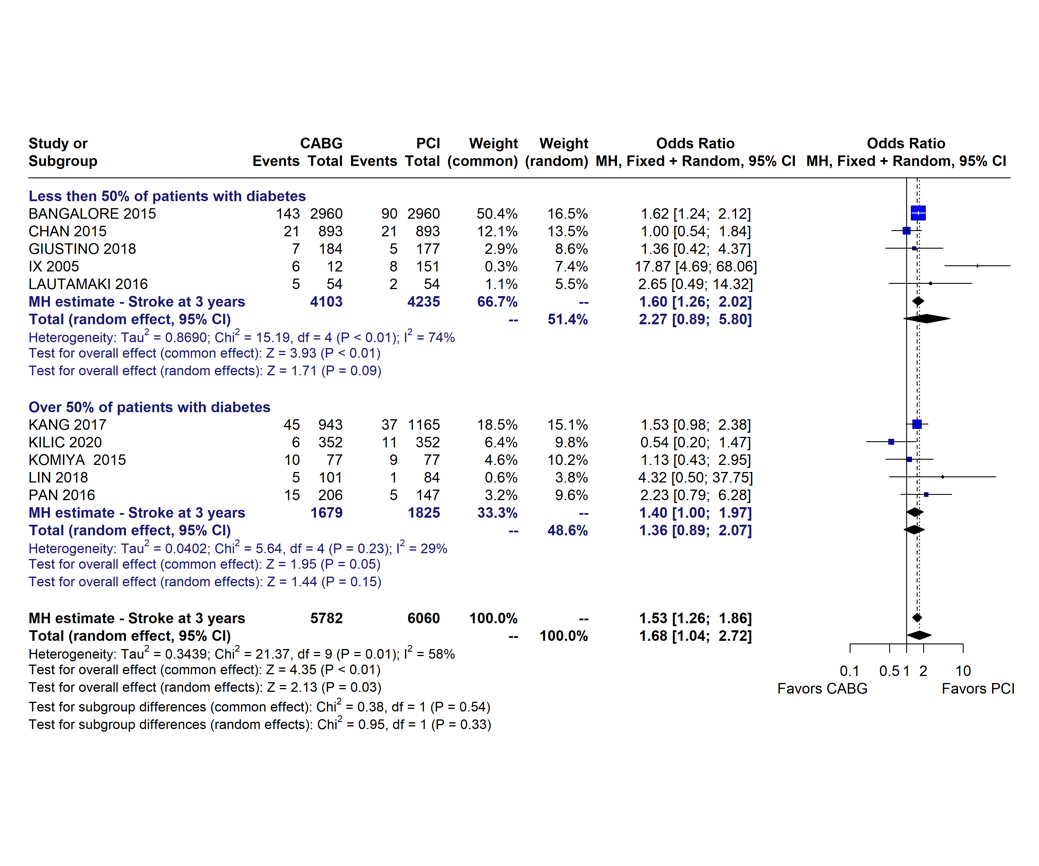


Figure S20. Forest plot subgroup meta-analysis of stroke for hemodialysis


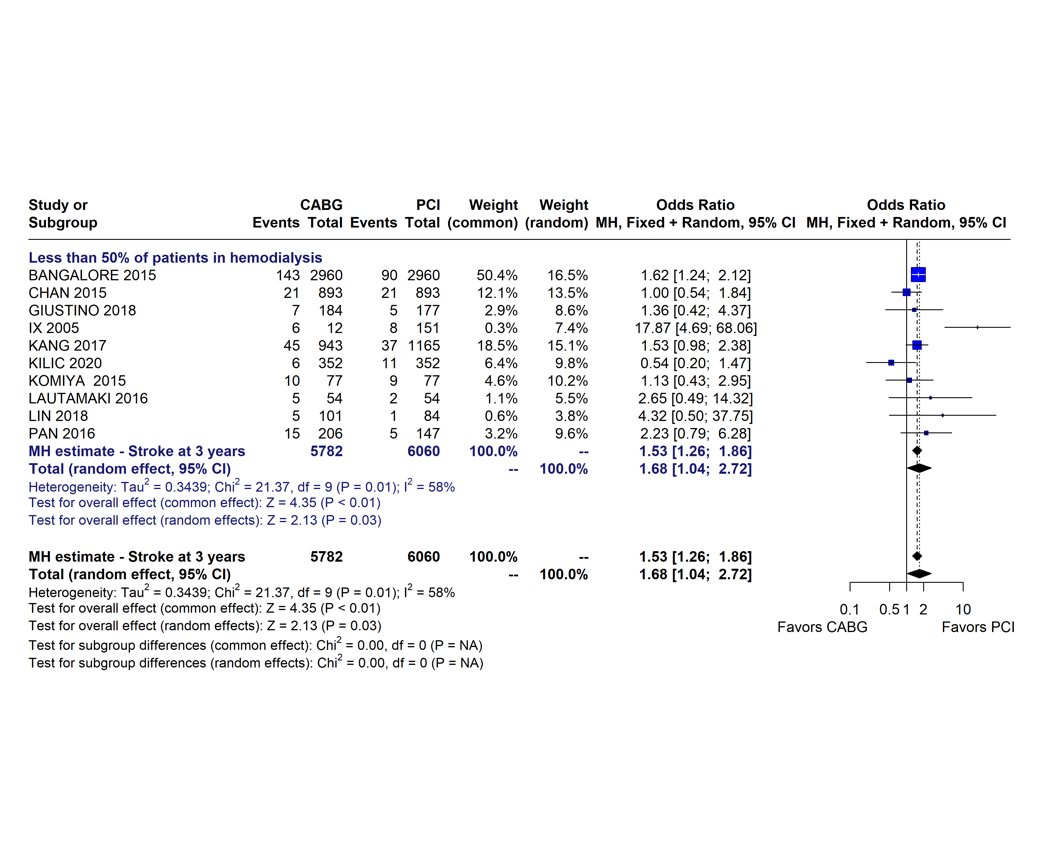


Figure S21. Forest plot subgroup meta-analysis of stroke for hypertension


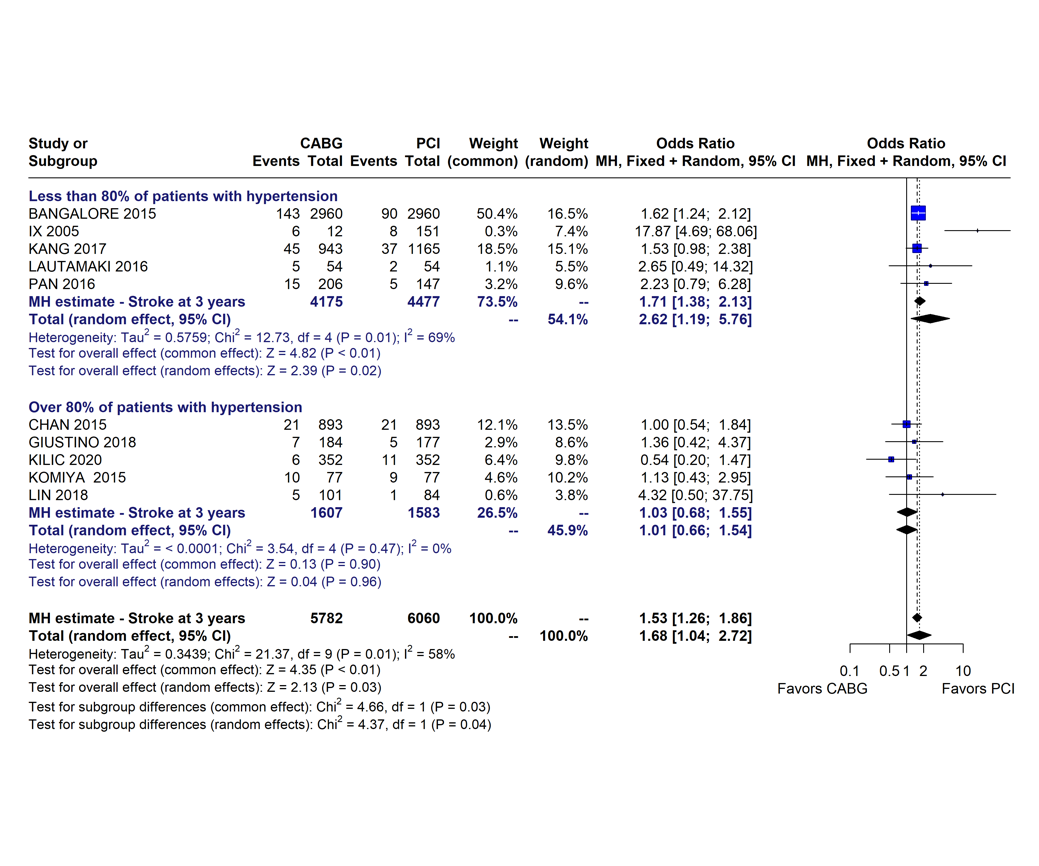


Figure S22. Forest plot subgroup meta-analysis of stroke for publication year


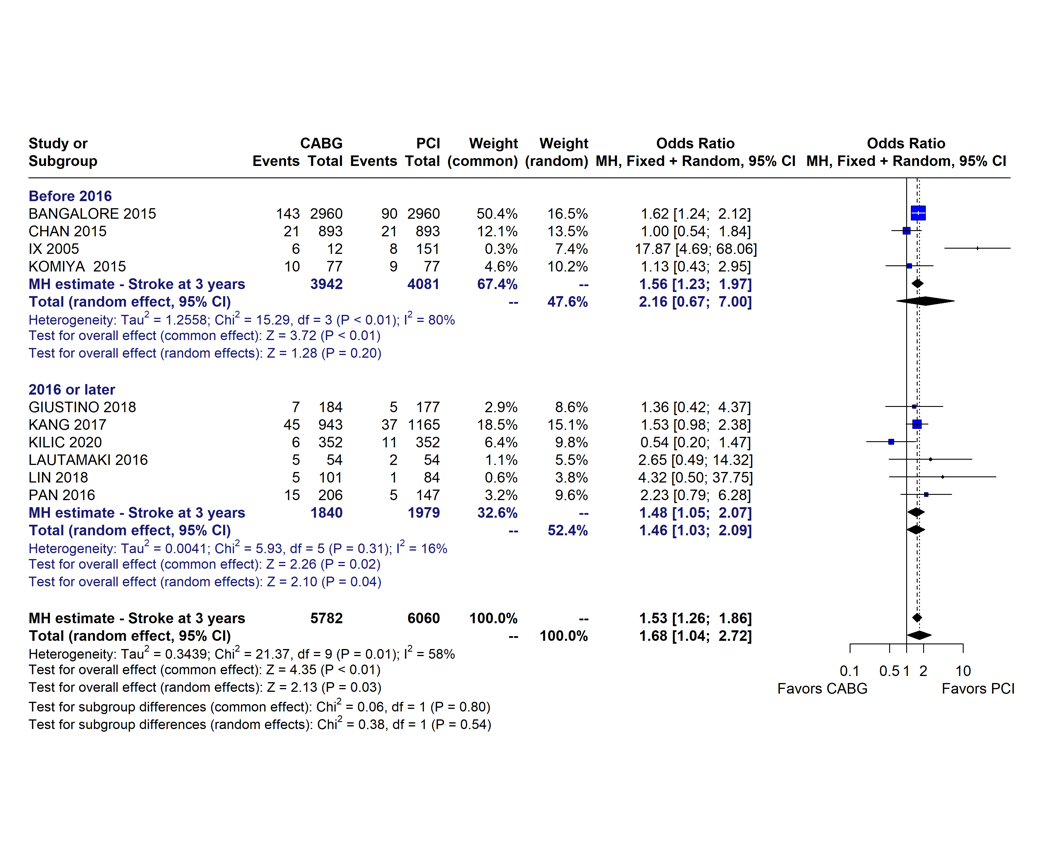


Figure S23. Forest plot subgroup meta-analysis of stroke for study type


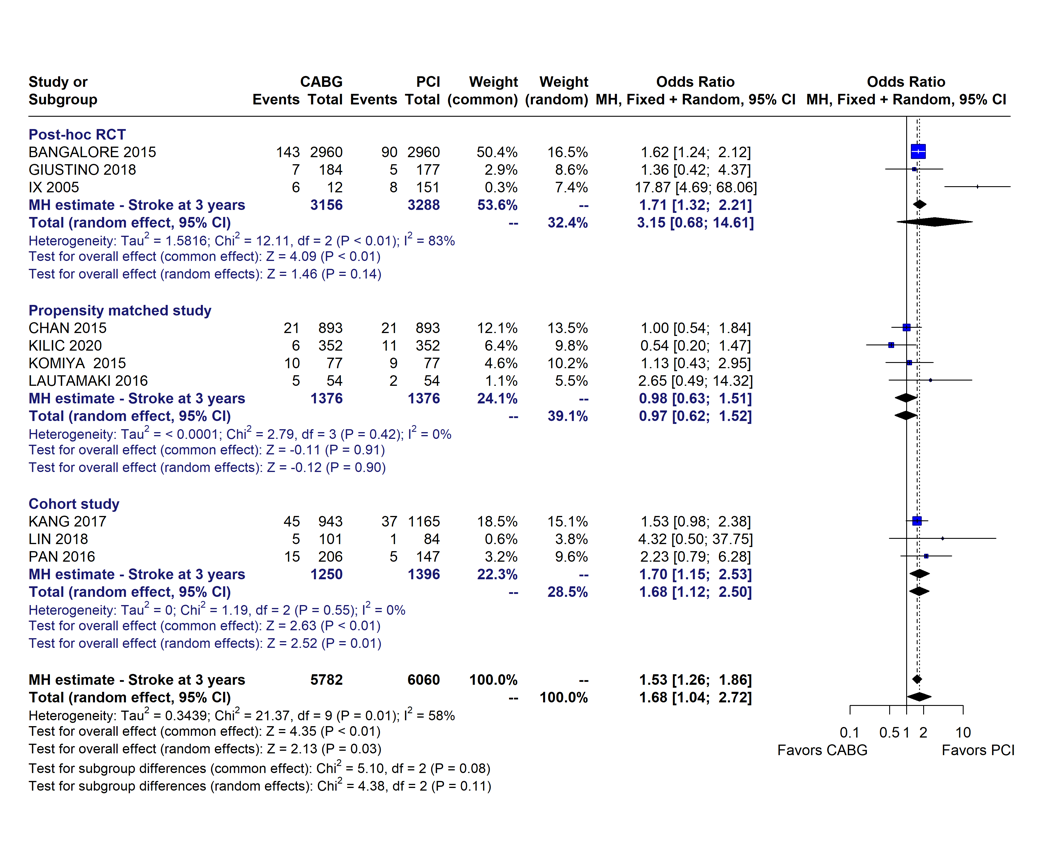


Figure S24. Forest plot subgroup meta-analysis of myocardial infarction for diabetes


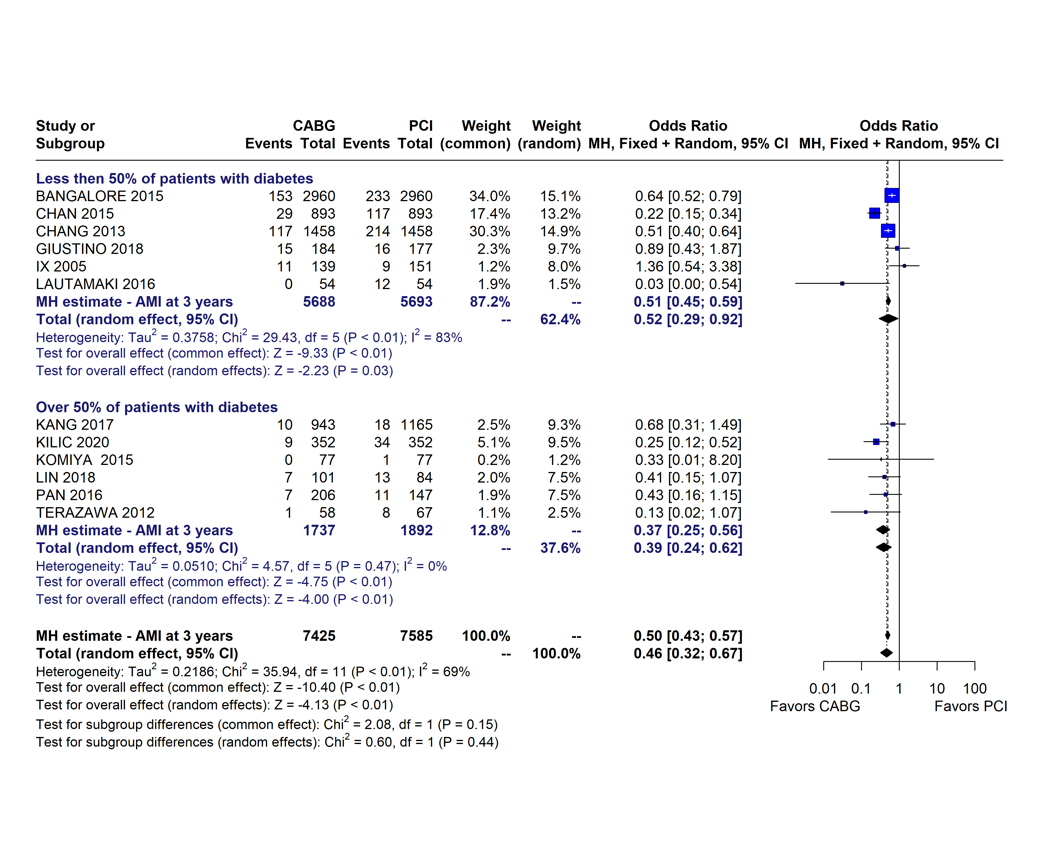


Figure S25. Forest plot subgroup meta-analysis of myocardial infarction for hemodialysis


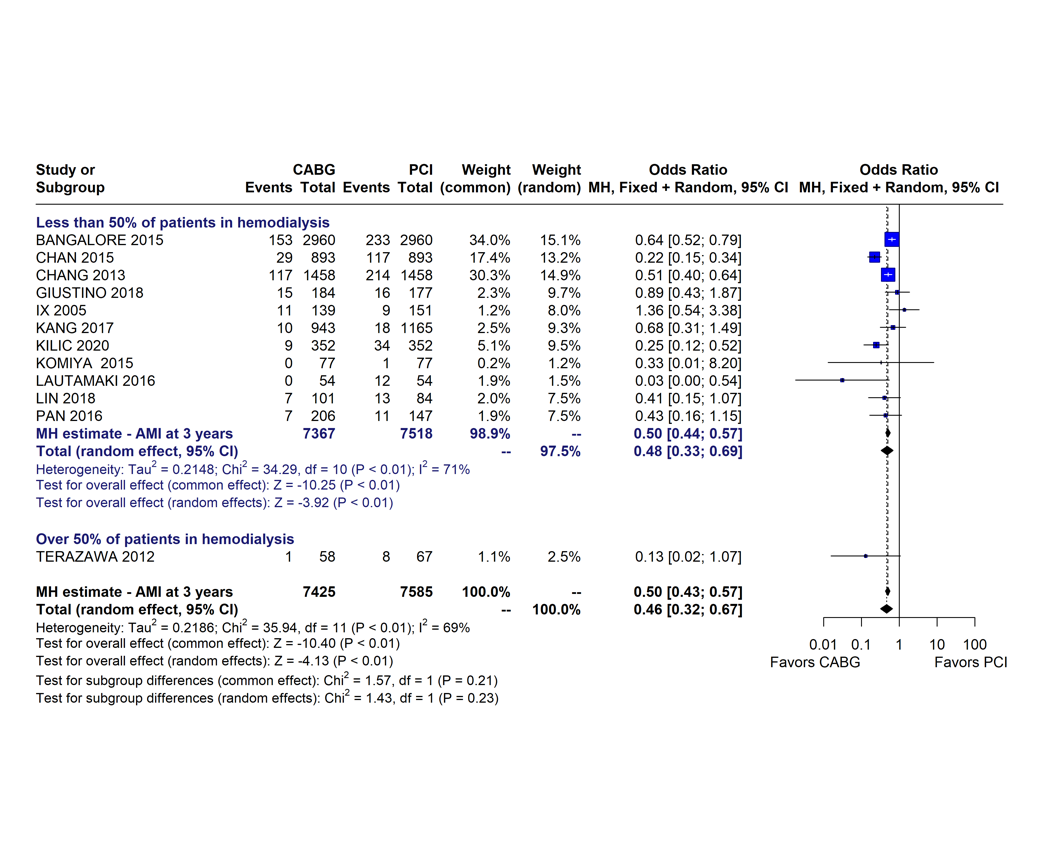


Figure S26. Forest plot subgroup meta-analysis of myocardial infarction for hypertension


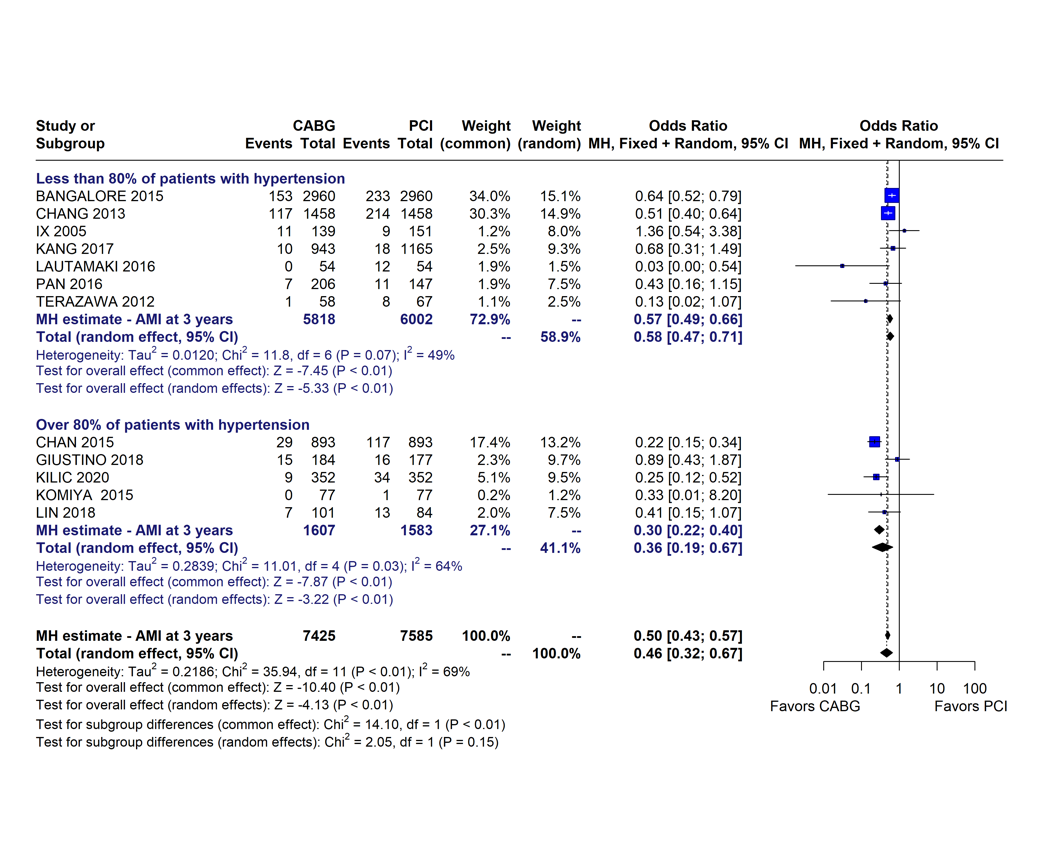


Figure S27. Forest plot subgroup meta-analysis of myocardial infarction for publication year


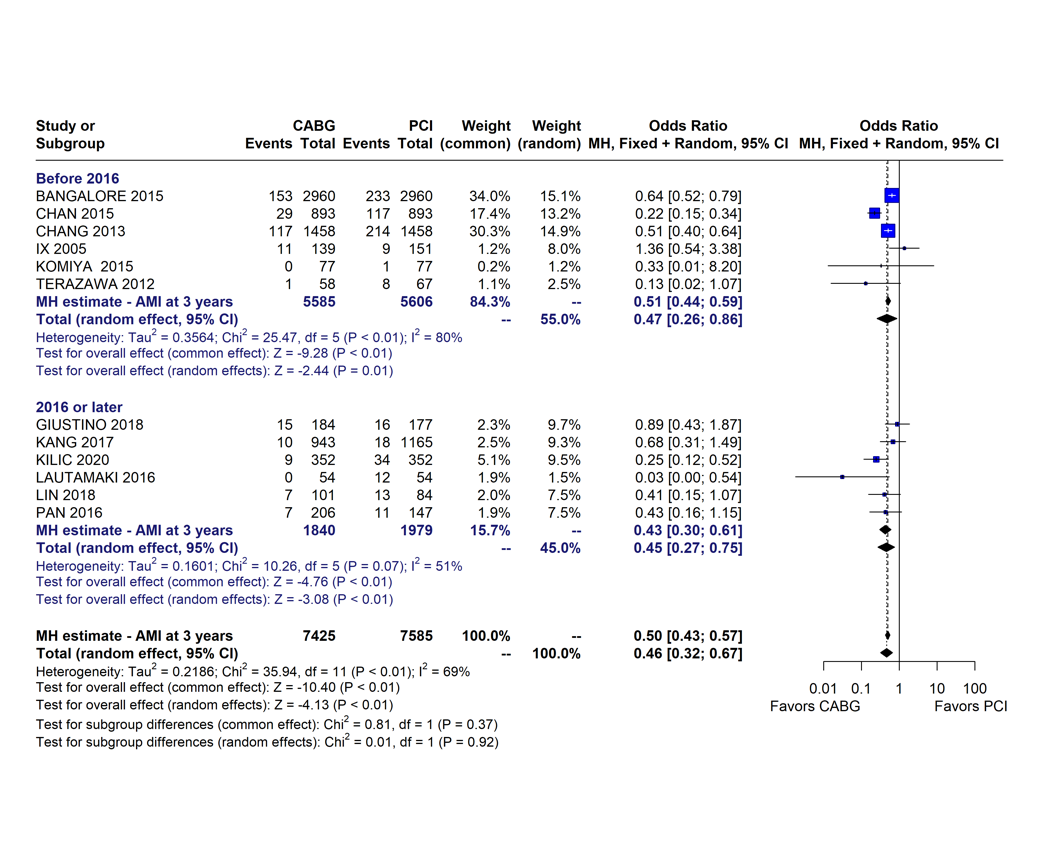


Figure S28. Forest plot subgroup meta-analysis of myocardial infarction for study type


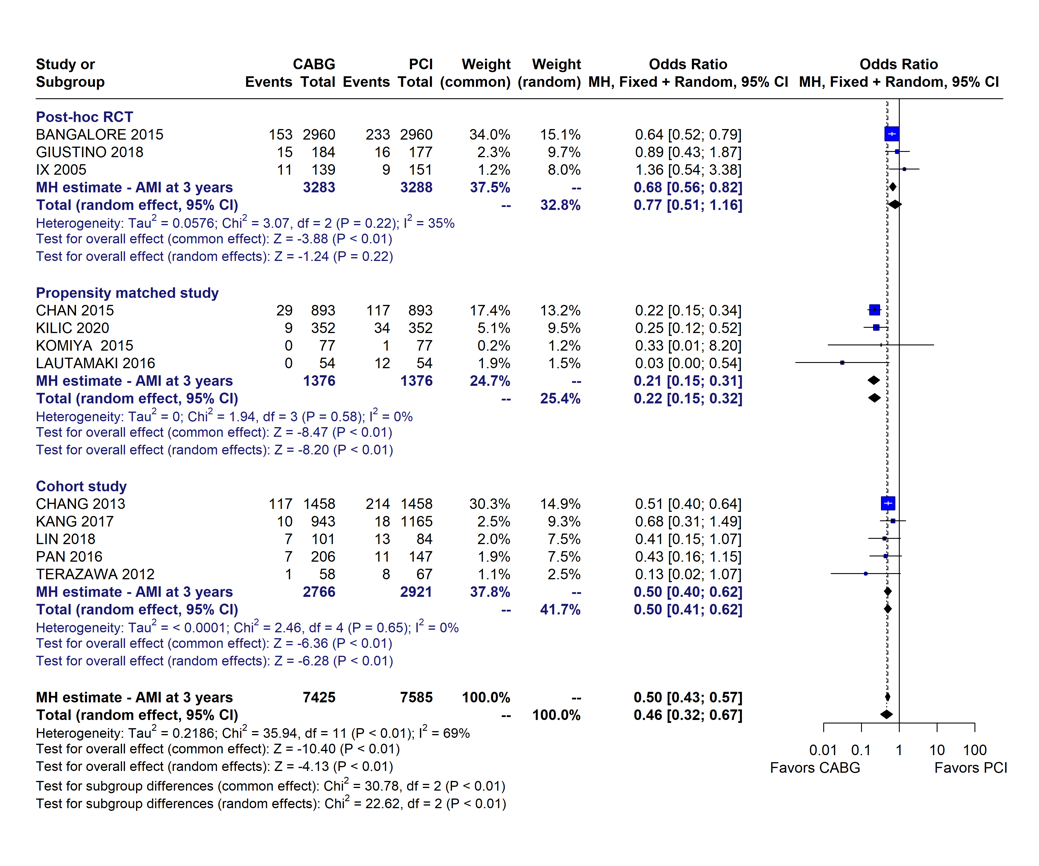


Figure S29. Forest plot subgroup meta-analysis of repeat revascularization for diabetes


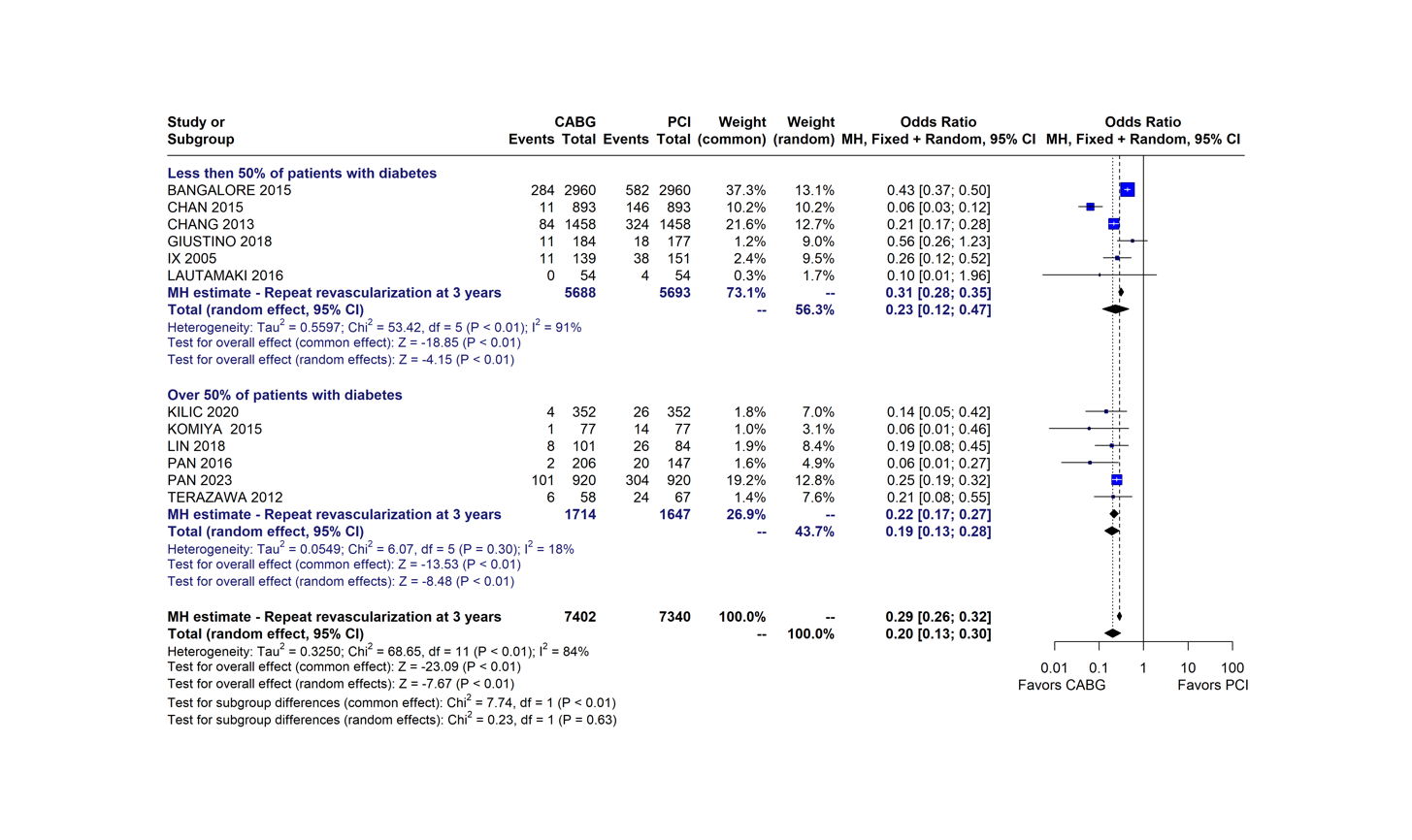


Figure S30. Forest plot subgroup meta-analysis of repeat revascularization for hemodialysis


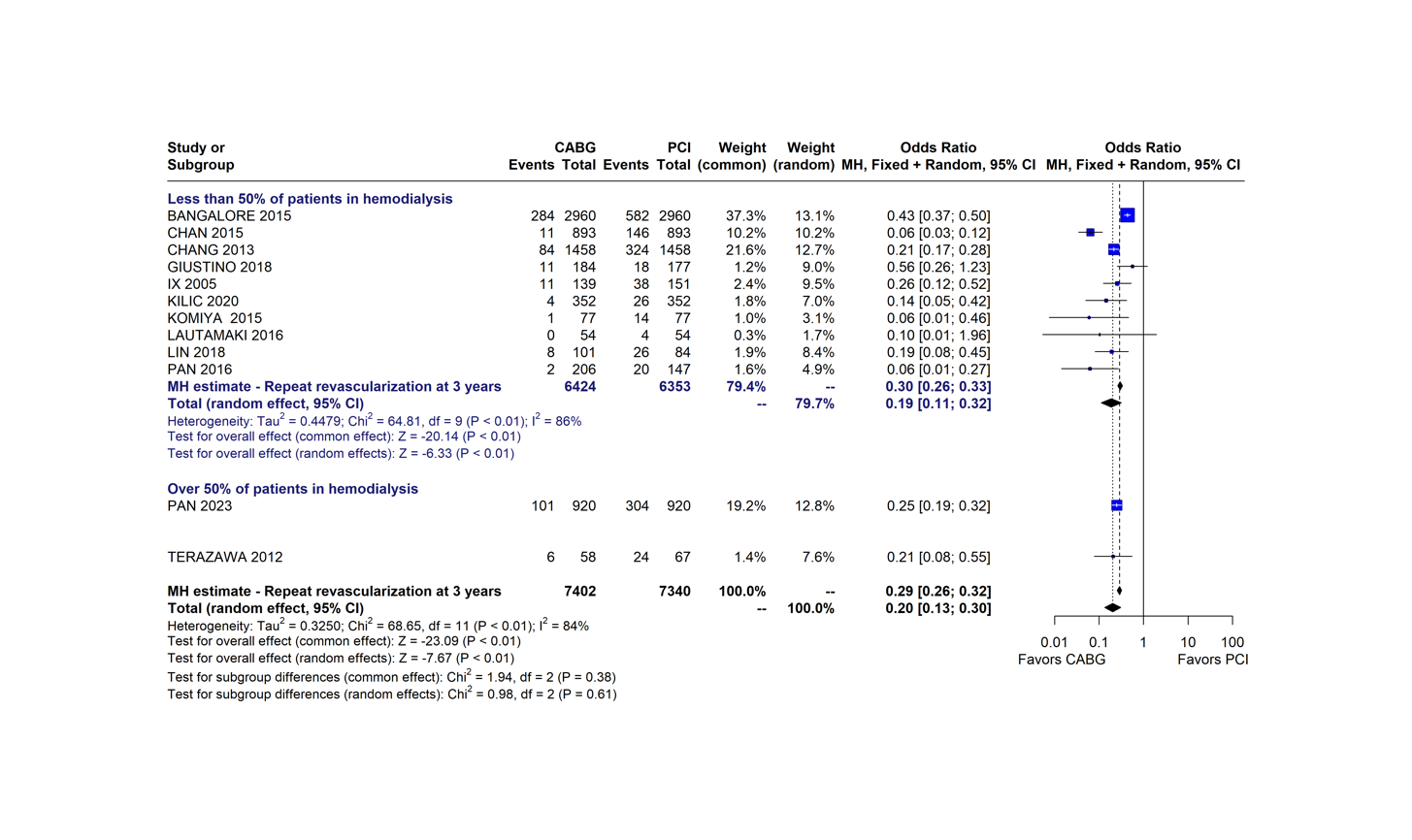


Figure S31. Forest plot subgroup meta-analysis of repeat revascularization for hypertension


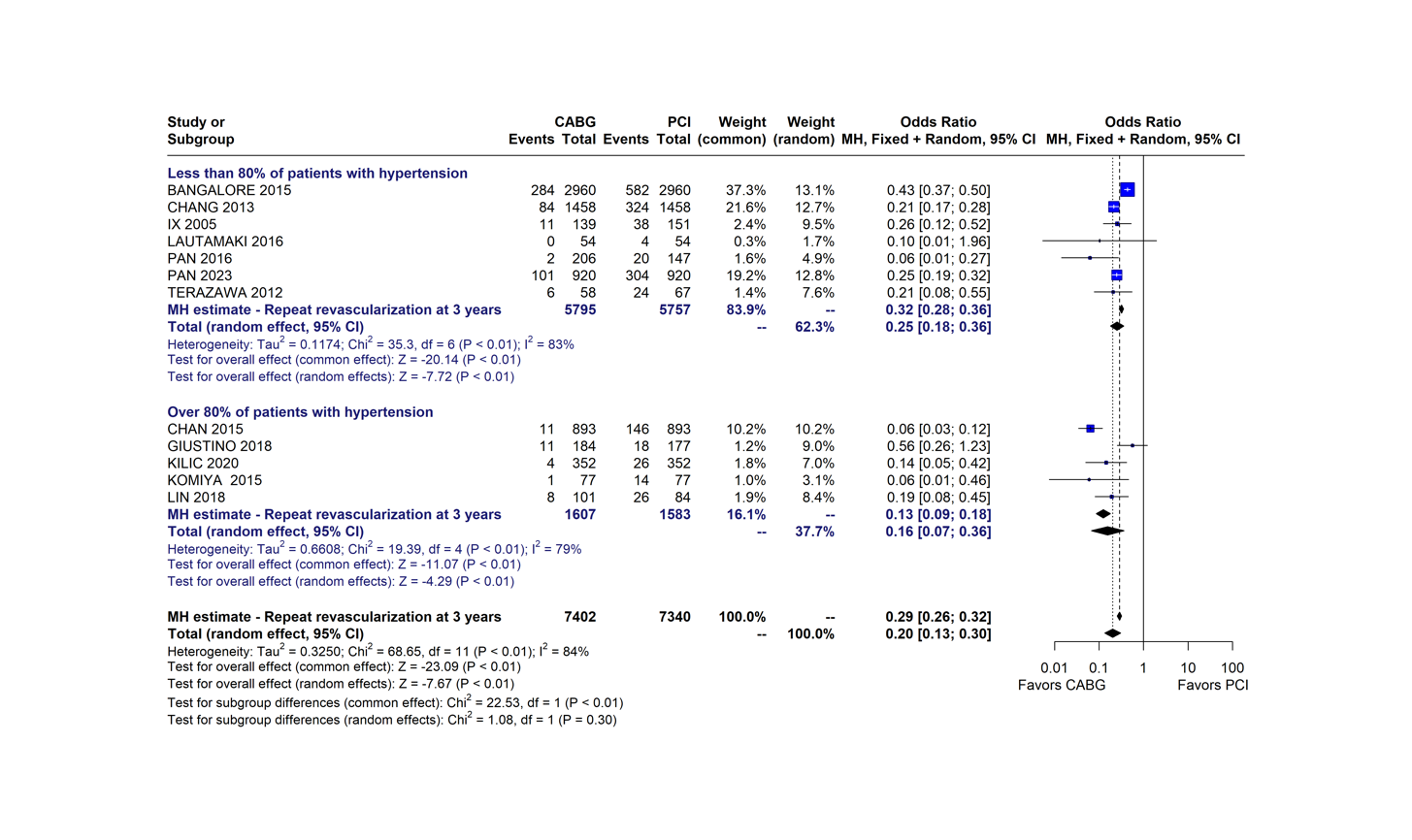


Figure S32. Forest plot subgroup meta-analysis of repeat revascularization for publication year


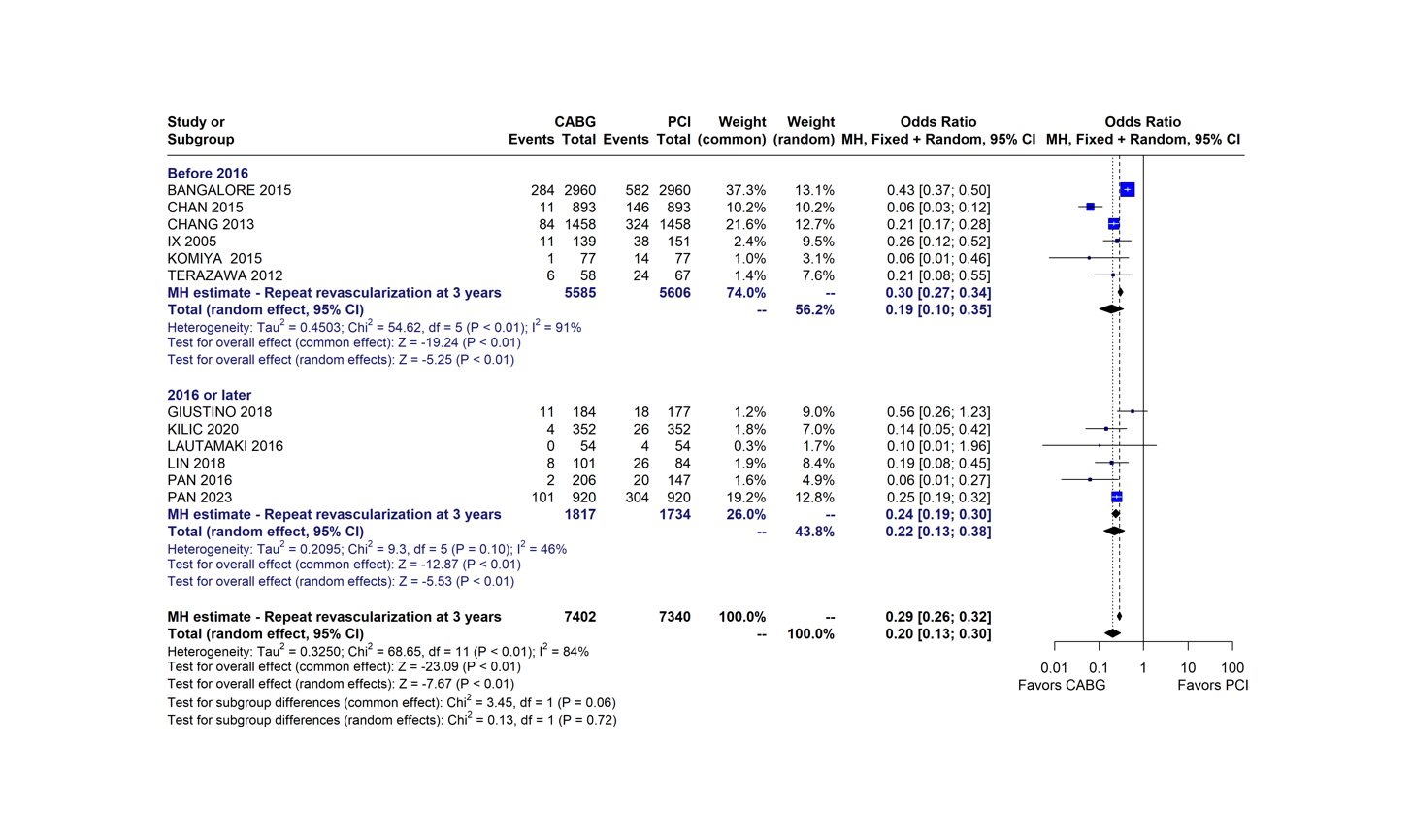


Figure S33. Forest plot subgroup meta-analysis of repeat revascularization for study type


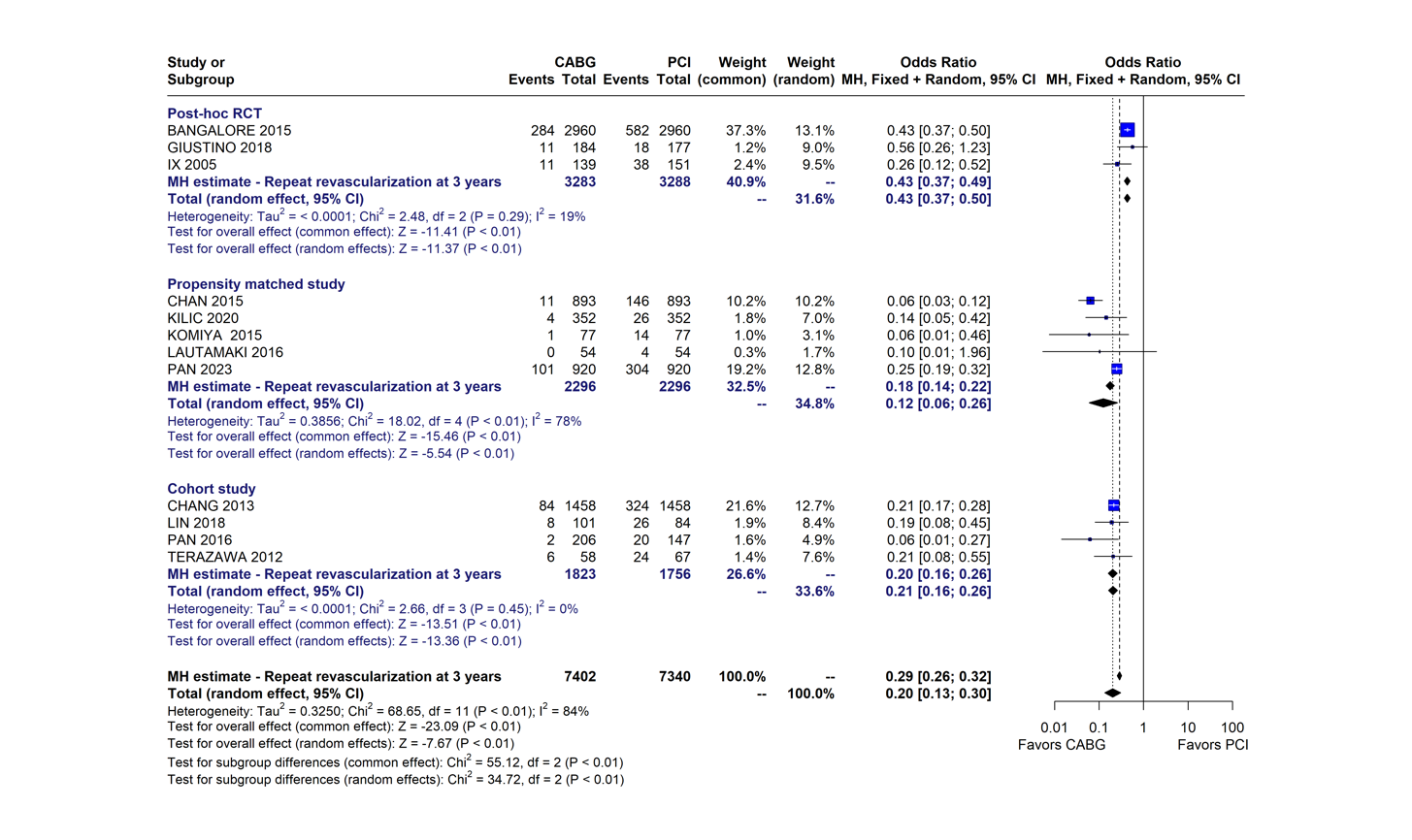


Table S3. Quality assessment of the included studies.

| **Study (Author/Year)** | **Newcastle-Ottawa Scale^111^** | | | | | | | | |
| --- | --- | --- | --- | --- | --- | --- | --- | --- | --- |
|  | **Representativeness** | **Selection** | **Ascertainment of exposure** | **Outcome of interest was not present at start of study** | **Comparability** | **Assessment of outcome** | **Follow-up long enough** | **Adeguacy of follow-up** | **Quality score** |
| Agirbasli, 2020 | **🟋** | **🟋** | **🟋** | **🟋** | **🟋🟋** | **🟋** | **🟋** | **🟋** | 9 |
| Aoki, 2005 | **🟋** | **🟋** | **🟋** | **🟋** | **🟋🟋** | **🟋** | **🟋** | **🟋** | 9 |
| Bangalore, 2015 | **🟋** | **🟋** | **🟋** | **🟋** | **🟋🟋** | **🟋** | **🟋** | **🟋** | 9 |
| Chan, 2015 | **🟋** | **🟋** | **🟋** | **🟋** | **🟋🟋** | **🟋** | **🟋** | **🟋** | 9 |
| Chang, 2013 | **🟋** | **🟋** | **🟋** | **🟋** | **🟋🟋** | **🟋** | **🟋** | **🟋** | 9 |
| Charytan, 2021 | **🟋** | **🟋** | **🟋** | **🟋** | **🟋🟋** | **🟋** | **🟋** | **🟋** | 9 |
| Giustino, 2018 | **🟋** | **🟋** | **🟋** | **🟋** | **🟋🟋** | **🟋** | **🟋** | **🟋** | 9 |
| Ix, 2005 | **🟋** | **🟋** | **🟋** | **🟋** | **🟋🟋** | **🟋** | **🟋** | **🟋** | 9 |
| Kang, 2017 | **🟋** | **🟋** | **🟋** | **🟋** | **🟋🟋** | **🟋** | **🟋** | **🟋** | 9 |
| Kilic, 2020 | **🟋** | **🟋** | **🟋** | **🟋** | **🟋🟋** | **🟋** | **🟋** | **🟋** | 9 |
| Kim, 2020 | **🟋** | **🟋** | **🟋** | **🟋** | **🟋🟋** | **🟋** | **🟋** | **🟋** | 9 |
| Koh, 2023 | **🟋** | **🟋** | **🟋** | **🟋** | **🟋🟋** | **🟋** | **🟋** | **🟋** | 9 |
| Komiya, 2015 | **🟋** | **🟋** | **🟋** | **🟋** | **🟋🟋** | **🟋** | **🟋** | **🟋** | 9 |
| Kumada, 2018 | **🟋** | **🟋** | **🟋** | **🟋** | **🟋🟋** | **🟋** | **🟋** | **🟋** | 9 |
| Lautamaki, 2016 | **🟋** | **🟋** | **🟋** | **🟋** | **🟋🟋** | **🟋** | **🟋** | **🟋** | 9 |
| Lima, 2016 | **🟋** | **🟋** | **🟋** | **🟋** | **🟋🟋** | **🟋** | **🟋** | **🟋** | 9 |
| Lin, 2018 | **🟋** | **🟋** | **🟋** | **🟋** | **🟋🟋** | **🟋** | **🟋** | **🟋** | 9 |
| Lopes, 2009 | **🟋** | **🟋** | **🟋** | **🟋** | **🟋🟋** | **🟋** | **🟋** | **🟋** | 9 |
| Manabe, 2009 | **🟋** | **🟋** | **🟋** | **🟋** | **🟋🟋** | **🟋** | **🟋** | **🟋** | 9 |
| Marui, 2014 | **🟋** | **🟋** | **🟋** | **🟋** | **🟋🟋** | **🟋** | **🟋** | **🟋** | 9 |
| Milojevic, 2018 | **🟋** | **🟋** | **🟋** | **🟋** | **🟋🟋** | **🟋** | **🟋** | **🟋** | 9 |
| Pan, 2016 | **🟋** | **🟋** | **🟋** | **🟋** | **🟋🟋** | **🟋** | **🟋** | **🟋** | 9 |
| Pan, 2023 | **🟋** | **🟋** | **🟋** | **🟋** | **🟋🟋** | **🟋** | **🟋** | **🟋** | 9 |
| Pilmore, 2017 | **🟋** | **🟋** | **🟋** | **🟋** | **🟋🟋** | **🟋** | **🟋** | **🟋** | 9 |
| Sattar, 2020 | **🟋** | **🟋** | **🟋** | **🟋** | **🟋🟋** | **🟋** | **🟋** | **🟋** | 9 |
| Shroff, 2013 | **🟋** | **🟋** | **🟋** | **🟋** | **🟋🟋** | **🟋** | **🟋** | **🟋** | 9 |
| Sugumar, 2014 | **🟋** | **🟋** | **🟋** | **🟋** | **🟋🟋** | **🟋** | **🟋** | **🟋** | 9 |
| Sunagawa, 2010 | **🟋** | **🟋** | **🟋** | **🟋** | **🟋🟋** | **🟋** | **🟋** | **🟋** | 9 |
| Terazawa, 2012 | **🟋** | **🟋** | **🟋** | **🟋** | **🟋🟋** | **🟋** | **🟋** | **🟋** | 9 |
| Ullah, 2021 | **🟋** | **🟋** | **🟋** | **🟋** | **🟋🟋** | **🟋** | **🟋** | **🟋** | 9 |
| Wang, 2020 | **🟋** | **🟋** | **🟋** | **🟋** | **🟋🟋** | **🟋** | **🟋** | **🟋** | 9 |
| Yeates, 2012 | **🟋** | **🟋** | **🟋** | **🟋** | **🟋🟋** | **🟋** | **🟋** | **🟋** | 9 |
| Zhang, 2016 | **🟋** | **🟋** | **🟋** | **🟋** | **🟋🟋** | **🟋** | **🟋** | **🟋** | 9 |
